# Naphthyl-Substituted Indole and Pyrrole Carboxylic Acids as Effective Antibiotic Potentiators—Inhibitors of Bacterial Cystathionine γ-Lyase

**DOI:** 10.3390/ijms242216331

**Published:** 2023-11-15

**Authors:** Andrey S. Kuzovlev, Mikhail D. Zybalov, Andrey V. Golovin, Maxim A. Gureev, Mariia A. Kasatkina, Mikhail V. Biryukov, Albina R. Belik, Sergey A. Silonov, Maxim A. Yunin, Nailya A. Zigangirova, Vasiliy V. Reshetnikov, Yulia E. Isakova, Yuri B. Porozov, Roman A. Ivanov

**Affiliations:** 1 Translational Medicine Research Center, Sirius University of Science and Technology, Olympic Ave. 1, 354340 Sochi, Russia; zybalov.mikhail@gmail.com (M.D.Z.); kasatkina.ma@talantiuspeh.ru (M.A.K.); biryukov.mv@talantiuspeh.ru (M.V.B.); belik.ar@talantiuspeh.ru (A.R.B.); silonov.sa@talantiuspeh.ru (S.A.S.); yunin.ma@talantiuspeh.ru (M.A.Y.); reshetnikov.vv@talantiuspeh.ru (V.V.R.); isakova.ye@talantiuspeh.ru (Y.E.I.); ivanov.ra@talantiuspeh.ru (R.A.I.); 2 Faculty of Bioengineering and Bioinformatics, Lomonosov Moscow State University, 1/73 Leninskie gori St., 119234 Moscow, Russia; golovin.av@talantiuspeh.ru; 3 Laboratory of Bioinformatics, Center of AI and Information Technologies, Sirius University of Science and Technology, Olympic Ave. 1, 354340 Sochi, Russia; gureev_m_a@staff.sechenov.ru (M.A.G.); porozov_yu_b@staff.sechenov.ru (Y.B.P.); 4 Laboratory of Bio- and Chemoinformatics, Institute of Biodesign and Modeling of Complex Systems, I.M. Sechenov First Moscow State Medical University, 8/2 Trubetskaya, 119991 Moscow, Russia; 5 Faculty of Biology, Lomonosov Moscow State University, 1/12 Leninskie gori St., 119234 Moscow, Russia; 6 Laboratory of Structural Dynamics, Stability and Folding of Proteins, Institute of Cytology, Russian Academy of Sciences, 4 Tikhoretsky Ave., 194064 St. Petersburg, Russia; 7 Medical Microbiology Department, Laboratory of Chlamydiosis, National Research Center for Epidemiology and Microbiology Named after N. F. Gamaleya, 18 Gamaleya St., 123098 Moscow, Russia; zigangirova@mail.ru; 8 Institute of Cytology and Genetics, Siberian Branch of RAS, 10 Akademika Lavrentyeva, 630090 Novosibirsk, Russia

**Keywords:** potentiators, antibiotics, antibiotic resistance, naphthalene, indole, pyrrole, CSE, cystathionine γ-lyase, molecular docking, molecular dynamics

## Abstract

Over the past decades, the problem of bacterial resistance to most antibiotics has become a serious threat to patients’ survival. Nevertheless, antibiotics of a novel class have not been approved since the 1980s. The development of antibiotic potentiators is an appealing alternative to the challenging process of searching for new antimicrobials. Production of H_2_S—one of the leading defense mechanisms crucial for bacterial survival—can be influenced by the inhibition of relevant enzymes: bacterial cystathionine γ-lyase (bCSE), bacterial cystathionine β-synthase (bCBS), or 3-mercaptopyruvate sulfurtransferase (MST). The first one makes the main contribution to H_2_S generation. Herein, we present data on the synthesis, in silico analyses, and enzymatic and microbiological assays of novel bCSE inhibitors. Combined molecular docking and molecular dynamics analyses revealed a novel binding mode of these ligands to bCSE. Lead compound **2a** manifested strong potentiating activity when applied in combination with some commonly used antibiotics against multidrug-resistant *Acinetobacter baumannii*, *Pseudomonas aeruginosa*, and methicillin-resistant *Staphylococcus aureus*. The compound was found to have favorable in vitro absorption, distribution, metabolism, excretion, and toxicity parameters. The high effectiveness and safety of compound **2a** makes it a promising candidate for enhancing the activity of antibiotics against high-priority pathogens.

## 1. Introduction

The problem of the emergence and spread of bacterial antibiotic resistance is a growing public health problem. The WHO predicts that bacterial infections will claim around 10 million human lives each year [1]. Antibiotics of the “golden era” (the 1940s to the 1960s) and their various derivatives, which are still actively used in clinical practice, are becoming obsolete due to the decreasing vulnerability of bacteria [2]. Infections caused by such bacteria as *Enterococci faecalis*, *Enterococci faecium*, *Streptococcus pneumoniae*, *Clostridium difficile*, *Mycobacterium tuberculosis*, *Pseudomonas aeruginosa*, *Acinetobacter baumannii*, *Klebsiella pneumoniae*, *Enterobacter*, and *Neisseria gonorrhoeae* are difficult to treat with existing antimicrobials and therefore pose a great threat to humanity [3]. Currently, there is only a limited set of compounds actively used for antibacterial therapy and a few substances are in clinical development [4]. Almost all drugs approved for antibacterial treatment in the past three decades belong to already known classes, possessing the same core structure with a modified side chain that makes them less toxic to humans and slightly more effective against bacteria [5]. Hence, overcoming antibiotic resistance is a cutting-edge topic in the field of medicinal chemistry. There are three main strategies for addressing this problem: (1) the development of new antibiotics with fundamentally novel molecular structures [6]; (2) the modification of antibiotics from known classes by the variation of substituents in a key structural motif [7]; and (3) a search for new antibiotic potentiators (adjuvants), which are compounds that can be used together with antibiotics to block certain bacterial defense mechanisms through interference with metabolic routes inside the bacterial cell [8]. A lack of resistance to potentiators, selective action on diverse bacterial targets, a simple structure, and short synthetic pathways from readily accessible reagents make adjuvant screening an attractive alternative to the complicated, time-consuming, and expensive process of development of new antibiotics. According to their mechanism of action, potentiators are categorized into several classes: (1) compounds with weak antibacterial properties that show synergy with other antibiotics; (2) compounds that influence bacterial membrane permeability; and (3) compounds affecting specific targets inside the bacterial cell by obstructing the main physiological processes in the cell [9].

**Figure 1:**
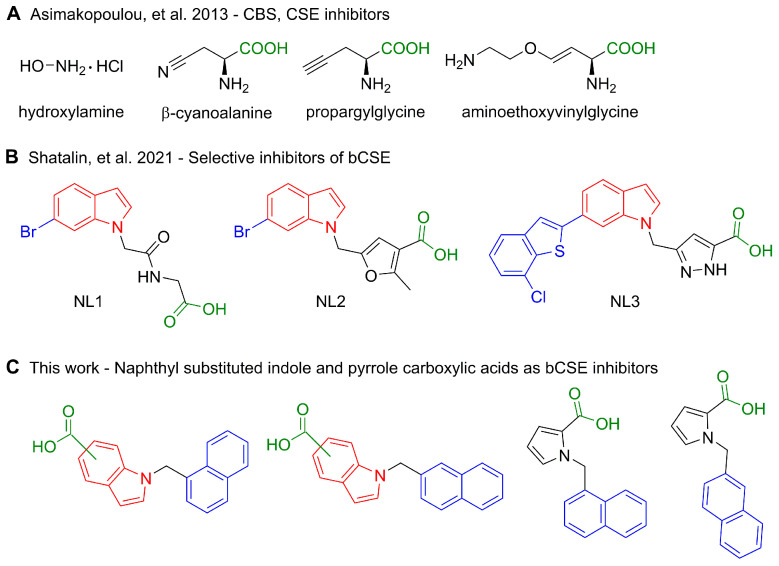
A summary of bacterial cystathionine γ-lyase (bCSE) inhibitors. (**A**) The earliest discovered potentiators [15]; (**B**) Indole-containing compounds–selective inhibitors of bCSE [13]; (**C**) Naphthyl substituted indole and pyrrole carboxylic acids as bCSE inhibitors.

**Figure 2:**
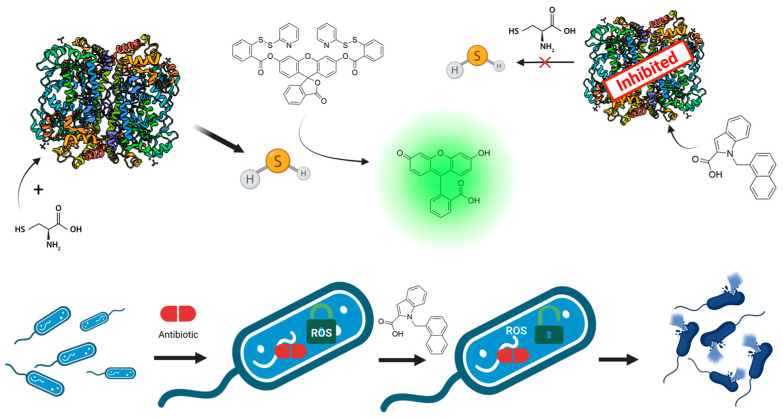
Graphical abstract of the research reported herein. In enzymatic assay, bCSE breaks down cysteine-producing H_2_S, which further reacts with WSP5, forming a fluorescent product. In the case of efficient bCSE inhibition (by our lead molecule) this reaction does not take place due to the absence of H_2_S. Antibiotic exposure leads to expression of DNA damage response and repair genes. It causes increased aerobic respiratory activity and oxidative stress [20,21]. Normally bacteria successfully cope with oxidation stress by H_2_S production. Nevertheless, in the presence of bCSE inhibitors, ROS formation results in destruction of bacterial cell.

One of the important bacterial defense mechanisms is the suppression of oxidative stress caused by the penetration of the cell membrane by an antibiotic [10]. A lot of harmful reactive oxygen species (ROS) are released during this process, damaging vital macromolecules and biological structures. To prevent this damage, bacteria produce reducing agents. Hydrogen sulfide (H_2_S) is one of these ROS scavengers. Genetic disruption of H_2_S production increases the sensitivity of pathogenic bacteria to a wide range of antibiotics [11]. Two pyridoxal-5′-phosphate–dependent enzymes are mainly responsible for the production of H_2_S in bacteria: bacterial cystathionine γ-lyase (bCSE) and bacterial cystathionine β-synthase (bCBS) [12]. Nonetheless, inactivation of the bCSE gene results in a considerable decrease in H_2_S production, whereas only a slight effect is observed with the absence of bCBS [13]. Mammals possess a similar system of orthologous enzymes detoxifying ROS [14]. Thus, promising antibiotic potentiators affecting oxidative stress defense need to be primarily specific to bCSE. A number of more or less specific bCSE inhibitors were published recently. The earliest discovered potentiators, such as hydroxylamine, β-cyanoalanine, propargylglycine, and aminoethoxyvinylglycine (Figure 1A), possess simple structures and are completely unselective toward bCSE [15]. Indole scaffold is a key structural motif of diverse bioactive compounds: anticancer [16], antiviral [17], and antifungal agents [18]. Recently, indole-containing compounds were found to be selective inhibitors of bCSE [13]. Although the application of these compounds in combination with bactericidal antibiotics allows for a reduction in antibiotic dosage, their synthetic route is quite long (seven steps) and includes complicated steps such as rhodium-catalyzed cyclization and Suzuki cross-coupling (Figure 1B). Here we report on readily synthesized (Figure 1C) selective inhibitors of bCSE that potentiate the activity of antibiotics from *β*-lactam (penicillin, cephalosporin, and carbapenem families), fluoroquinolone, and aminoglycoside classes against pathogenic bacteria of the ESKAPE group (Figure 2). Molecular docking and molecular dynamics analyses were used to elucidate the putative mechanism of action and possible bCSE-binding sites within these compounds. A reduction in enzymatically produced H_2_S was quantitatively assayed here by means of a 2-pyridyl disulfide–based fluorescent probe [19]. In vitro absorption, distribution, metabolism, excretion, and toxicity (ADMET) assays indicated a favorable profile of our lead compound.

## 2. Results and Discussion

### 2.1. Design Strategy and Synthesis

**Figure 3:**
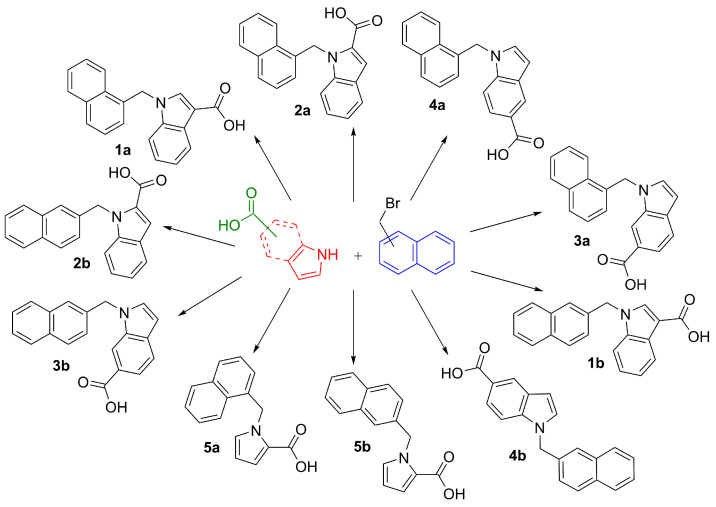
A set of target molecules containing key structural units: a heterocyclic framework (either pyrrole or indole), a lipophilic naphthyl back chain, and a carboxyl group as a polar component responsible for the formation of strong ionic contacts inside the active-site pocket of bCSE.

Inspired by the Nudler group’s results [13], and having carefully examined various antibacterial activities of indole derivatives [22], we selected a chemotype comprising indole as the key structural motif, i.e., a foundation for the development of novel bCSE inhibitors. According to X-ray data, ligands were stabilized by two types of interactions: (1) lipophilic contacts between aliphatic amino acid residues and bulky hydrophobic substituents of indole derivatives such as a bromine atom; (2) hydrogen bonds between guanidine residues of arginine and a carboxyl group of a ligand. It should be noted that around the perimeter of the bCSE active-site pocket, arginine residues are frequent and distributed uniformly. This arrangement allows carboxyl-containing molecules to bind to the holoenzyme efficiently. With the intention to make the synthetic route more straightforward, we chose readily accessible naphthalene derivatives and indole or pyrrole carboxylic acids as sources of the respective hydrophobic and carboxylic components of candidate ligands (Figure 3). All target compounds were prepared by the conventional *N*-alkylation of heterocycles with *α*- or *β*-bromomethylnaphthalene, either in one step starting from indole or pyrrole carboxylic acids, or with an additional saponification step if a corresponding ester was utilized initially.

### 2.2. Enzymatic (bCSE and Human CSE (hCSE)) Assays

The biochemical assays revealed some structure–activity relationships for the obtained compounds in terms of the inhibition of bCSE and hCSE. According to Clustal 2.1 analysis, the percentage similarity between amino acid sequences of the bCSE under study (UniProt ID: A0A2Y2FJW5) and hCSE (UniProt ID: P32929) is 42.1% (Figure S1) [23]. Comparisons of the crystal structure between bCSE (PDB ID: 7md0) and hCSE (PDB ID: 3COG), as well as predictive models using AlphaFold with a high model confidence predicted local distance difference test (pLDDT), indicated a significant resemblance in 3D structure between these proteins (Tertiary structural comparison of bCSE and hCSE.mp4) [24]. Despite significant similarities between hCSE and bCSE, all tested compounds preferentially inhibited the bCSE enzyme. The selectivity can be explained by the metadynamics data showing that the most probable active site is located at a junction between two monomers of tetrameric bCSE, where the diversity of structure is expected to be higher than inside the monomer.

**Figure 4:**
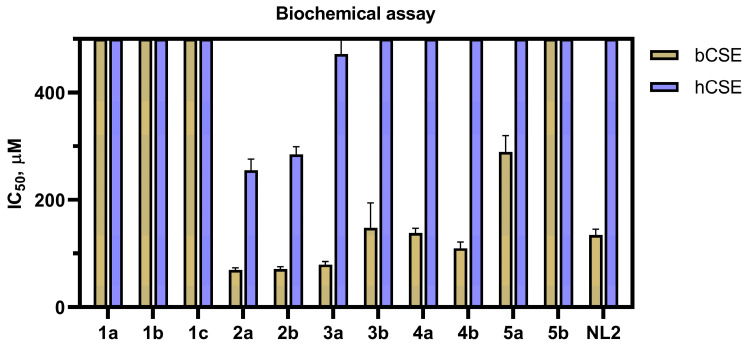
Inhibitory activity of the investigated compounds toward enzymes bCSE and hCSE. The data represent mean ± SD (standard deviation) from at least two independent experiments.

**Figure 5:**
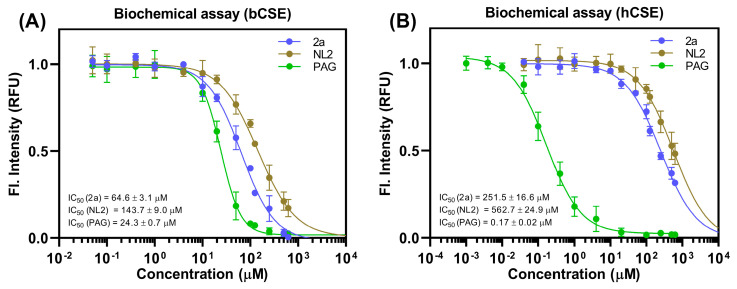
A comparison of in vitro inhibition of bCSE and hCSE by PAG (DL-propargylglycine: an irreversible inhibitor of cystathionine γ-lyase), **NL2**, and lead compound **2a** [25]. Representative curves show concentration-dependent inhibition of H_2_S production by purified bCSE (**A**) and hCSE (**B**). Detection of H_2_S was performed using the WSP5 fluorescent probe. Data represent mean ± SD of at least two independent experiments, each conducted in triplicate.

Compounds **4a** and **4b**, both containing an indole-6-carboxylic acid ring system, manifested almost the same inhibitory activity as **NL2** (5-((6-bromo-1*H*-indol-1-yl)methyl)-2-methylfuran-3-carboxylic acid), against both bCSE and hCSE. Notably, structurally similar compounds, containing a carboxyl group at the same second position on the alkylated heterocyclic ring, showed completely different activity. The presence of a monocyclic aromatic pyrrole system (compounds **5a** and **5b**) led to diminished inhibition, whereas indole-containing compounds (**2a**, **2b**, **3a**, and **3b**) were found to have the best (lowest) half-maximal inhibitory concentrations (IC_50_). Derivatives of indole-3-carboxylic acids (**1a**, **1b**, and **1c**) mostly resembling **NL2** structure did not show noticeable activity toward either enzyme (Figure 4, Table S1). Lead compound **2a** has approximately 3.6-fold selectivity for bCSE over hCSE and a twice as low IC_50_, compared to **NL2** (Figure 5).

It is worth noting that our dose–response curve for PAG toward bCSE has a Hill slope of approximately two. The reason may be a chemical reaction with the enzyme and/or a specific action of PAG as a suicidal bCSE inhibitor [15]. Nevertheless, in the case of hCSE, this value for PAG approaches 1.0, indicating the likely absence of an irreversible reaction under the experimental conditions.

### 2.3. Antibacterial Activity

**Figure 6:**
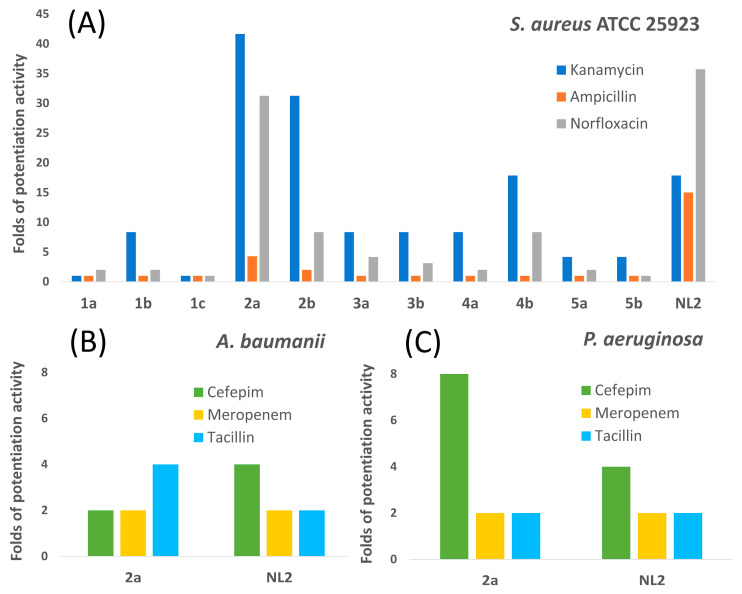
Enhancement of antibiotic activity by the presence of potentiating molecules toward (**A**) *S. aureus* ATCC 25923; (**B**) *A. baumannii* GIMC5509:ABT-52Ts19; and (**C**) *P. aeruginosa* GIMC5016:PA1840/36/2015. The enhancing effect was estimated as MIC of the antibiotic divided by MIC of the antibiotic in the presence of potentiator.

The compounds under investigation were tested for growth inhibition of *S. aureus* ATCC 25923, a strain commonly used in various biological assays, and *S. aureus* INA00761 (methicillin-resistant *S. aureus*; MRSA), which is an antibiotic-resistant strain of clinical importance. In case of *S. aureus* ATCC 25923, all potentiators were tested at a 50 µg/mL concentration due to the absence of their own inhibitory activity toward the bacterial cultures at this concentration. Unexpectedly, *S. aureus* INA00761 demonstrated greater sensitivity to the potentiators, and lower concentrations were used in corresponding microbiological assays: 50 µg/mL for **5a**, **1a**, and **NL2**; 25 µg/mL for **5b** and **3b**; 12.5 µg/mL for **4a**, **2a**, **1b**, **4b**, and **2b**; and 6 µg/mL for **1c** and **3a**. The minimum inhibitory concentrations (MICs) of antibiotics were assessed for each strain as well (Figure 6A, Table S3). The range of concentrations was selected such that the maximum concentration of an antibiotic was 2–4 times higher than its MIC. This approach allowed the evaluation of the potentiating activity of bCSE inhibitors, taking into account their intrinsic antibacterial activity. *S. aureus* INA00761 (MRSA) was resistant to kanamycin and ampicillin; therefore, the 100.0–0.8 µg/mL range of concentrations was employed in this case. Other rows present MICs of antibiotics in combination with potentiators (Table S3).

The presented data suggest that almost all the potentiators increased the effectiveness of antibiotics against *S. aureus* ATCC 25923. By contrast, only **2a** in combination with ampicillin manifested activity against *S. aureus* INA00761 (MRSA) and has the lowest MIC when combined with kanamycin, ampicillin, and norfloxacin against *S. aureus* ATCC 25923. As expected, **NL2** enhanced the effect of kanamycin, ampicillin, and norfloxacin against the MRSA strain. In agreement with the results of the enzymatic assay, pyrrole-containing compounds (**5a** and **5b**) showed a reduced potentiating effect, and compound **1a**, which most closely resembles **NL2** in structure (owing to the presence of a carboxyl group at the third position of the heterocycle), showed almost no activity in combination with antibiotics. It should be pointed out that the same compound is considerably less active when it is applied as a salt (**1c**). Thus, the acid form—as was the case for all the obtained compounds—appears to be the most practical for their biological evaluation (Figure 6A).

Compound **2a** was designated as a “lead compound” for further analyses involving two clinical isolates of Gram-negative bacteria: *A. baumannii* GIMC5509:ABT-52Ts19 and *P. aeruginosa* GIMC5016:PA1840/36/2015 (Figure 6B,C, Table S2). A carbapenem antibiotic (meropenem), a fourth-generation cephalosporin (cefepime), an aminoglycoside (gentamicin), and a broad-spectrum antibiotic of the penicillin group with a β-lactamase inhibitor (piperacillin + tazobactam) were chosen as antibiotics of clinical importance. All antibiotics were tested in the concentration range 512–4 µg/mL. The lead compound vigorously potentiated the activity of cefepime against *P. aeruginosa* GIMC5016:PA1840/36/2015 by decreasing the MIC from 256 to 32 µg/mL. Much weaker potentiating activity was observed in the lead compound’s combination with piperacillin + tazobactam or with meropenem against *P. aeruginosa* isolate GIMC5016:PA1840/36/2015. The microbiological assay revealed that the lead compound possesses a notable potentiating effect in combination with cefepime, meropenem, or piperacillin + tazobactam against *A. baumannii* GIMC5509:ABT-52Ts19. MICs of cefepime in the presence of compound **2a** were decreased from >512 to 256 µg/mL; for piperacillin + tazobactam, from 256 to 64 µg/mL; and for meropenem, from 64 to 32 µg/mL. The addition of bCSE inhibitors did not exert any noticeable effect on the MIC of gentamicin. The potentiating activity of compound **NL2** with almost all tested antibiotics was the same or lower in comparison to **2a** against both clinical multidrug-resistant isolates: *A. baumannii* GIMC5509:ABT-52Ts19 and *P. aeruginosa* GIMC5016:PA1840/36/2015 (Table S4).

### 2.4. ADMET Experiments

**Figure 7:**
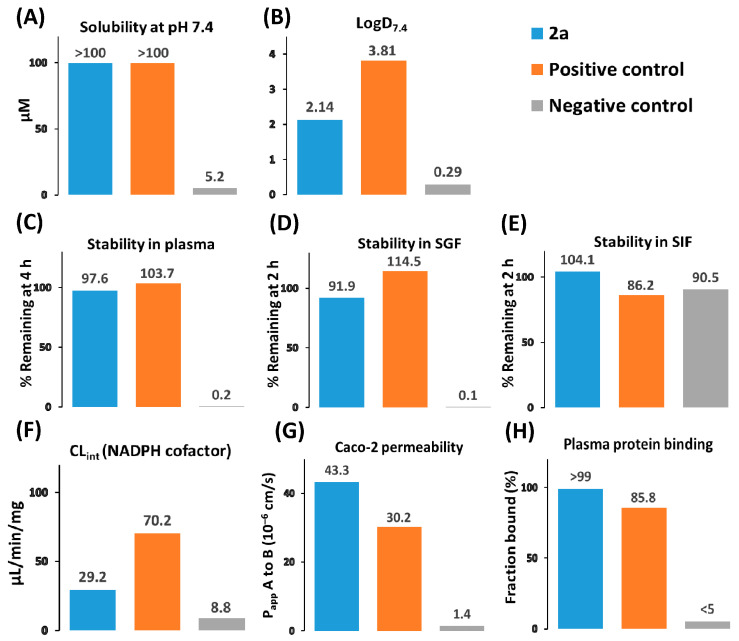
ADME properties of the lead compound. (**A**) Solubility (µM) in 100 mM sodium phosphate buffer pH 7.4. Diclofenac and nicardipine served as positive and negative controls. (**B**) Distribution coefficients between 1-octanol and 100 mM sodium phosphate buffer at pH 7.4. Ketoconazole and paracetamol were used as positive and negative controls. (**C**) Stability in human plasma. The percentage of remaining compounds was determined after incubation for 4 h at 37 °C. Verapamil and propantheline bromide served as positive and negative controls. (**D**) Stability in SGF. The percentage of compounds remaining after incubation for 2 h at 37 °C was determined. Verapamil and omeprazole were chosen as positive and negative controls. (**E**) Stability in SIF. The percentage of remaining compounds was determined after incubation for 2 h at 37 °C. Verapamil and omeprazole were used as positive and negative controls. (**F**) Intrinsic clearance (CL_int_, µL/[min·mg]) in rat liver microsomes. Verapamil and umbelliferone served as positive and negative controls. (**G**) Apparent permeability coefficients for apical to basolateral transport (P_app_ A to B, 10^–6^ cm/s) in a Caco-2 cell monolayer model. Propranolol and atenolol were used as positive and negative controls. (**H**) The fraction bound (f_b_, %) by human plasma in a rapid equilibrium dialysis (RED) assay. See the supporting information for the detailed description of assay protocols and results.

These experiments were conducted to estimate the solubility, stability, permeability, lipophilicity, plasma protein binding, and metabolism by rat liver microsomes (Figure 7). The lead compound was soluble (>100 µM) in phosphate buffer at neutral pH and was found to have good plasma, SGF (simulated gastric fluid), and SIF (simulated intestinal fluid) stability (Tables S5–S7). The logD_7.4_ value was in the range of 1–3, suggesting that the lead compound has moderate lipophilicity at pH 7.4 [26]. The measured logD_7.4_ value was in good agreement with the predicted value (Table S8). A Caco-2 cell permeability assay showed that the lead compound had a high penetration ability, and the efflux ratio was less than 2.0 (Table S9). Compound **2a** showed moderate intrinsic clearance (29.2 µL/[min·mg]) in rat liver microsomes with NADPH as a cofactor, as well as low intrinsic clearance (9.2 µL/[min·mg]) in rat liver microsomes with UDPGA (uridine 5′-diphospho-*α*-D-glucuronic acid) as a cofactor (Table S10) [27]. Many acidic drugs are capable of strong protein binding (>99%) and tend to associate predominantly with albumin [28,29]. The lead compound contains a carboxyl group and is strongly bound by human plasma (Table S11). We are planning to conduct pharmacokinetic studies to understand whether the lead compound has unusual pharmacokinetic properties due to the strong plasma protein binding.

**Figure 8:**
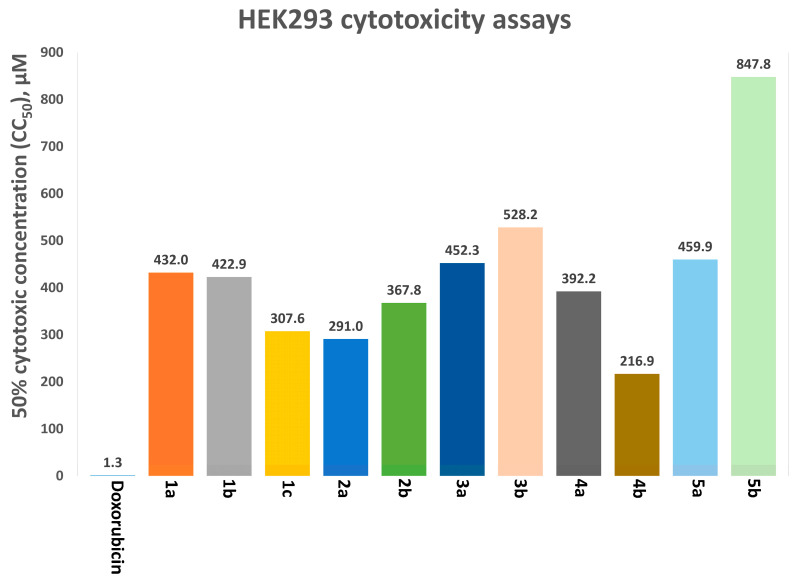
HEK293 cytotoxicity assays, to determine half-maximal cytotoxic concentration (CC50) after incubation for 72 h in a humidified atmosphere containing 5% of CO_2_ at 37 °C. Doxorubicin served as a control with high toxicity.

An analysis of cytotoxicity indicated that the half-maximal cytotoxic concentrations (CC_50_) on HEK293 cells (Figure 8, Table S12) were 2–6 times higher than the IC_50_ values for tested inhibitors. The lead compound possessed a satisfactory in vitro therapeutic index.

### 2.5. Molecular Dynamics Simulations

Cystathionine γ-lyase is present as a homotetramer in the Protein Data Bank (PDB); therefore, we wondered how important the formation of the tetramer is to the structure of the **NL2**-binding site. Equilibrium molecular dynamics simulations were carried out for the monomer; in the case of the monomer and dimer, significant fluctuations of the N-terminus position of the protein were observed. Messerschmidt and coworkers reported that bCSE is a tetramer in its native form. This important detail led us to the hypothesis about multiple protein–ligand interactions in multimeric bCSE [30]. In the case of the homotetramer, no such oscillations were detectable. It is important to note that in the tetramer structure, the N-terminus of the protein is involved in the formation of the **NL2**-binding site in the adjacent tetramer subunit. Obviously, the use of a tetramer in the modeling of cystathionine γ-lyase complexes is mandatory according to both structural and functional data [13,31].

As a reference analysis and to validate the method, we simulated the binding of **NL2** to the enzyme by the funnel metadynamics method [32,33] with a GROMACS/PLUMED packages combination [34,35]; the complex of this enzyme with **NL2** has been described well. As a result, two minima were found on the potential energy surface, and the deepest minimum corresponded to the position of **NL2** according to X-ray data, although in the starting state of the system, **NL2** was in a water environment. The second significant minimum on the potential energy surface was slightly shifted, and the shift was possibly caused by a dynamic change in the binding site. After a comparison of energies of the positions of the ligand in the aqueous environment and in the active site, the binding energy can be estimated as 7.5 kcal/mol, and with a correction for a limited volume: 1.59 kcal/mol. The experimental inhibition constant was 1.8 μM; if we assume that the inhibition directly depends on the inhibitor’s binding constant, then the binding constant can be estimated at 7.8 kcal/mol. In the course of **NL2** binding modeling, we observed 11 events of complex formation and dissociation. The convergence of the binding energy was observed already after five events of complex formation. It can be concluded that for the selected system with the protein tetramer, more than five binding events should be examined to obtain converging results.

**Figure 9:**
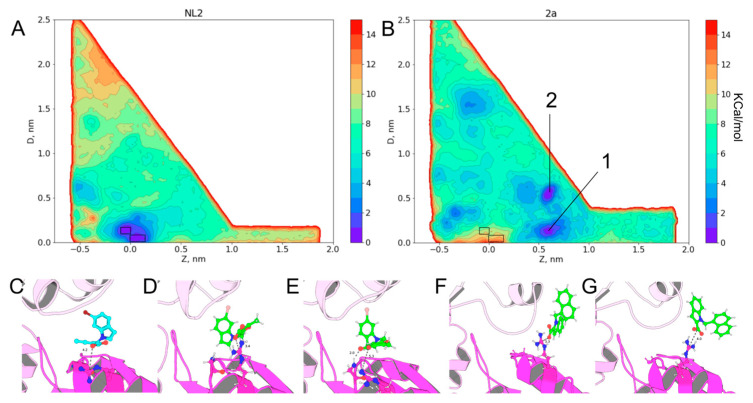
Results of funnel metadynamics simulation for **NL2** and **2a** compounds. (**A**) Surface of the free energy of binding of **NL2** to bCSE; the zones with minimal energy are marked by rectangles. (**B**) Surface of the potential energy of binding **2a** to bCSE; rectangles mark the zones with binding of **NL2**; numbers 1 and 2 mark the deepest minimums for **2a** binding. (**C**) **NL2** binding site in X-RAY structure; Arg104 is presented as ball and sticks where carbons are colored magenta. (**D**,**E**) The variants of binding of **NL2** according to metadynamics simulations; Arg104 and Gly100 are presented as ball and sticks where carbons are colored magenta. (**F**) **2a** binding with bCSE according to metadynamics simulations in energy minimum one; Arg104 is presented as ball and sticks where carbons are colored magenta. (**G**) **2a** binding with bCSE according to metadynamics simulations in energy minimum two; Arg104 is presented as ball and sticks where carbons are colored magenta.

The validation of the method allowed us to move on to the assessment of new potential inhibitors **1–5**. All compounds have significant minima on FES (free energy surface) outside **NL2** position coordinates, indicating the possibility of the substances’ binding to the protein without affecting its function (Figures S2 and S3). Compounds **1c**, **2a**, and **3a** stood out from the standpoint of binding efficiency. A feature of the binding of these compounds is the presence of a limited number of minima comparable in energy to **NL2**, but the position of these substances differs from that of **NL2**. This modeling did not allow for the determination of a direct relation between the binding energy of a substance and the inhibitory effect of the enzyme. To solve this riddle, it can be assumed that the closer the binding site to the putative **NL2**-binding pathway, the more likely are such positions on the potential energy surface to have an inhibitory effect. Compound **2a** has two pronounced minima near the axis along which the putative **NL2** binding occurs. Considering the presence of activity in this compound, we believe that one of these minima falls into a functionally significant site (Figure 9).

### 2.6. Molecular Docking

**Figure 10:**
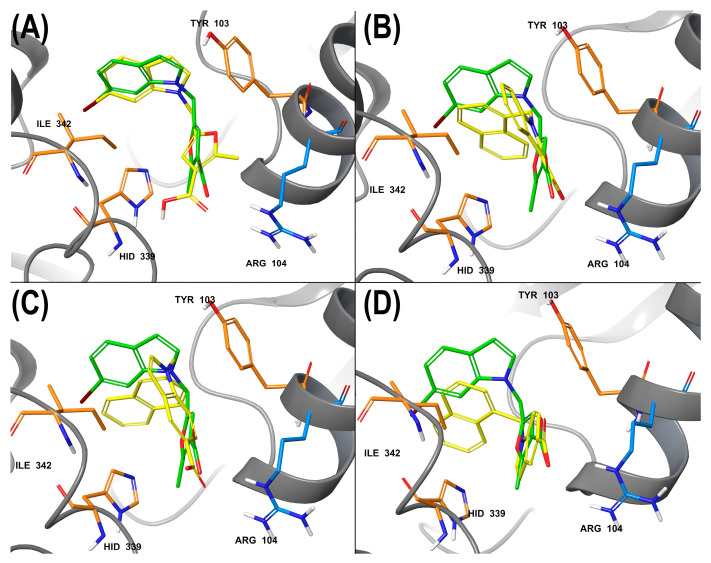
(**A**) Interaction of bCSE and redocked **NL2** used as a control. Green: **NL2**, a crystal pose in PDB model 7MCU, yellow: the best docking pose; (**B**) the binding pose of **1c** (poor activity), (**C**) **4b** (active), (**D**) **4a** (moderately active); orange and blue: key amino acid residues. In all cases (panels **B**–**D**), orientation relative to key amino acids was preserved. The patterns of intermolecular interactions coincided: the aromatic part retains lipophilic contacts with Ile342, and the polar carboxyl group retains contacts with Arg104.

The first stage of calculations involved the docking of the investigated structures into the bCSE model in combination with an **NL2** molecule. **NL2** was used as a control structure. All docked compounds reproduced the pharmacophore properties of **NL2** described in the 7MCU model (Figure 10). The obtained results are summarized in Table S13.

The interactions of the control substance were accurately replicated by the binding positions of the compounds under study. Nonetheless, it is worth noting that scoring function values did not correlate with the levels of enzymatic and antibacterial activities. For instance, the inactive compounds **1a**, **1b**, and **1c** had better parameters compared to lead compound **2a** and outperformed moderately active structures. The preferred poses of ligands in the active site did not look relevant: the binding poses of all structures were largely similar and partially reproduced the pharmacophore characteristics of the control structure.

**Figure 11:**
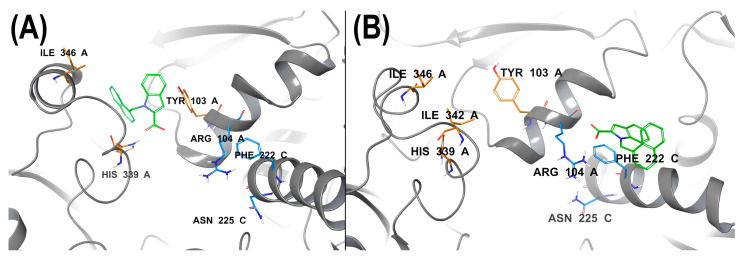
Two possible binding sites for a ligand. (**A**) The original binding site, (**B**) a novel binding site described by us, which is located between monomers.

According to metadynamics, the investigated ligands are able to bind to tetrameric bCSE in two ways within the same active-site pocket. The binding may be either an interaction with the protein at the canonical site involving Ile346/342, Tyr103, His339, and Arg104, as described in the work of Nudler et al. [13], or a binding in the region of residues Phe222, Asn225, and Arg104 (Figure 11). In the former case, as in the PDB model (7MCU and others), all interactions take place within the monomer (Figure 11A), whereas the latter scenario is implemented at a junction of two monomers (Figure 11B, chains A/C).

Accordingly, on the basis of the results obtained after detailed analysis of molecular metadynamics data, docking of the ligand was performed again, to the tetramer structure. The results are summarized in Table S14.

**Figure 12:**
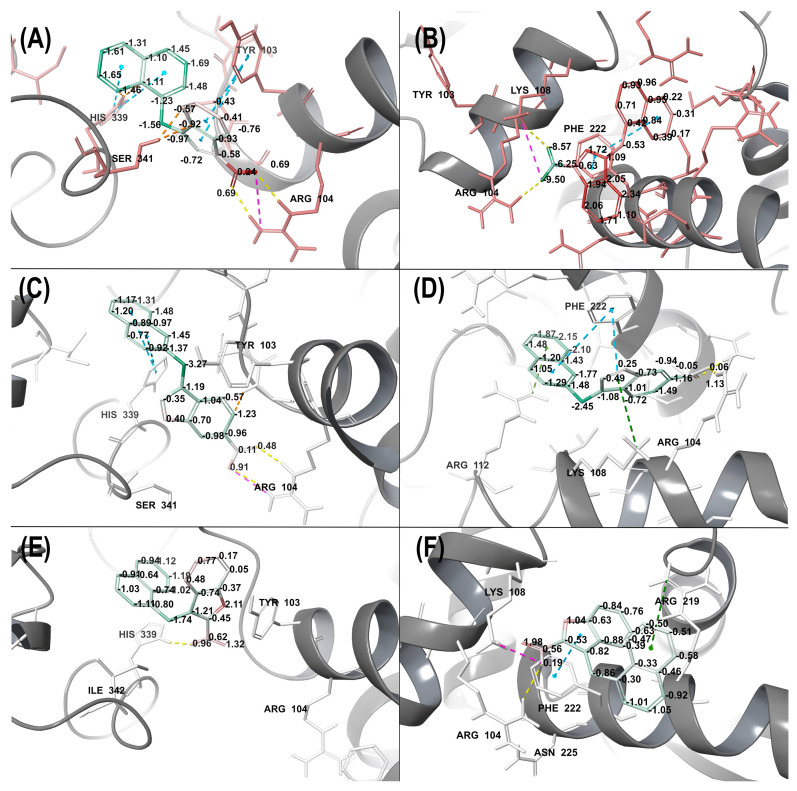
Free energy distribution and strained contacts within bCSE structure. The strain energy level is reflected by gradient coloring: from red (maximal strain energy) to green (energetically favorable contacts), in the range −4 to +4 kcal/mol. Colors of the dashed lines indicate: blue, π-stacking; green, π-cation interaction; yellow, a hydrogen bond; violet, a salt bridge; and orange, a strained contact. (**A**)—compound **1c** in site 1, (**B**)—**1c** in site 2, (**C**)—**4b** in site 1, (**D**)—**4b** in site 2, (**E**)—**2a** in site 1, (**F**)—**2a** in site 2.

To determine the factors weakening the affinity of the studied molecules and for the purpose of their ranking, Gibbs free energy (ΔG) parameters were calculated by the MM–GBSA (molecular mechanics/generalized Born surface area) method, which takes into consideration the effects of solvation of a ligand–protein complex. As a result, per-atom energy distribution and defined strained contacts were obtained, indicating unfavorable ligand–substrate interactions (Figure 12).

In the case of the inactive compound **1c**, the analysis of free energy distribution and of the extent of internal strains indicated the presence of energetically unfavorable interactions: (1) with Ser341 and His339 at the original site (Figure 12A) and (2) strained contacts of the indole ring at the junction of two monomers (Figure 12B).

Compound **4b**, highly active in the enzymatic assay, in contrast to **1c** was found to form a network of energetically favorable ligand–protein contacts. Strained contacts are minimal in the case of the original binding site (Figure 12C). Notably, **4b** comes into even more favorable contacts at the discovered active site. A possible reason is the energetically favorable embrace of the ligand by a lipophilic ledge, formed by the duet of Phe222 and Ile223. In addition, stacking interactions with aromatic parts of the ligand and Phe222 are seen in the complex. Hydrogen bonds with Arg104 and π-cationic bonds with Arg112 and Lys108 favorably complement the complex, making it stabler (Figure 12D).

Compound **2a** binds equally to the two sites of interest and shows interactions that are equivalent in terms of energies ΔG, with the overall absence of any significant strained contacts (Figure 12E,F). In the case of binding within the novel active site, the molecule forms the same lipophilic contacts as **4b** does, engages in a π-cationic interaction with Arg219, and forms a salt bridge with Lys108 through a carboxyl group (Figure 12F).

The GlideScore scoring function does not take into consideration ligand-induced rearrangements in the active-site pocket. Nevertheless, these effects are taken into account, albeit in a limited manner, in MM–GBSA. This method complements the calculation with the environment of the ligand at a distance of 5 Å as well as with an implicit solvent. As a result, ΔG was calculated for each complex (Table S15). With these free energy values in hand, an energy advantage of the ligand–protein complexes can be parameterized.

Thus, in most cases (except for **4a** and **4b**), the ligands preferentially interact with the bCSE enzyme at the original binding site. Nevertheless, due to a small difference in Gibbs free energy, the active site described herein can also be considered favorable for the interaction. These two active sites even overlap: both include interactions with Arg104. Therefore, the discovery of this novel active site creates an opportunity for the development of new bCSE inhibitors.

## 3. Materials and Methods

### 3.1. General Procedures, Materials, and Equipment

#### 3.1.1. Glassware and Reaction Techniques

Unless stated otherwise, all glassware was flame-dried or oven-dried, cooled in vacuum, and back-filled with nitrogen or argon; then the reactions were carried out in that inert atmosphere, and additions of solids were performed under positive pressure of that inert atmosphere. Unless stated otherwise, all yields are isolated yields. Room temperature was generally in the range 21–25 °C. Reaction temperature refers to the temperature of the oil bath.

#### 3.1.2. Solvents, Reagents, and Chemicals

Extra dry dichloromethane (DCM) and dimethylformamide (DMF) were purchased from commercial suppliers. Dry tetrahydrofuran (THF) and diethyl ether were obtained according to standard procedures. The rest of solvents were reagent grade; all reagents and chemicals come from commercial sources and were used without further purification. All water for solutions and assays was deionized water.

#### 3.1.3. Column Chromatography and Thin Layer Chromatography (TLC)

All chromatographic purification procedures were flash (ca. 2–3 atm. of pressurized air) column chromatography (FCC) on silica gel (Silicycle 60, 0.04–0.063 mm). Supelco aluminum-backed TLC plates (silica gel, 200 µm, 60 Å, F-254 analytical plates) were utilized for TLC. The development of staining was undertaken first by nondestructive visualization under a UV lamp (254 nm); destructive development was performed on staining solutions of potassium permanganate (KMnO_4_), cerium molybdate (CAM), or phosphomolybdic acid (H_3_PMo_12_O_40_), followed by heating.

#### 3.1.4. NMR Spectroscopy (Engelhardt Institute of Molecular Biology RAS, Moscow, Russia)

The NMR experiments were performed on a Bruker Avance-300 spectrometer, operating at a resonance frequency of 300.18 MHz for ^1^H nuclei and at 75.48 MHz for ^13^C nuclei. Chemical shifts are reported in units of δ (ppm) using the internal standard residual CDCl_3_ (δ = 7.26 ppm for ^1^H NMR spectra and δ = 77.16 ppm for ^13^C NMR spectra) or DMSO-*d_6_* (δ = 2.50 ppm for ^1^H NMR spectra and δ = 39.52 ppm for ^13^C NMR spectra). The following abbreviations are used to present multiplicities: s = singlet, br. s = broad singlet, d = doublet, t = triplet, q = quartet, p = pentet, m = multiplet, app = apparent. Analytes and common impurities were identified by means of common standards [36,37].

#### 3.1.5. Mass Spectrometry (MS) (Sirius University of Science and Technology, Sochi, Russia)

High-resolution MS (HRMS) analyses were performed using a Bruker maXis II 4G ETD mass spectrometer and an UltiMate 3000 chromatograph equipped with an Acclaim RSLC 120 C18 2.2 μm 2.1 × 100 mm column. The spectrum registration mode was electrospray ionization (ESI), with a full scan between *m*/*z* 100 and 1500, tandem MS (MS/MS) with a selection of the three most intense ions, collision-induced dissociation (CID) at 10–40 eV, and nitrogen as a collision gas.

#### 3.1.6. Purity Analysis (Sirius University of Science and Technology, Sochi, Russia)

All compounds submitted for biological and ADMET testing were determined to be more than 95% pure. The purity was measured using a Vanquish Flex series UHPLC with the diode array detector DAD-FG (Thermo Fisher Scientific, Germering, Germany). Analytical separation was carried out at 40 °C on Poroshell 120 EC-C18 column (100 mm × 2.1 mm, particle size 1.9 µm, Agilent, Santa Clara, CA, USA) using a flow rate of 0.50 mL/min with detection at 254 nm. The mobile phases were 0.1% *v*/*v* formic acid in water (phase A) and 0.1% *v*/*v* formic acid in acetonitrile (phase B). A linear gradient was applied starting at 90% A, increasing to 90% B within 2.5 min, and keeping 90% B for 1 min. Subsequently, the column was re-equilibrated for 2.5 min at 90% A.

### 3.2. Synthesis of Target Compounds

#### 3.2.1. General Procedure 1: N-alkylation of Indole Carboxylic Acids to Obtain **1a**, **1b**, **1c**, **3a**, **4a**, and **4b**

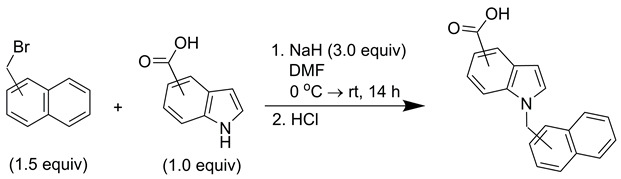



A dry round-bottom flask was charged with a relevant nitrogen-containing heterocyclic compound (1 equiv.) in an inert atmosphere. The compound was dissolved in extra-dry DMF. Sodium hydride (3 equiv., a 60% suspension in mineral oil) was introduced at 0 °C under N_2_. The resulting solution was continuously stirred at this temperature for 30 min. Then, 1-(bromomethyl)naphthalene or 2-(bromomethyl)naphthalene (1.5 equiv.) was added to the mixture at 0 °C. The reaction mixture was stirred for 1 h at this temperature, and the solution was allowed to warm up to room temperature and was stirred until the completion of the reaction (circa 14 h). The sodium salt of indole carboxylic acid precipitates abundantly during extraction, thereby interfering with effective phase separation. The problem was solved by the addition of a concentrated NaCl solution. Nevertheless, turbidity persisted until DCM was present in the aqueous phase. The aqueous phase was boiled to remove the DCM residue, and a transparent solution was obtained. After cooling to room temperature, the aqueous phase was acidified with HCl to pH 3–4, and a lot of brown precipitate fell out. The sediment was filtered out, washed four times with 5 mL of distilled water, and dried under reduced pressure overnight to obtain the final compound.

#### 3.2.2. General Procedure 2: N-alkylation of Pyrrole and Indole Carboxylates to Obtain **2c**, **2d**, **3c**, **5c**, and **5d**

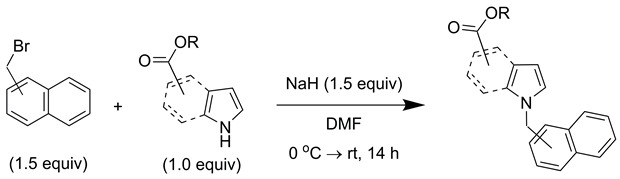



A dry round-bottom flask was charged with a relevant nitrogen-containing heterocyclic compound (1 equiv.) in an inert atmosphere. The compound was dissolved in extra-dry DMF. Sodium hydride (1.5 equiv., a 60% suspension in mineral oil) was added at 0 °C under N_2_. The resulting solution was continuously stirred at this temperature for 30 min. Next, 1-(bromomethyl)naphthalene or 2-(bromomethyl)naphthalene (1.5 equiv.) was introduced at 0 °C. The reaction mixture was stirred for 1 h at this temperature, and the solution was allowed to warm up to room temperature and was stirred until the completion of the reaction (circa 14 h). After that, the reaction mixture was diluted with 2.5% HCl (30 mL), and the aqueous layer was extracted with ethyl acetate (3 × 15 mL). The combined organic layers were washed with water, a saturated NaHCO_3_ solution, and brine, and dried with Na_2_SO_4_. The combined organic layers were washed with sat. aq. NaCl (saturated aqueous solution), dried over Na_2_SO_4_, filtered, and concentrated in vacuo.

#### 3.2.3. General Procedure 3: Saponification of N-alkylated Pyrrole and Indole Carboxylates to Obtain Target Compounds **2a**, **2b**, **3b**, **5a**, and **5b**

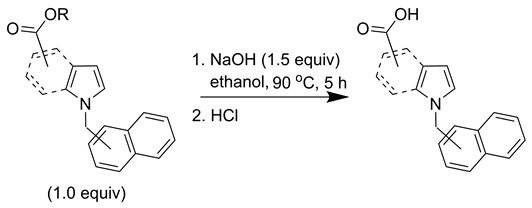



Into a round-bottom flask charged with the corresponding carboxylate (1 equiv.) ethanol, a 1.5 M solution of NaOH in water (1.5 equiv.) was added. The resulting solution was stirred and refluxed in an oil bath (90 °C) for 5 h. The mixture was cooled to room temperature and evaporated in vacuo. The obtained residue was dissolved in 20 mL of water and extracted with DCM (3 × 15 mL). The aqueous layer was acidified with HCl to pH 3–4, leading to the formation of an abundant white precipitate. It was filtered off, washed four times with 5 mL of distilled water, and dried under reduced pressure overnight to obtain the final compound.

### 3.3. IC_50_ Assay

Fluorescence spectrophotometry-based inhibitory activity toward purified bCSE and hCSE was measured in a 96-well nonbinding black plate (Greiner, Kremsmunster, Austria) at 100 μL of the reaction mixture per well. bCSE and hCSE (0.02 μM) were preincubated in a thermoshaker (Biosan, Riga, Latvia) (30 °C, 300 rpm) for 40 min in 50 mM sodium phosphate buffer (pH 8.0), supplemented with 10 μM pyridoxal 5′-phosphate (Sigma-Aldrich, St. Louis, MO, USA), 0.1% of Tween 20 (Sigma-Aldrich, St. Louis, MO, USA), and 1.2 μM Washington State Probe-5 (Cayman Chemical, Ann Arbor, MI, USA) (excitation: 500 nm, emission: 530 nm) at different concentrations of a tested inhibitor. The reaction was initiated by the addition of 100 μM L-cysteine (TCI, Chennai, Tamil Nadu, India) in 50 mM sodium phosphate buffer pH 8.0 to the reaction mixture, and the enzymatic activity was monitored for 12 h using a CLARIOstar Plus instrument (BMG LABTECH GmbH, Ortenberg, Germany). The 100 µM L-cysteine concentration used in the experiments corresponds to the maximal reported intracellular concentration in bacteria [13]. The data were normalized to the positive and negative controls, and IC_50_ values were obtained by fitting to a four-parameter sigmoidal curve (inhibitor vs. response, variable slope model) in GraphPad Prism 8 software (GraphPad by Dotmatics, Boston, MA, USA). Each experiment was conducted with 2–3 technical replicates. IC_50_ values were means of at least two independent experiments.

### 3.4. Microbiological Assays

#### 3.4.1. Tested Bacterial Strains

At the first stage of evaluation of the obtained compounds, we used two strains of Gram-positive bacteria: *S. aureus* ATCC 25923 and *S. aureus* INA00761 obtained from collections. *S. aureus* INA00761 (MRSA) carries genes of resistance to methicillin-like antibiotics. For further tests of selected lead compound, we employed two clinical isolates of Gram-negative bacteria: *A. baumannii* GIMC5509:ABT-52Ts19, obtained from expectoration in Moscow City Clinical Hospital No. 52, and *P. aeruginosa* GIMC5016:PA1840/36/2015, obtained from discharge from a wound in Moscow City Clinical Hospital No. 36. Both bacteria belong to the ESKAPE group of highly virulent and antibiotic-resistant pathogens.

#### 3.4.2. Assessment of MICs

Overnight (ON) culture of a specific strain was grown in the liquid Luria–Bertani nutrient medium (LB broth, BD Difco) for 18 h at 37 °C, with agitation at 180 rpm using an Innova 44R orbital shaker (Eppendorf, Hamburg, Germany). After a certain period, 50 µL of the ON culture was transferred into 5 mL of the LB medium and grown for 3 h at 37 °C with agitation. Growth in the liquid medium was assessed by means of turbidity of the suspension, measured using a Densi-La-Meter II densitometer (Erba Lachema s.r.o., Brno, Czech Republic). Then, the bacterial suspension was diluted to the concentration 10^6^ cells/mL with sterile LB and used as a seed culture [38,39].

For the assessment of MICs, 96-well microtiter plates were utilized at 200 µL of a medium per well. Each well was filled with 100 µL of the seed culture. After that, 10 µL of a solution of each tested synthetic compound (potentiators) was added into each experimental well. Finally, 90 µL of twofold serial dilutions of an antibiotic in a sterile LB was added into individual wells. The final concentration of tested compounds (potentiators) was 50 µg/mL for all strains except *S. aureus* INA00761, for which concentrations were lower for the reasons discussed in the “Results and Discussion” section.

As a positive control, we set up wells with 100 µL of the bacterial culture and 100 µL of the sterile LB medium. **NL2** was chosen as a reference compound showing potentiating activity, according to published data [13]. Negative-control wells contained 200 µL of the sterile medium. The plates were incubated in a thermostat for 18–20 h at 37 °C. The bacterial growth was assessed as optical density at 600 nm on a CLARIOstar microplate reader (BMG LABTECH GmbH, Ortenberg, Germany)).

### 3.5. ADMET Experiments

#### 3.5.1. Caco-2 Cell Penetration

Caco-2 cells were seeded onto inserts of a PET (polyethylene terephthalate) membrane in a 24-well plate (surface area: 0.33 cm^2^, Corning, cat. # 353495) at a cell density of 0.5 × 10^5^ cells/insert. The culture medium consisted of high-glucose DMEM containing 10% of fetal bovine serum, 4 mM L-glutamine, 1% of a solution of nonessential amino acids (PanEco, Moscow, Russia), 100 U/mL penicillin, and 100 μg/mL streptomycin. The cells were maintained for 21 days at 37 °C and 5% CO_2_. The medium was refreshed every 3 days. A Lucifer yellow rejection assay was applied to determine cell monolayer integrity. Wells with Lucifer yellow rejection values below 97% were not used for assays. The Lucifer yellow rejection value was calculated using the equation: LY % rejection = 100*(1−FI_b_/FI_a_), where FI_b_ is the fluorescence in the basolateral compartment and FI_a_ is the fluorescence in the apical compartment. Solutions of test and reference compounds (propranolol and atenolol) were prepared by diluting 10 mM DMSO stocks in HBSS buffer containing 0.5% of BSA (final concentration 10 μM). Both apical-to-basolateral (AB) and basolateral-to-apical (BA) transport were assessed. For apical-to-basolateral transport, 0.3 mL of a test compound solution was added to the apical side, and 0.8 mL of HBSS with 0.5% of BSA was added to the basolateral side. For basolateral-to-apical transport, 0.8 mL of a test compound solution was added to the basolateral side and 0.3 mL of HBSS with 0.5% of BSA was added to the apical side. The Caco-2 cell monolayers were then incubated for 2 h at 37 °C. The pH values of the apical and basolateral compartments were 7.4. Next, 100 μL aliquots were taken from both the apical and basolateral compartments, 300 µL of acetonitrile containing an internal standard was introduced, and the mixture was centrifuged for 10 min at 10,500× *g*, 4 °C. The supernatant was transferred to a vial and analyzed by liquid chromatography coupled with tandem MS (LC–MS/MS). The AB (or BA) apparent permeability coefficients (P_app_, 10^–6^ cm/s) were calculated according to the following formula [40,41]: P_app_ = (V × ∆C)/(∆t × A × C_0_), where V is the volume of the receiver compartment (cm^3^), C_0_ is the initial drug concentration in the donor compartment (μM), ∆C is the concentration in the receiver compartment (μM), ∆t is the incubation time (s), and A is the surface area of the cell monolayer (cm^2^). An efflux ratio was calculated by dividing P_app_ (BA) by P_app_ (AB). All compounds were tested in duplicate.

#### 3.5.2. Stability in SGF, SIF, and Human Plasma

SIF was prepared by adding pancreatin to SIF without an enzyme (Ricca, cat. # 7109.75-16) to a final concentration of 10 mg/mL. SGF was prepared by adding pepsin to SGF without an enzyme (Ricca, cat. # 7108-16) to a final concentration of 1.6 mg/mL. A test compound (final concentration 10 μM) was incubated at 37 °C in SGF, SIF, or human plasma for 2, 2, or 4 h, respectively. 100 μL aliquots were taken from each sample and 300 μL of acetonitrile containing an internal standard was added. The mixture was centrifuged for 10 min at 10,500× *g* and 4 °C and the supernatant was analyzed by LC–MS/MS. The controls were verapamil, omeprazole, and propantheline bromide. All compounds were tested in triplicate. The percentage of a remaining compound after incubation was determined based on the chromatographic peak area of the sample after incubation and that of time zero (freshly prepared and injected) sample.

#### 3.5.3. Plasma Protein Binding

Plasma protein binding was assessed using RED device inserts with built-in cellulose acetate dialysis membranes (molecular weight cutoff: 8 kDa). The final concentration of the test compound in human plasma was 5 μM. The samples were prepared by placing 300 μL of human plasma into the plasma compartment of the RED device and 500 μL of phosphate-buffered saline (pH 7.4) into the buffer compartment. The RED device was fixed to a shaker, placed in a CO_2_ (5%) incubator, and incubated for 6 h at 37 °C. After an equilibration step, 100 μL aliquots were taken from each compartment; 100 μL of plasma was added to the buffer compartment aliquot and 100 μL of buffer was added to the plasma compartment aliquot. After mixing, 600 μL of acetonitrile containing an internal standard was added. The mixture was centrifuged for 10 min at 10,500× *g* and 4 °C, and the supernatant was analyzed by LC–MS/MS. The controls were propranolol and atenolol. All compounds were tested in duplicate. Fraction bound f_b_ was determined via the following equation: f_b_ (%) = [1 − (C_b_/C_p_)] × 100, where C_b_ is the concentration of an analyte in the buffer compartment (μM), and C_p_ is the concentration of the analyte in the plasma compartment (μM).

#### 3.5.4. Microsomal Stability Assay

The metabolic stability of a test compound was evaluated based on the rate of disappearance during incubation with rat liver microsomes. A test compound (1 µM) was incubated with rat liver microsomes (0.5 mg protein/mL) at 37 °C in 100 mM sodium phosphate buffer (pH 7.4) in the presence of NADPH (phase I cofactor) or UDPGA (phase II cofactor), with an incubation volume of 600 µL. Duplicate 50 μL aliquots were removed immediately after compound addition (time zero) and at the assay timepoints of 5, 15, 30, and 45 min. The reaction was quenched by the addition of 100 µL of acetonitrile containing an internal standard, followed by centrifugation at 10,500× *g* for 10 min. The supernatant was analyzed by LC–MS/MS. A compound’s metabolic stability was determined by calculating its intrinsic clearance and in vitro half-life values. The natural logarithm of a peak area ratio (compound peak area/internal standard peak area) was plotted against incubation time, and the slope of the line was determined. Half-life (t_1/2_) was determined using the following equation: t_1/2_ (min) = 0.693/slope. Intrinsic clearance was determined via the following equation: CL_int_ (μL/[min·mg]) = (0.693/t_1/2_) × (μL incubation volume/mg microsomal protein). The controls were verapamil and umbelliferone.

#### 3.5.5. Solubility

A stock solution of a compound (10 mM in DMSO, 10 μL) was added to 990 μL of 100 mM sodium phosphate buffer (pH 7.4) and incubated at 25 °C for 2 h with constant shaking. The suspension was filtered through the PVDF membrane filter (Merck Millipore, Burlington, MA, USA). To 320 µL of the filtrate, 80 µL of acetonitrile was added and mixed. The mixture was transferred to a vial and analyzed by ultra-high-performance liquid chromatography (UHPLC) with UV detection. Concentrations were calculated from a 5-point calibration curve. The calibration range was 1 to 100 μM. The controls were diclofenac and nicardipine. All compounds were tested in triplicate.

#### 3.5.6. LogD

Coefficients of distribution between 1-octanol and 100 mM sodium phosphate buffer (pH 7.4) were determined by the miniaturized shake flask method. Namely, 5 μL of a 10 mM compound’s solution in DMSO was dissolved in 245 μL of 1-octanol saturated with phosphate buffer. Next, 500 μL of phosphate buffer saturated with 1-octanol was added, followed by mixing for 3 h. The phases were separated by centrifugation (10,500× *g*, 5 min), and the samples were allowed to stand at room temperature for 3 h to ensure the complete separation of the two phases. After that, 50 μL of the octanol phase (top layer) was aspirated and diluted with 500 μL of 1-octanol saturated with phosphate buffer. Then, the remaining octanol phase was removed; 50 μL of the buffer layer was aspirated and diluted with 500 μL of phosphate buffer saturated with 1-octanol. Both the octanol and phosphate buffer samples were analyzed by LC–MS/MS. The distribution coefficient was determined according to the following equation: logD_7.4_ = log_10_ (peak area for octanol sample/peak area for buffer sample). The controls were paracetamol and ketoconazole. All compounds were tested in triplicate.

ACD/Labs Percepta Platform software (ACD/Labs Release 2021.2.2, Advanced Chemistry Development, Inc., Toronto, ON, Canada, https://www.acdlabs.com, (accessed on 18 September 2023)) was employed for predicting logD values.

#### 3.5.7. LC–MS/MS Analysis

The LC–MS/MS system consisted of an Ultimate 3000 RS UHPLC instrument (Thermo Scientific, Germering, Germany) coupled to a triple quadruple mass spectrometer (EVOQ Elite, Bruker, Bremen, Germany). Chromatographic separation was achieved by reversed-phase chromatography and gradient elution. Separation of the analytes was carried out on a Poroshell 120 EC-C18 column (100 mm × 2.1 mm, particle size 1.9 μm, Agilent) maintained at 40 °C. The injection volume was 1 μL, and total chromatographic run time was 9.0 min. The mobile phases were 0.1% *v*/*v* formic acid in water (phase A) and 0.1% *v*/*v* formic acid in acetonitrile (phase B). A linear gradient was implemented at a flow rate of 400 μL/min starting at 95% A, increasing to 95% B within 3.5 min and keeping 95% B for 1.5 min. After that, the column was re-equilibrated for 4 min at 95% A. The detection of target compounds was conducted in multiple reaction monitoring (MRM) mode on a triple quadruple mass spectrometer equipped with a heated ESI source. Verapamil, omeprazole, propantheline, propranolol, atenolol, umbelliferone, paracetamol, and ketoconazole were analyzed in positive ionization mode and the monitored transitions were *m/z* 455 > 165 for verapamil, *m/z* 346 > 198 for omeprazole, *m/z* 368 > 326 for propantheline, *m/z* 260 > 116 for propranolol, *m/z* 267 > 145 for atenolol, *m/z* 163 > 107 for umbelliferone, *m/z* 152 > 110 for paracetamol, and *m/z* 531 > 489 for ketoconazole. The lead compound was analyzed in negative ionization mode, with monitoring of the *m/z* transition 300 > 127. The source capillary voltage was 4000 and 3500 V in positive ion and negative ion modes, respectively. The cone temperature was set to 350 °C, and the cone gas to 20 psi. The heated probe temperature was set to 250 °C, and the probe gas flow to 40 psi. The nebulizing gas was set to 60 psi, and the collision gas (argon) to 1.5 mTorr. Bruker MS Workstation software (version 8.2.1; Bruker, Bremen, Germany) was used for data acquisition and processing.

#### 3.5.8. Cytotoxicity Assay

The HEK 293 cell line was obtained from the European Collection of Authenticated Cell Cultures (ECACC, cat. # 85120602, Salisbury, UK). The cells were grown in a monolayer culture in Dulbecco’s Modified Eagle’s Medium (DMEM) (PanEco, Moscow, Russia), supplemented with 2 mM L-glutamine, 1% of a penicillin/streptomycin solution (Capricorn Scientific, Ebsdorfergrund, Germany), and 10% of heat inactivated fetal bovine serum (HyClone, Cytiva, Marlborough, MA, USA) in a humidified atmosphere containing 5% of CO_2_ at 37 °C (Binder, Germany). For the assay, HEK 293 cells were seeded in a 96-well plate (Corning, NY, USA) in 100 μL of the complete culture medium, at a final density of 2 × 10^3^ cells/well, and were cultured for 24 h for proper attachment. Subsequently, the medium was removed, and the cells were exposed to a tested inhibitor at different concentrations for 72 h in 100 μL of the fresh complete culture medium. The sensitivity of the cells to the compounds was detected using the vital dye Alamar Blue (Invitrogen, San Diego, CA, USA). The Alamar Blue assay was performed according to the procedure described by O’Brien et al. [42]. After incubation with the tested bCSE inhibitors, 10 μL of the Alamar Blue reagent was added into each well, the cell culture plates were returned to the humidified incubator, and the fluorescence was read after 3 h. The plates were exposed to an excitation light of 545 nm wavelength, and emission at 600 nm was measured on the CLARIOstar Plus (BMG LABTECH GmbH, Ortenberg, Germany). To determine half-maximal cytotoxic concentration (CC_50_), a four-parameter sigmoidal curve model (GraphPad Prism 8 software) was fitted to the measurement data.

### 3.6. Lab Scale Preparation, Purification, and Validation of Proteins bCSE and hCSE

For the production of *S. aureus* cystathionine-γ-lyase (bCSE) and of human cystathionine-γ-lyase (hCSE), plasmids pSUMO:saCSE (Addgene # 54336) and pNIC28-Bsa4 (Addgene # 42365) were employed. Cultivation of a competent *E. coli* cell strain (Thermo Fisher Scientific, Waltham, MA, USA), transformed with a target plasmid, was carried out on a selective solid medium with kanamycin. Bacterial cells harboring the plasmid vector were cultured at 37 °C and 180 rpm until OD_600_ = 0.65. Then, isopropyl β-D1-thiogalactopyranoside (IPTG) was added to a final concentration of 1 mM. To obtain bCSE, the cells were cultured for 3.5 h at 37 °C; to obtain hCSE, the cells were cultured for 18–20 h at 25 °C. After centrifugation, cells were resuspended in a buffer composed of 50 mM Tris–HCl, at a pH of 8.0, and 300 mM sodium chloride. After that, the cells were lysed by ultrasonication, with a pulse duration of 15 s and an interval between pulses of 15 s. The amplitude of the ultrasonic vibrations was 50% of the maximum. After lysis, samples were centrifuged for 15 min (20,000× *g* at 4 ℃). The pH of the supernatant obtained after the centrifugation was adjusted to 7.5 by the addition of a 1 M solution of Tris–OH. Then, the test sample was subjected to filtration through a polyethersulfone membrane with a pore diameter of 0.22 μm.

Purification was performed using a chromatographic system (Akta Avant 150, Cytiva). The first purification step, by means of 10-His, was identical for bCSE and hCSE; the purification was carried out on a metal chelate affinity sorbent with an average particle size of 30 μm. A cell lysate was applied to the sorbent bearing an immobilized chelating ligand (nitrilotriacetic acid), and the desired protein was eluted in an imidazole gradient. After that, the purified protein fractions were dialyzed against a 25 mM Tris–HCl buffer with a pH of 7.5.

After the first purification step, the proteins were subjected to proteolysis. bCSE was digested with ULP1 protease to remove the His6-SUMO tag; hCSE was processed with TEV protease to remove the His6-TEV tag. Next, the samples were applied to a strong anion exchange sorbent with an average particle size of 90 μm and functional quaternary ammonium groups immobilized on the particles. After anion exchange chromatography, samples were dialyzed overnight against storage buffer (0.02 M Tris-HCl pH 8.0, 0.100 M NaCl, 1 mM DTT, and 50% [*v*/*v*] of glycerol), flash frozen, and stored in a freezer at −80 °C.

The molecular weight of both proteins at each stage was confirmed by the classic method of polyacrylamide gel electrophoresis. Molecular weight validation was performed on a maXis 4G mass spectrometer (Bruker Daltonik, Bremen, Germany). bCSE was identical to sequence A0A2Y2FJW5 in the Uniprot database (https://www.uniprot.org/, (accessed on 17 May 2022)), whereas hCSE was identical to sequence P32929.

### 3.7. Other Methods and Funnel Metadynamics

#### 3.7.1. Parameter Sets

Three freely available modern force fields, Amber19sb, were used. All ligands was parameterized with acpype, where atom point charges were derived from 6-31G* ab initio calculations with the help of psiresp, and the other parameters were set with the GAFF2 forcefield. In the same manner, we performed the parametrization of pyridoxal-modified lysine [43].

#### 3.7.2. System Preparation

CSE was modeled based on coordinates from PDB ID 7MCU [13]. Protonation states of residues were predicted with PROPKA and verified manually [44]. The system was placed in a triclinic box with periodic boundary conditions and solvated with tip3p water molecules. Na^+^ and Cl^−^ ions were added to neutralize the net charge and to attain 0.15 M ionic strength. Systems were minimized with 5000 steps of steepest descent.

#### 3.7.3. Molecular Dynamics Simulations

The equilibration phase consisted of seven steps. First, an NVT run of 100 ps was performed while positionally restraining heavy atoms at 1000 kJ/(mol·nm^2^). A velocity rescale thermostat was utilized for temperature coupling [45]. Then, for five rounds of NPT equilibration of 100 ps, each restraint strength was gradually decreased as follows: 1000, 500, 200, 100, 10 kJ/(mol·nm^2^), respectively. A stochastic barostat was used for pressure control [46].

#### 3.7.4. Funnel Metadynamics

The funnel metadynamics setup was similar to the one described in Raniolo and Limongelli’s work [33]. A metadynamics potential of 0.5 kJ/mol was added once every 500 steps. Two collective variables were utilized. The first one was the distance between the COM (center of mass) of **NL2** in the binding site of an X-ray structure and the point solution 20 Å far from **NL2**’s position, which evaluates the projection of the ligand along the funnel line, and the second collective variable is distance from it. The use of a funnel metadynamics setup requires the correction of the resulting energy differences to turn them into a binding free energy estimate. The correction derives from the nature of the funnel-shaped restraint. Effective sampling is achieved by confining the unbound ligand within only a narrow cylinder, thus restraining it from diffusing throughout the solvent phase. Although this approach reduces the simulation time needed to achieve recrossing, it comes at a price: an unaccounted entropic contribution to the energy of an unbound state, and this contribution naturally depends on the radius of the cylinder. The equation for the calculation of a correction to account for this contribution can be found in the corresponding reference [33]. In the present study, for the cylinder, the correction amounts to 1.59 kcal/mol. We report the final calculated binding free energy together with an error estimate similar to the one used by Bhakat [47]. The width of the window for statistical analysis was 1000 ns.

### 3.8. Molecular Docking

#### 3.8.1. Protein and Ligand Structure Preparation

A set of models depicting the structure of bCSE together with low molecular weight inhibitors was utilized for calculations: 7MD0, 7MD6, 7MCU, 7MCY, and 7MCT [13]. All ligand–protein complexes were preprocessed with protein prepwizard [48]. This procedure is required to remove errors and inaccuracies in the protein and ligand structures; these problems can affect the results of calculations. This step also removes the crystallized solvents. The structure of the complex undergoes restrained minimization in force field OPLS4 [49]. Three-dimensional structures of the investigated compounds were generated in the OPLS4 forcefield as well. Ionization states and tautomers were predicted using the Epik module [50].

#### 3.8.2. GridBox Generation and the Docking Protocol

The Glide methodology was used for these calculations. GridBox was constructed for each computation iteration, based on the centroid of a reference ligand and amino acid capture inside a cubic region with a side of 13 Å [51]. Positional constraints were not applied. The variations in stereoisomers was restricted (they are presented as separate structures). The precision level was SP, with flexible docking. The ten best-fitting binding poses were included for each compound. Post-docking minimization was performed for each binding pose (crucial for correct hydrogen bonding). The best binding pose was selected based on the scoring function (GlideScore), solution clustering, and representation of reference structure pharmacophore properties.

#### 3.8.3. The MM–GBSA Protocol

Previously obtained docking solutions were used for calculations of the Gibbs free energy value (ΔG) and of strained contacts in a protein–ligand complex. The solvent model was VSGB, with forcefield OPLS4. The protein structure was flexible at 5 Å around a ligand. The minimization method was the steepest descent (SD). When per-atom ΔG values were visualized, fixed ramping for energy values coloring in the range ±4 kcal/mol was applied. The prime software was used for MM–GBSA ΔG calculations [52,53].

## 4. Conclusions

Several new selective bCSE inhibitors were efficiently synthesized and evaluated for their antimicrobial activity against Gram-positive and Gram-negative bacteria. Pyrrole and indole-3-carboxylic acid derivatives showed little or no inhibition of bCSE and potentiating activity with all tested antibiotics. Compounds **2a**, **2b**, **3a**, **3b**, **4a**, and **4b**, in combination with antibiotics of the *β*-lactam class (penicillin, cephalosporin, and carbapenem families) or of fluoroquinolone or aminoglycoside classes, considerably suppress the growth of *S. aureus.* Molecular docking and molecular dynamics tandem results revealed a new mode of ligand binding in the substrate. From the findings, we can conclude that the presence of two bicyclic systems in the structure of bCSE inhibitors does not interfere with the binding and even facilitates it. The calculations indicate that there is an alternative site for the binding of the inhibitors to bCSE. In this case, the binding occurs at a junction of several monomers in the tetrameric form of the enzyme. At the same time, the studied compounds showed low disproportion of the binding, as evidenced by similar GlideScore values between the two sites, and the same was true for ΔG values. The analyzed binding sites are equivalent in terms of the energy of the interaction, with our set of active molecules possessing the described chemotype. Therefore, some hypotheses can be advanced: (1) a possibility of bCSE interaction with several molecules of the same ligand; (2) the presence of the same amino acid residues in the different active sites allow for the expansion of the pharmacophore hypothesis and therefore improves the design of new effective bCSE inhibitors. Judging by the results of enzymatic and microbiological assays and in silico modeling, **2a** was designated as a lead compound. This compound exerted distinct action against *A. baumannii*, *P. aeruginosa*, and MRSA by potentiating the antimicrobial activity of ampicillin, cefepime, and piperacillin + tazobactam. Favorable in vitro ADMET characteristics made the lead compound a suitable candidate for in vivo studies. It is worth mentioning that a wide range of heterocyclic derivatives of reported bCSE inhibitors can be synthesized for in-depth elucidation of structure–activity relationships by the newly developed efficient and simple synthetic protocol. Our results confirm that the inhibition of hydrogen sulfide–generating enzymes is a promising strategy to circumvent antibiotic resistance.

## Data Availability

Data are contained within the article and supplementary material.

## Supplementary Materials

The following supporting information can be downloaded at: https://www.mdpi.com/article/10.3390/ijms242216331/s1. References [54,55,56,57,58,59,60] are cited in the supplementary file.

## References

[1] De Kraker M.E.A., Stewardson A.J., Harbarth S. (2016). Will 10 Million People Die a Year Due to Antimicrobial Resistance by 2050?. PLoS Med..

[2] Gould K. (2016). Antibiotics: From Prehistory to the Present Day. J. Antimicrob. Chemother..

[3] WHO List of Bacteria for Which New Antibiotics Are Urgently Needed. https://www.who.int/en/news-room/detail/27-02-2017-who-publishes-list-of-bacteria-for-which-new-antibiotics-are-urgently-needed.

[4] Donadio S., Maffioli S., Monciardini P., Sosio M., Jabes D. (2010). Antibiotic Discovery in the Twenty-First Century: Current Trends and Future Perspectives. J. Antibiot..

[5] Fernandes P., Martens E., Bertrand D., Pereira D. (2016). The Solithromycin Journey—It Is All in the Chemistry. Bioorg. Med. Chem..

[6] Årdal C., Balasegaram M., Laxminarayan R., McAdams D., Outterson K., Rex J.H., Sumpradit N. (2020). Antibiotic Development—Economic, Regulatory and Societal Challenges. Nat. Rev. Microbiol..

[7] Bai H., Wang S., Capelo-Martínez J., Igrejas G. (2019). Antibiotic Modification Addressing Resistance. Antibiotic Drug Resistance.

[8] Douafer H., Andrieu V., Phanstiel O., Brunel J.M. (2019). Antibiotic Adjuvants: Make Antibiotics Great Again!. J. Med. Chem..

[9] Wright G.D. (2016). Antibiotic Adjuvants: Rescuing Antibiotics from Resistance. Trends Microbiol..

[10] Fasnacht M., Polacek N. (2021). Oxidative Stress in Bacteria and the Central Dogma of Molecular Biology. Front. Mol. Biosci..

[11] Mironov A., Seregina T., Nagornykh M., Luhachack L.G., Korolkova N., Lopes L.E., Kotova V., Zavilgelsky G., Shakulov R., Shatalin K. (2017). Mechanism of H_2_S-Mediated Protection against Oxidative Stress in Escherichia Coli. Proc. Natl. Acad. Sci. USA.

[12] Shatalin K., Shatalina E., Mironov A., Nudler E. (2011). H_2_S: A Universal Defense Against Antibiotics in Bacteria. Science.

[13] Shatalin K., Nuthanakanti A., Kaushik A., Shishov D., Peselis A., Shamovsky I., Pani B., Lechpammer M., Vasilyev N., Shatalina E. (2021). Inhibitors of Bacterial H_2_S Biogenesis Targeting Antibiotic Resistance and Tolerance. Science.

[14] Borisov V.B., Forte E. (2021). Impact of Hydrogen Sulfide on Mitochondrial and Bacterial Bioenergetics. Int. J. Mol. Sci..

[15] Asimakopoulou A., Panopoulos P., Chasapis C.T., Coletta C., Zhou Z., Cirino G., Giannis A., Szabo C., Spyroulias G.A., Papapetropoulos A. (2013). Selectivity of Commonly Used Pharmacological Inhibitors for Cystathionine β-Synthase (CBS) and Cystathionine γ-Lyase (CSE). Br. J. Pharmacol..

[16] Kudličková Z., Michalková R., Salayová A., Ksiažek M., Vilková M., Bekešová S., Mojžiš J. (2023). Design, Synthesis, and Evaluation of Novel Indole Hybrid Chalcones and Their Antiproliferative and Antioxidant Activity. Molecules.

[17] Citarella A., Moi D., Pedrini M., Pérez-Peña H., Pieraccini S., Dimasi A., Stagno C., Micale N., Schirmeister T., Sibille G. (2023). Synthesis of SARS-CoV-2 M pro Inhibitors Bearing a Cinnamic Ester Warhead with in Vitro Activity against Human Coronaviruses. Org. Biomol. Chem..

[18] Jagadeesan S., Karpagam S. (2023). Novel Series of N-Acyl Substituted Indole Based Piperazine, Thiazole and Tetrazoles as Potential Antibacterial, Antifungal, Antioxidant and Cytotoxic Agents, and Their Docking Investigation as Potential Mcl-1 Inhibitors. J. Mol. Struct..

[19] Peng B., Chen W., Liu C., Rosser E.W., Pacheco A., Zhao Y., Aguilar H.C., Xian M. (2014). Fluorescent Probes Based on Nucleophilic Substitution-Cyclization for Hydrogen Sulfide Detection and Bioimaging. Chem. Eur. J..

[20] Dwyer D.J., Kohanski M.A., Hayete B., Collins J.J. (2007). Gyrase Inhibitors Induce an Oxidative Damage Cellular Death Pathway in Escherichia Coli. Mol. Syst. Biol..

[21] Kohanski M.A., Dwyer D.J., Hayete B., Lawrence C.A., Collins J.J. (2007). A Common Mechanism of Cellular Death Induced by Bactericidal Antibiotics. Cell.

[22] Qin H.-L., Liu J., Fang W.-Y., Ravindar L., Rakesh K.P. (2020). Indole-Based Derivatives as Potential Antibacterial Activity against Methicillin-Resistance Staphylococcus Aureus (MRSA). Eur. J. Med. Chem..

[23] McWilliam H., Li W., Uludag M., Squizzato S., Park Y.M., Buso N., Cowley A.P., Lopez R. (2013). Analysis Tool Web Services from the EMBL-EBI. Nucleic Acids Res..

[24] Jumper J., Evans R., Pritzel A., Green T., Figurnov M., Ronneberger O., Tunyasuvunakool K., Bates R., Žídek A., Potapenko A. (2021). Highly Accurate Protein Structure Prediction with AlphaFold. Nature.

[25] Steegborn C., Clausen T., Sondermann P., Jacob U., Worbs M., Marinkovic S., Huber R., Wahl M.C. (1999). Kinetics and Inhibition of Recombinant Human Cystathionine γ-Lyase. J. Biol. Chem..

[26] Waring M.J. (2010). Lipophilicity in Drug Discovery. Expert Opin. Drug Discov..

[27] Słoczyńska K., Gunia-Krzyżak A., Koczurkiewicz P., Wójcik-Pszczoła K., Żelaszczyk D., Popiół J., Pękala E. (2019). Metabolic Stability and Its Role in the Discovery of New Chemical Entities. Acta Pharm..

[28] Gardiner P., Cox R.J., Grime K. (2019). Plasma Protein Binding as an Optimizable Parameter for Acidic Drugs. Drug Metab. Dispos..

[29] Smith D.A., Di L., Kerns E.H. (2010). The Effect of Plasma Protein Binding on in Vivo Efficacy: Misconceptions in Drug Discovery. Nat. Rev. Drug Discov..

[30] Messerschmidt A., Worbs M., Steegborn C., Wahl M.C., Huber R., Laber B., Clausen T. (2003). Determinants of Enzymatic Specificity in the Cys-Met-Metabolism PLP-Dependent Enzyme Family: Crystal Structure of Cystathionine γ-Lyase from Yeast and Intrafamiliar Structure Comparison. Biol. Chem..

[31] Kaplan M.M., Flavin M. (1966). Cystathionine γ-Synthetase of Salmonella. J. Biol. Chem..

[32] Limongelli V., Bonomi M., Parrinello M. (2013). Funnel Metadynamics as Accurate Binding Free-Energy Method. Proc. Natl. Acad. Sci. USA.

[33] Raniolo S., Limongelli V. (2020). Ligand Binding Free-Energy Calculations with Funnel Metadynamics. Nat. Protoc..

[34] Abraham M.J., Murtola T., Schulz R., Páll S., Smith J.C., Hess B., Lindahl E. (2015). GROMACS: High Performance Molecular Simulations through Multi-Level Parallelism from Laptops to Supercomputers. SoftwareX.

[35] The PLUMED consortium (2019). Promoting Transparency and Reproducibility in Enhanced Molecular Simulations. Nat. Methods.

[36] Gottlieb H.E., Kotlyar V., Nudelman A. (1997). NMR Chemical Shifts of Common Laboratory Solvents as Trace Impurities. J. Org. Chem..

[37] Fulmer G.R., Miller A.J.M., Sherden N.H., Gottlieb H.E., Nudelman A., Stoltz B.M., Bercaw J.E., Goldberg K.I. (2010). NMR Chemical Shifts of Trace Impurities: Common Laboratory Solvents, Organics, and Gases in Deuterated Solvents Relevant to the Organometallic Chemist. Organometallics.

[38] Alferova V.A., Maviza T.P., Biryukov M.V., Zakalyukina Y.V., Lukianov D.A., Skvortsov D.A., Vasilyeva L.A., Tashlitsky V.N., Polshakov V.I., Sergiev P.V. (2022). Biological Evaluation and Spectral Characterization of a Novel Tetracenomycin X Congener. Biochimie.

[39] Osterman I.A., Wieland M., Maviza T.P., Lashkevich K.A., Lukianov D.A., Komarova E.S., Zakalyukina Y.V., Buschauer R., Shiriaev D.I., Leyn S.A. (2020). Tetracenomycin X Inhibits Translation by Binding within the Ribosomal Exit Tunnel. Nat. Chem. Biol..

[40] Yang Y., Zhao Y., Yu A., Sun D., Yu L.X., Qui Y., Chen Y., Zhang G., Yu L., Mantri R. (2017). Oral Drug Absorption. Developing Solid Oral Dosage Forms.

[41] Volpe D.A. (2008). Variability in Caco-2 and MDCK Cell-Based Intestinal Permeability Assays. J. Pharm. Sci..

[42] O’Brien J., Wilson I., Orton T., Pognan F. (2000). Investigation of the Alamar Blue (Resazurin) Fluorescent Dye for the Assessment of Mammalian Cell Cytotoxicity. Eur. J. Biochem..

[43] Connolly M.L. (1983). Analytical Molecular Surface Calculation. J. Appl. Crystallogr..

[44] Søndergaard C.R., Olsson M.H.M., Rostkowski M., Jensen J.H. (2011). Improved Treatment of Ligands and Coupling Effects in Empirical Calculation and Rationalization of pKa Values. J. Chem. Theory Comput..

[45] Bussi G., Donadio D., Parrinello M. (2007). Canonical Sampling through Velocity Rescaling. J. Chem. Phys..

[46] Bernetti M., Bussi G. (2020). Pressure Control Using Stochastic Cell Rescaling. J. Chem. Phys..

[47] Bhakat S., Söderhjelm P. (2017). Resolving the Problem of Trapped Water in Binding Cavities: Prediction of Host–Guest Binding Free Energies in the SAMPL5 Challenge by Funnel Metadynamics. J. Comput. Aided Mol. Des..

[48] Madhavi Sastry G., Adzhigirey M., Day T., Annabhimoju R., Sherman W. (2013). Protein and Ligand Preparation: Parameters, Protocols, and Influence on Virtual Screening Enrichments. J. Comput. Aided Mol. Des..

[49] Lu C., Wu C., Ghoreishi D., Chen W., Wang L., Damm W., Ross G.A., Dahlgren M.K., Russell E., Von Bargen C.D. (2021). OPLS4: Improving Force Field Accuracy on Challenging Regimes of Chemical Space. J. Chem. Theory Comput..

[50] Shelley J.C., Cholleti A., Frye L.L., Greenwood J.R., Timlin M.R., Uchimaya M. (2007). Epik: A Software Program for pKa Prediction and Protonation State Generation for Drug-like Molecules. J. Comput. Aided Mol. Des..

[51] Repasky M.P., Shelley M., Friesner R.A. (2007). Flexible Ligand Docking with Glide. Curr. Protoc. Bioinform..

[52] Suenaga A., Okimoto N., Hirano Y., Fukui K. (2012). An Efficient Computational Method for Calculating Ligand Binding Affinities. PLoS ONE.

[53] Li J., Abel R., Zhu K., Cao Y., Zhao S., Friesner R.A. (2011). The VSGB 2.0 model: A next generation energy model for high resolution protein structure modeling. Proteins.

[54] Subhas Bose D., Idrees M., Todewale I.K., Jakka N.M., Venkateswara Rao J. (2012). Hybrids of Privileged Structures Benzothiazoles and Pyrrolo[2,1-c] [1,4]Benzodiazepin-5-One, and Diversity-Oriented Synthesis of Benzothiazoles. Eur. J. Med. Chem..

[55] Yang T., Li X., Deng S., Qi X., Cong H., Cheng H.-G., Shi L., Zhou Q., Zhuang L. (2022). From N–H Nitration to Controllable Aromatic Mononitration and Dinitration─The Discovery of a Versatile and Powerful *N*-Nitropyrazole Nitrating Reagent. JACS.

[56] Brown F.J., Cronk L.A., Aharony D., Snyder D.W. (1992). 1,3,6-Trisubstituted Indoles as Peptidoleukotriene Antagonists: Benefits of a Second, Polar, Pyrrole Substituent. J. Med. Chem..

[57] Houck H.A., Blasco E., Du Prez F.E., Barner-Kowollik C. (2019). Light-Stabilized Dynamic Materials. J. Am. Chem. Soc..

[58] Young B.M., Rossi P., Slavish P.J., Cui Y., Sowaileh M., Das J., Kalodimos C.G., Rankovic Z. (2021). Synthesis of Isotopically Labeled, Spin-Isolated Tyrosine and Phenylalanine for Protein NMR Applications. Org. Lett..

[59] Verma A.K., Fatima K., Dudi R.K., Tabassum M., Iqbal H., Kumar Y., Luqman S., Mondhe D.M., Chanda D., Khan F. (2020). Antiproliferative Activity of Diarylnaphthylpyrrolidine Derivative via Dual Target Inhibition. Eur. J. Med. Chem..

[60] Portolani C., Centonze G., Luciani S., Pellegrini A., Righi P., Mazzanti A., Ciogli A., Sorato A., Bencivenni G. (2022). Synthesis of Atropisomeric Hydrazides by One-Pot Sequential Enantio- and Diastereoselective Catalysis. Angew. Chem. Int. Ed..

