# Supplementary material for: Naphthyl-Substituted Indole and Pyrrole Carboxylic Acids as Effective Antibiotic Potentiators—Inhibitors of Bacterial Cystathionine γ-Lyase
Source: Int J Mol Sci. 2023 Nov 15;24(22):16331. doi: 10.3390/ijms242216331 (PMC10671052; doi:10.3390/ijms242216331)
Supplement: Supplementary file 1 [file ijms-24-16331-s001.zip › ijms-2694630-supplementary.pdf]

Supporting Information

Naphthyl-Substituted Indole and Pyrrole Carboxylic Acids as Effective Antibiotic Potentiators - Inhibitors of bCSE

Andrey S. Kuzovlev [1,*], Mikhail D. Zybalov [1], Andrey V. Golovin [2,3], Maxim A. Gureev [3,4], Mariia A. Kasatkina [1], Mikhail V. Biryukov [1,5], Albina R. Belik [1], Sergey A. Silonov [1,6], Maxim A. Yunin [1], Nailya A. Zigangirova [7], Vasiliy V. Reshetnikov [1,8], Yulia E. Isakova [1], Yuri B. Porozov [3,4], Roman A. Ivanov [1]

[1] Translational Medicine Research Center, Sirius University of Science and Technology, Olympic Ave. 1, 354340 Sochi, Russia; zybalov.mikhail@gmail.com (M.D.Z.); kasatkina.ma@talantiuspeh.ru (M.A.K.); belik.ar@talantiuspeh.ru (A.R.B.); yunin.ma@talantiuspeh.ru (M.A.Y.); isakova.ye@talantiuspeh.ru (Y.E.I.); ivanov.ra@talantiuspeh.ru (R.A.I.)

[2] Faculty of Bioengineering and Bioinformatics, Lomonosov Moscow State University, 1/73 Leninskie gori St., 119234 Moscow, Russia; golovin.av@talantiuspeh.ru

[3] Laboratory of bioinformatics, Center of AI and information technologies, Sirius University of Science and Technology, Olympic Ave. 1, 354340 Sochi, Russia; gureev_m_a@staff.sechenov.ru

[4] Laboratory of bio- and chemoinformatics, Institute of Biodesign and Modeling of Complex Systems, I.M. Sechenov First Moscow State Medical University, 8/2 Trubetskaya, Moscow, 119991, Russia; porozov_yu_b@staff.sechenov.ru

[5] Faculty of Biology, Lomonosov Moscow State University, 1/12 Leninskie gori St., 119234 Moscow, Russia; biryukov.mv@talantiuspeh.ru

[6] Laboratory of Structural Dynamics, Stability and Folding of Proteins, Institute of Cytology, Russian Academy of Sciences, 4 Tikhoretsky Ave., 194064 St. Petersburg, Russia; silonov.sa@talantiuspeh.ru

[7] Medical Microbiology Department, Laboratory of chlamydiosis, National Research Center for Epidemiology and Microbiology named after N. F. Gamaleya, 18 Gamaleya St., 123098 Moscow, Russia; zigangirova@mail.ru

[8] Institute of Cytology and Genetics, Siberian Branch of RAS, 10 Akademika Lavrentyeva, 630090 Novosibirsk, Russia; reshetnikov.vv@talantiuspeh.ru

***** Correspondence: a.s.kuzovlev@gmial.com

Synthesis and characterization

Synthesis of indole-6-carboxylic acid

4-Methyl-3-nitrobenzoic acid

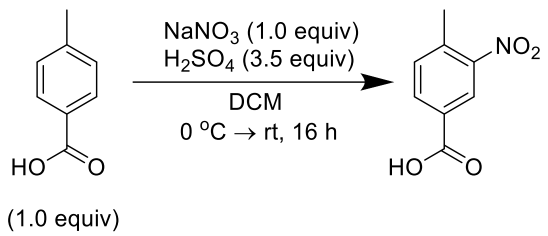

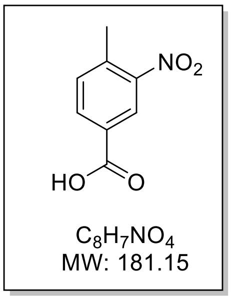

The compound was prepared according to a slightly altered procedure from the literature [54]. In a round-bottom flask (100 ml), to a solution of *p*-toluic acid (5.00 g, 36.72 mmol) in DCM (40 ml), $NaNO_3$ (2.90 g, 36.72 mmol) was added with stirring. After 10 min, H_2SO_4 (7.03 ml, 128.53 mmol) was introduced dropwise in an inert atmosphere at 0 °C. The reaction mixture was slowly heated to room temperature and stirred until completion (monitored by TLC) for 16 h. Next, the reaction mixture was poured into 200 ml of cold water. The organic layer was washed three times with water and brine, dried over $MgSO_4$, filtered, and concentrated in vacuum thereby affording the title product as a yellow oil (6.50 g, 100%). Spectral and physical data were in agreement with the literature [54].

^{1}H NMR (300 MHz, DMSO-*d6*) δ 13.25 (s, 1H), 8.41 (d, J = 1.8 Hz, 1H), 8.12 (dd, J = 7.9, 1.8 Hz, 1H), 7.63 (d, J = 8.0 Hz, 1H), 2.58 (s, 3H).

^{13}C NMR (75 MHz, DMSO-*d6*) δ 165.49, 148.84, 137.64, 133.39, 133.35, 130.06, 124.94, 19.66.

Methyl 4-methyl-3-nitrobenzoate

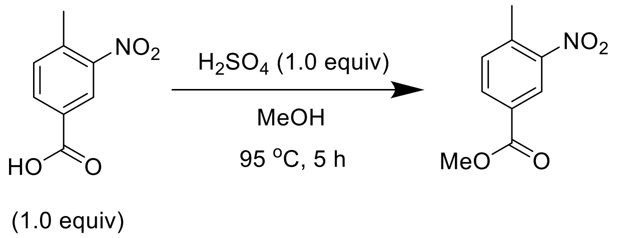

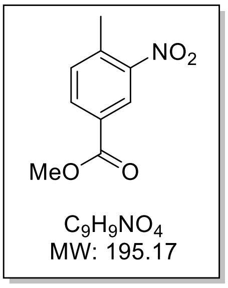

In a round-bottom flask, 4-methyl-3-nitrobenzoic acid (6.50 g, 36.72 mmol) was dissolved in methanol (30 ml); then H_2SO_4 (1.90 ml, 36.7 mmol) was added to the solution. The resulting mixture was refluxed in an oil bath (95 °C) for 5 h. The reaction mixture was evaporated in a rotary evaporator; poured into 30 ml of water; extracted with EtOAc (3 × 30 ml); washed with water, a saturated $NaHCO_3$ solution, and brine; dried over Na_2SO_4; and concentrated *in vacuo*. The residue was purified by FCC on silica gel (in a hexane/EtOAc mixture at 94:6) to obtain the title product as a yellow solid (3.21 g, 49%). Spectral and physical data were consistent with the literature [55].

^{1}H NMR (300 MHz, DMSO-*d6*) δ 8.42 (d, J = 1.8 Hz, 1H), 8.14 (dd, J = 8.0, 1.8 Hz, 1H), 7.67 (d, J = 8.0 Hz, 1H), 3.90 (s, 3H), 2.59 (s, 3H).

^{13}C NMR (75 MHz, DMSO-*d6*) δ 164.47, 148.86, 138.15, 133.56, 133.14, 128.72, 124.81, 52.58, 19.66.

Methyl (E)-4-(2-(dimethylamino)vinyl)-3-nitrobenzoate

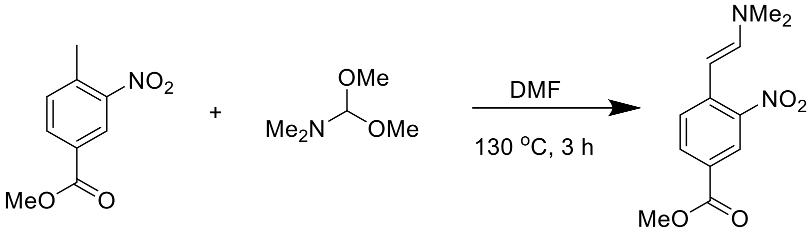

(1.0 equiv) (3.0 equiv)

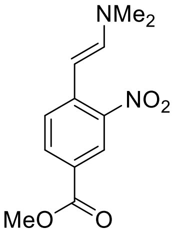

$C_{12}H_{14}N_2O_4$
MW: 250.25

The compound was prepared according to a slightly altered procedure from the literature [56]. In a round-bottom flask, methyl 4-methyl-3-nitrobenzoate (3.20 g, 16.47 mmol) was dissolved in DMF (20 ml); then dimethyl acetal of DMF (6.60 ml, 49.41 mmol) was added. The resulting solution was stirred and refluxed in an oil bath (130 °C) for 3 h. The reaction mixture was evaporated *in vacuo* thus yielding the title product as a red crumbly powder (3.40 g, 83%). Spectral and physical data were in agreement with the literature [56].

^{1}H NMR (400 MHz, DMSO-*d6*) δ 8.29 (t, J = 1.1 Hz, 1H), 7.78 (d, J = 1.1 Hz, 2H), 7.74 (d, J = 13.2 Hz, 1H), 5.77 (d, J = 13.2 Hz, 1H), 3.83 (s, 3H), 2.97 (s, 6H).

^{13}C NMR (101 MHz, DMSO-*d6*) δ 164.88, 149.15, 142.08, 140.57, 131.61, 126.86, 123.36, 121.01, 88.44, 52.00.

Methyl 1H-indole-6-carboxylate

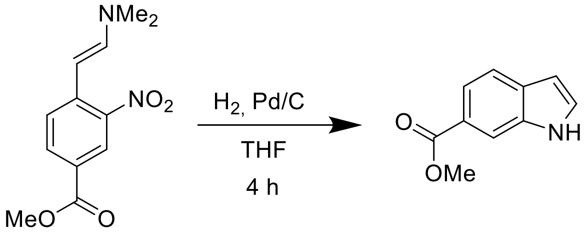

(1.0 equiv)

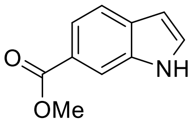

$C_{10}H_9NO_2$
MW: 175.19

The compound was prepared according to a slightly modified procedure from the literature [56]. Into a two-neck round-bottom flask charged with a solution of methyl (E)-4-(2-(dimethylamino)vinyl)-3-nitrobenzoate (3.40 g, 13.55 mmol) in THF (60 ml), 10% Pd/C (1.33 g, 50% water) was added. Hydrogen was bubbled through the solution for 4 h until no starting compound remained. The catalyst was filtered out in a plug of celite, and the filtrate was evaporated *in vacuo*. The dry residue was dissolved in ethyl acetate and poured into 5% HCl. The aqueous layer was extracted with EtOAc (3 × 15 ml). The combined organic layers were washed with water, a saturated $NaHCO_3$ solution, and brine, then dried over Na_2SO_4. After filtration and evaporation of the solvent, the residue was purified by column chromatography in isocratic mode (eluent: DCM) to obtain the pure title product as a white solid (2.10 g, 88%). Spectral and physical data were consistent with the literature [56].

^{1}H NMR (400 MHz, DMSO-*d*$_6$) δ 11.48 (s, 1H), 8.08 (d, J = 1.2 Hz, 1H), 7.62 (s, 2H), 7.59 (t, J = 2.8 Hz, 1H), 6.53 (t, J = 2.3 Hz, 1H), 3.85 (s, 3H).

^{13}C NMR (101 MHz, DMSO-*d*$_6$) δ 167.24, 135.10, 131.29, 129.21, 121.97, 119.78, 119.45, 113.39, 101.51, 51.69.

1H-indole-6-carboxylic acid

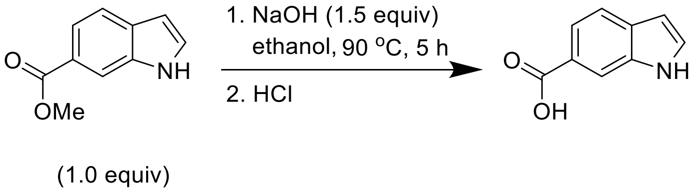

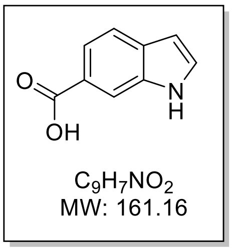

In a round-bottom flask, to a solution of methyl 1H-indole-6-carboxylate (1.00 g, 5.71 mmol) in ethanol (20 ml), NaOH (1.5 M in water, 4.95 ml, 7.42 mmol) was added. The resultant solution was stirred and refluxed in an oil bath (90 °C) for 5 h. After that, the mixture was cooled to room temperature, evaporated *in vacuo*, dissolved in 20 ml of water, and extracted with DCM (3 × 15 ml). The aqueous layer was acidified with HCl to pH 3–4 leading to the formation of an abundant white precipitate. The residue was filtered out, washed four times with 5 ml of distilled water, and dried under reduced pressure overnight thereby yielding the final desired compound as a white solid (0.91 g, 99%). Spectral and physical data were in agreement with the literature [56].

^{1}H NMR (400 MHz, DMSO-*d*$_6$) δ 12.45 (s, 1H), 11.43 (s, 1H), 8.06 (d, J = 1.3 Hz, 1H), 7.60 (d, J = 1.5 Hz, 2H), 7.56 (t, J = 2.8 Hz, 1H), 6.51 (ddd, J = 2.9, 1.9, 0.9 Hz, 1H).

^{13}C NMR (101 MHz, DMSO-*d*$_6$) δ 168.35, 135.16, 131.04, 128.85, 123.12, 119.77, 119.56, 113.51, 101.43.

Synthesis of α-(bromomethyl)naphthalene

Methyl 1-naphthoate

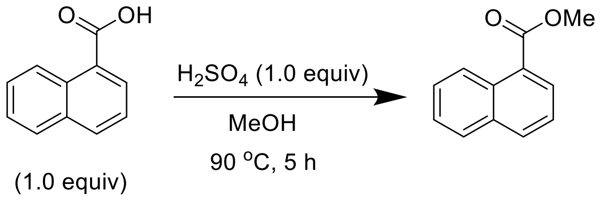

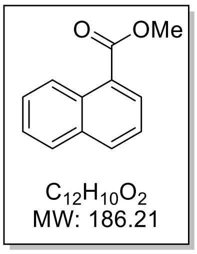

The compound was prepared according to a slightly altered procedure from the literature [57]. In a round-bottom flask, 1-naphthoic acid (5.00 g, 29.04 mmol) was dissolved in methanol (25 ml), then concentrated sulfuric acid (1.55 ml, 29.04 mmol) was added to the solution. The resulting mixture was stirred at reflux in an oil bath (90 °C) for 5 h. The reaction mixture was evaporated *in vacuo*, dissolved in DCM (20 ml), and washed with water (20 ml). The aqueous layer was extracted with DCM (3 × 15 ml). The combined organic layers were washed with water, a saturated $NaHCO_3$ solution, and brine and dried with Na_2SO_4. The obtained dry organic phase was concentrated *in vacuo* thus affording the pure title product as a faint yellow oil (5.01 g, 93%). Spectral and physical data were consistent with the literature [57].

^{1}**H NMR** (300 MHz, Chloroform-*d*) δ = 8.91 (ddt, J=8.6, 1.3, 0.8, 1H), 8.19 (dd, J=7.3, 1.4, 1H), 8.02 (dt, J=8.3, 1.3, 1H), 7.88 (ddt, J=8.1, 1.4, 0.6, 1H), 7.62 (ddd, J=8.6, 6.9, 1.6, 1H), 7.57–7.52 (m, 1H), 7.52–7.47 (m, 1H), 4.01 (s, 3H).

^{13}**C NMR** (75 MHz, Chloroform-*d*) δ 168.18, 134.02, 133.49, 131.51, 130.34, 128.68, 127.89, 127.30, 126.35, 125.99, 124.63, 52.25.

Naphthalen-1-ylmethanol

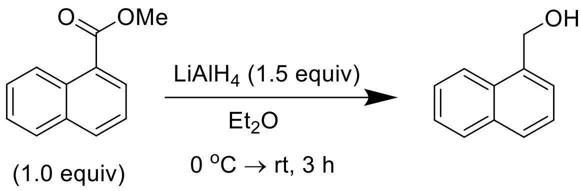

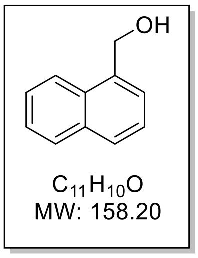

The compound was prepared according to a slightly modified procedure from the literature [58]. Into a round-bottom flask charged with a cooled (ice bath) suspension of $LiAlH_4$ (0.31 g, 8.06 mmol) in dry diethyl ether (15 ml), a solution of methyl 1-naphthoate (1.00 g, 5.37 mmol) in diethyl ether (5 ml) was added dropwise. After stirring at room temperature for 3 h, TLC (in a hexane/ethyl acetate mixture at 4:1) showed complete conversion of the starting material. The excess of $LiAlH_4$ was quenched with 30 ml of ethyl acetate followed by dropwise addition of 10% sulfuric acid (40 ml). Stirring was continued until all solids disappeared, and then the layers were separated. The aqueous layer was extracted with EtOAc (3 × 15 ml), and the combined organic layers were washed with water, a saturated $NaHCO_3$ solution, and brine and dried with Na_2SO_4. The obtained dry organic phase was concentrated *in vacuo* thereby affording the pure title product as a light-yellow oil (0.58 g, 68%). Spectral and physical data were consistent with the literature [59].

^{1}**H NMR** (300 MHz, DMSO-*d*$_6$) δ 8.11–8.06 (m, 1H), 7.96–7.89 (m, 1H), 7.82 (ddt, J = 8.1, 1.4, 0.7 Hz, 1H), 7.59–7.43 (m, 4H), 5.29 (t, J = 5.5 Hz, 1H), 4.97 (d, J = 5.5 Hz, 2H).

^{13}**C NMR** (75 MHz, DMSO-*d*$_6$) δ 137.78, 133.11, 130.65, 128.28, 127.20, 125.82, 125.57, 125.37, 124.18, 123.68, 61.13.

1-(bromomethyl)naphthalene

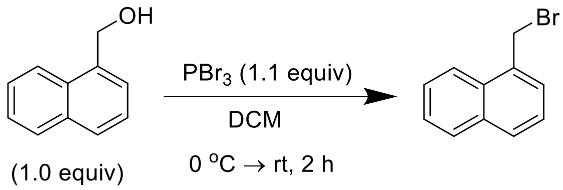

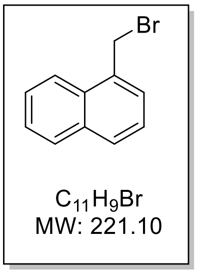

The compound was prepared according to a slightly altered procedure from the literature [60]. To a solution of naphthalen-1-ylmethanol (2.30 g, 14.54 mmol) in DCM (150 ml), phosphorus tribromide (1.52 ml, 15.99 mmol) was slowly added at 0 °C under N_2. After stirring at room temperature for 2 h, TLC analysis (in a hexane/ethyl acetate mixture at 4:1) revealed complete conversion of the starting material, then the reaction was stopped by slow addition of distilled water. The two phases were separated, the aqueous layer was extracted with DCM (3 × 15 ml), and the combined organic layers were washed with water, a saturated $NaHCO_3$ solution, and brine and

dried with Na_2SO_4. The resultant dry organic phase was concentrated *in vacuo* to obtain the pure title product as a yellow solid (0.77 g, 98%). Spectral and physical data were in agreement with the literature [59].

^{1}H NMR (300 MHz, DMSO-*d$_6$*) δ 8.19 (d, J = 8.4 Hz, 1H), 8.01–7.96 (m, 1H), 7.94 (d, J = 8.3 Hz, 1H), 7.71–7.68 (m, 1H), 7.67–7.62 (m, 1H), 7.57 (ddd, J = 8.1, 6.8, 1.3 Hz, 1H), 7.47 (dd, J = 8.2, 7.0 Hz, 1H), 5.22 (s, 2H).

^{13}C NMR (75 MHz, DMSO-*d$_6$*) δ 133.58, 133.51, 130.60, 129.46, 128.63, 128.10, 126.47, 126.22, 125.55, 123.93, 32.86.

Synthesis of β-(bromomethyl)naphthalene

Methyl 2-naphthoate

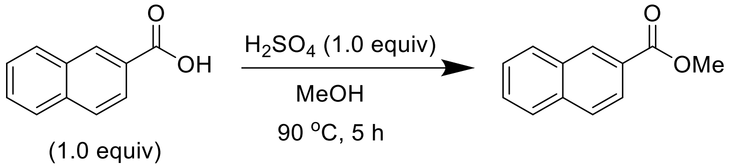

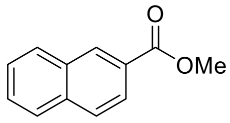

$C_{12}H_{10}O_2$
MW: 186.21

The compound was prepared according to a slightly altered procedure from the literature [57]. In a round-bottom flask, 2-naphthoic acid (5.00 g, 29.04 mmol) was dissolved in methanol (25 ml), then concentrated sulfuric acid (1.55 ml, 29.04 mmol) was added to the solution. The resulting mixture was stirred at reflux in an oil bath (90 °C) for 5 h. The reaction mixture was evaporated *in vacuo*, dissolved in DCM (20 ml), and washed with water (20 ml). The aqueous layer was extracted with DCM (3 × 15 ml). Next, the combined organic layers were washed with water, a saturated $NaHCO_3$ solution, and brine and dried with Na_2SO_4. The obtained dry organic phase was concentrated *in vacuo* thus affording the pure title product as a white solid (4.83 g, 89%). Spectral and physical data were consistent with the literature[57].

^{1}H NMR (300 MHz, Chloroform-*d*) δ 8.62 (s, 1H), 8.07 (dd, J = 8.6, 1.7 Hz, 1H), 7.99–7.91 (m, 1H), 7.88 (d, J = 8.5 Hz, 2H), 7.64–7.50 (m, 2H), 3.99 (s, 3H).

^{13}C NMR (75 MHz, Chloroform-*d*) δ 167.40, 135.69, 132.68, 131.21, 129.50, 128.36, 128.29, 127.91, 127.61, 126.77, 125.39, 52.33.

Naphthalen-2-ylmethanol

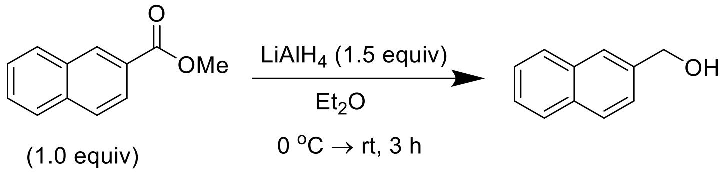

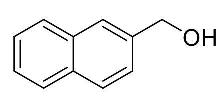

$C_{11}H_{10}O$
MW: 158.20

The compound was synthesized according to a slightly altered procedure from the literature [58]. Into a round-bottom flask charged with cooled (ice bath) suspension of $LiAlH_4$ (0.31 g, 8.06 mmol) in dry diethyl ether (15 ml), a solution of methyl 2-naphthoate (1.00 g, 5.37 mmol) in diethyl ether (5 ml) was added dropwise. After stirring at room temperature for 3 h, TLC analysis (in a hexane/ethyl acetate mixture at 4:1) showed complete conversion of the starting

material. The excess of $LiAlH_4$ was quenched with 30 ml of ethyl acetate followed by dropwise addition of 10% sulfuric acid (40 ml). Stirring was continued until all solids disappeared, and then the layers were separated. The aqueous layer was extracted with EtOAc (3 × 15 ml), and the combined organic layers were washed with water, a saturated $NaHCO_3$ solution, and brine and dried with Na_2SO_4. The resultant dry organic phase was concentrated *in vacuo* to obtain the pure title product as a white solid (0.77 g, 90%). Spectral and physical data were in agreement with the literature [59].

^{1}H NMR (300 MHz, DMSO-*d6*) δ 7.91–7.85 (m, 3H), 7.84–7.81 (m, 1H), 7.54–7.42 (m, 3H), 5.31 (t, J = 5.7 Hz, 1H), 4.68 (d, J = 5.7 Hz, 2H).

^{13}C NMR (75 MHz, DMSO-*d6*) δ 140.18, 132.91, 132.13, 127.56, 127.51, 127.48, 125.96, 125.40, 125.22, 124.26, 62.97.

2-(bromomethyl)naphthalene

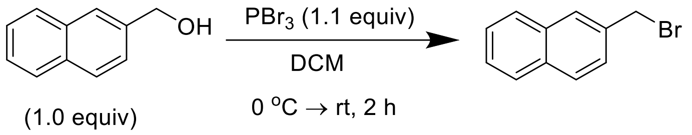

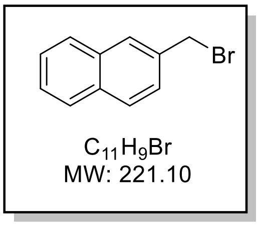

The compound was prepared according to a slightly altered procedure from the literature [60]. To a solution of naphthalen-2-ylmethanol (0.75 g, 4.71 mmol) in DCM (45 ml), phosphorus tribromide (0.49 ml, 5.18 mmol) was slowly added at 0 °C under N_2. After stirring at room temperature for 2 h, TLC analysis (in a hexane/ethyl acetate mixture at 4:1) revealed complete conversion of the starting material, then the reaction was stopped by slow addition of distilled water. The two phases were separated, the aqueous layer was extracted with DCM (3 × 15 ml), and the combined organic layers were washed with water, a saturated $NaHCO_3$ solution, and brine and dried with Na_2SO_4. The obtained dry organic phase was concentrated *in vacuo* thereby giving the pure title product as a yellow solid (0.53 g, 51%). Spectral and physical data were consistent with the literature [59].

^{1}H NMR (300 MHz, Chloroform-*d*) δ 7.87–7.79 (m, 4H), 7.55–7.46 (m, 3H), 4.68 (s, 2H).

^{13}C NMR (75 MHz, Chloroform-*d*) δ 135.24, 133.33, 133.24, 128.92, 128.11, 128.01, 127.87, 126.91, 126.72, 126.63, 34.18.

1-(naphthalen-1-ylmethyl)-1H-indole-3-carboxylic acid (1a)

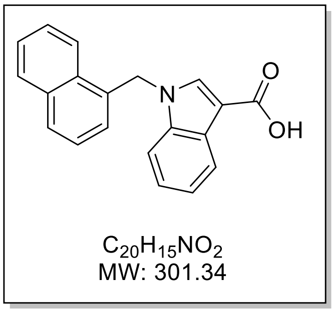

It was synthesized from 1H-indole-3-carboxylic acid (150.0 mg, 0.93 mmol), 1-(bromomethyl)naphthalene (308.7 mg, 1.40 mmol), and NaH (111.7 mg, 2.79 mmol) in DMF (5 ml) according to General procedure 1. The precipitate was filtered out, washed with distilled water, and dried under reduced pressure overnight thus affording **1a** as a white solid (142.1 mg, 51%): mp 238 °C.

^{1}H NMR (300 MHz, DMSO-*d6*) δ 12.04 (br.s, 1H), 8.18–8.12 (m, 1H), 8.08 (dd, J = 6.3, 2.9 Hz, 1H), 8.04–7.96 (m, 2H), 7.89 (d, J = 8.3 Hz, 1H), 7.61–7.52 (m, 3H), 7.45–7.38 (m, 1H), 7.25–7.18 (m, 2H), 6.97 (d, J = 7.1 Hz, 1H), 6.01 (s, 2H).

^{13}C NMR (75 MHz, DMSO-*d$_6$*) δ 165.49, 136.72, 135.30, 133.31, 132.47, 130.42, 128.67, 128.24, 126.62, 126.51, 126.12, 125.50, 124.95, 123.07, 122.48, 121.48, 120.96, 111.04, 107.13, 47.39.

HRMS (ESI$^+$) calcd. for $C_{20}H_{16}NO_2$ [M+H]$^+$: 302.1181; found: 302.1174. **HRMS** (ESI$^-$) calcd. for $C_{20}H_{14}NO_2$ [M-H]$^-$: 300.1025; found: 300.0995.

1-(Naphthalen-2-ylmethyl)-1H-indole-3-carboxylic acid (1b)

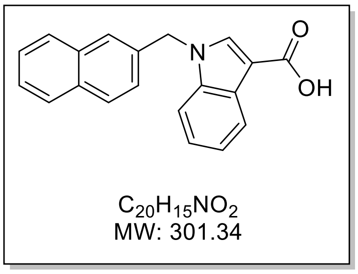

It was prepared from 1H-indole-3-carboxylic acid (72.90 mg, 0.45 mmol), 2-(bromomethyl)naphthalene (150.00 mg, 0.68 mmol), and NaH (42.20 mg, 1.13 mmol) in DMF (5 ml) according to General procedure 1. The precipitate was filtered off, washed with distilled water, and dried under reduced pressure overnight to obtain **1b** as a white solid (78.4 mg, 58%): mp 216 °C.

^{1}H NMR (300 MHz, DMSO-*d$_6$*) δ 12.08 (br.s, 1H), 8.29 (s, 1H), 8.07–8.01 (m, 1H), 7.86 (dd, J = 10.2, 6.5 Hz, 4H), 7.60–7.54 (m, 1H), 7.52–7.46 (m, 2H), 7.41 (dd, J = 8.5, 1.7 Hz, 1H), 7.21–7.14 (m, 2H), 5.67 (s, 2H).

^{13}C NMR (75 MHz, DMSO-*d$_6$*) δ 165.56, 136.34, 135.58, 134.69, 132.75, 132.32, 128.38, 127.67, 127.55, 126.68, 126.42, 126.13, 125.96, 125.34, 122.34, 121.37, 120.90, 111.10, 106.94, 49.75.

HRMS (ESI$^+$) calcd. for $C_{20}H_{16}NO_2$ [M+H]$^+$: 302.1181; found: 302.1179. **HRMS** (ESI$^-$) calcd. for $C_{20}H_{14}NO_2$ [M-H]$^-$: 300.1025; found: 300.0995.

Sodium 1-(naphthalen-1-ylmethyl)-1H-indole-3-carboxylate (1c)

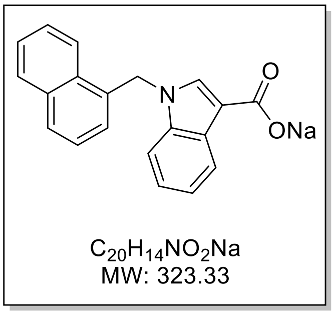

It was prepared from 1H-indole-3-carboxylic acid (150.00 mg, 0.93 mmol), 1-(bromomethyl)naphthalene (308.70 mg, 1.40 mmol), and NaH (111.70 mg, 2.79 mmol) in DMF (5 ml) according to General procedure 1 (without the acidification step). The precipitate was filtered out, washed with distilled water, and dried under reduced pressure overnight thereby affording **1c** as a light-brown solid (160.0 mg, 58%): mp 110 °C.

^{1}H NMR (300 MHz, DMSO-*d$_6$*) δ 8.32–8.28 (m, 1H), 8.15–8.10 (m, 1H), 7.97 (dd, J = 6.7, 2.7 Hz, 1H), 7.87 (d, J = 8.3 Hz, 1H), 7.58–7.53 (m, 3H), 7.42–7.36 (m, 2H), 7.06–7.01 (m, 2H), 6.94 (d, J = 6.1 Hz, 1H), 5.86 (s, 2H).

^{13}C NMR (75 MHz, DMSO-*d$_6$*) δ 169.72, 136.52, 133.39, 133.31, 132.00, 130.58, 128.61, 127.98, 126.46, 126.00, 125.45, 124.95, 123.21, 122.37, 120.79, 119.30, 116.85, 109.69, 46.95.

HRMS (ESI$^+$) calcd. for $C_{20}H_{16}NO_2$ [M+H]$^+$: 302.1181; found: 302.1176. **HRMS** (ESI$^-$) calcd. for $C_{20}H_{14}NO_2$ [M-H]$^-$: 300.1025; found: 300.0997.

1-(Naphthalen-1-ylmethyl)-1H-indole-5-carboxylic acid (3a)

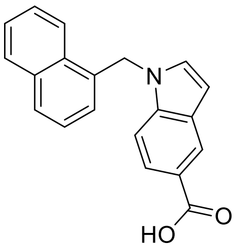

$C_{20}H_{15}NO_2$
MW: 301.34

It was prepared from 1H-indole-5-carboxylic acid (150.00 mg, 0.93 mmol), 1-(bromomethyl)naphthalene (308.70 mg, 1.40 mmol), and NaH (111.70 mg, 2.79 mmol) in DMF (5 ml) according to General procedure 1. The precipitate was filtered off, washed with distilled water, and dried under reduced pressure overnight to obtain **3a** as a brown solid (256.4 mg, 91%): mp 225 °C.

^{1}H NMR (400 MHz, DMSO-*d6*) δ 12.47 (br.s, 1H), 8.29 (d, J = 1.2 Hz, 1H), 8.18–8.15 (m, 1H), 7.97 (dd, J = 7.0, 2.4 Hz, 1H), 7.86 (d, J = 8.3 Hz, 1H), 7.73 (dd, J = 8.7, 1.6 Hz, 1H), 7.58 (ddd, J = 7.1, 5.0, 1.6 Hz, 2H), 7.55–7.51 (m, 2H), 7.41–7.35 (m, 1H), 6.81 (d, J = 6.7 Hz, 1H), 6.68 (d, J = 2.7 Hz, 1H), 5.98 (s, 2H).

^{13}C NMR (101 MHz, DMSO-*d6*) δ 168.23, 138.47, 133.29, 133.25, 130.82, 130.39, 128.61, 127.97, 127.76, 126.50, 126.07, 125.47, 124.30, 123.25, 123.14, 122.52, 121.87, 109.92, 102.78, 47.23.

HRMS (ESI$^+$) calcd. for $C_{20}H_{16}NO_2$ [M+H]$^+$: 302.1181; found: 302.1174. **HRMS** (ESI$^-$) calcd. for $C_{20}H_{14}NO_2$ [M-H]$^-$: 300.1025; found: 300.0993.

1-(Naphthalen-1-ylmethyl)-1H-indole-6-carboxylic acid (4a)

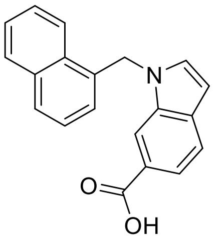

$C_{20}H_{15}NO_2$
MW: 301.34

It was prepared from 1H-indole-6-carboxylic acid (150.00 mg, 0.93 mmol), 1-(bromomethyl)naphthalene (308.70 mg, 1.40 mmol), and NaH (111.70 mg, 2.79 mmol) in DMF (5 ml) according to General procedure 1. The precipitate was filtered out, washed with distilled water, and dried under reduced pressure overnight thus giving **4a** as a brown solid (274.0 mg, 98%): mp 228 °C.

^{1}H NMR (400 MHz, DMSO-*d6*) δ 12.57 (s, 1H), 8.18 (d, J = 7.8 Hz, 1H), 8.07 (s, 1H), 8.01–7.96 (m, 1H), 7.86 (d, J = 8.2 Hz, 1H), 7.67 (s, 2H), 7.64 (d, J = 3.1 Hz, 1H), 7.62–7.56 (m, 2H), 7.37 (t, J = 7.7 Hz, 1H), 6.72 (d, J = 7.0 Hz, 1H), 6.63 (d, J = 2.9 Hz, 1H), 6.05 (s, 2H).

^{13}C NMR (101 MHz, DMSO-*d6*) δ 168.19, 135.47, 133.44, 133.26, 132.79, 131.70, 130.32, 128.61, 127.90, 126.49, 126.10, 125.48, 123.88, 123.64, 123.15, 120.26, 120.20, 112.19, 101.61, 47.19.

HRMS (ESI$^+$) calcd. for $C_{20}H_{16}NO_2$ [M+H]$^+$: 302.1181; found: 302.1176. **HRMS** (ESI$^-$) calcd. for $C_{20}H_{14}NO_2$ [M-H]$^-$: 300.1025; found: 300.0990.

1-(Naphthalen-2-ylmethyl)-1H-indole-5-carboxylic acid (4b)

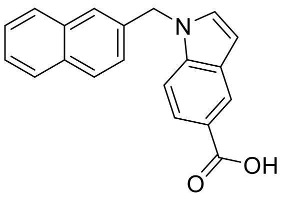

$C_{20}H_{15}NO_2$
MW: 301.34

It was prepared from 1H-indole-5-carboxylic acid (72.90 mg, 0.45 mmol), 2-(bromomethyl)naphthalene (150.00 mg, 0.68 mmol), and NaH (42.20 mg, 1.13 mmol) in DMF (5 ml) according to General procedure 1. The precipitate was filtered off, washed with distilled water, and dried under reduced pressure overnight to obtain **4b** as a brown solid (110.70 mg, 81%): mp 212 °C.

^{1}H NMR (300 MHz, DMSO-*d6*) δ 12.46 (br.s, 1H), 8.26 (d, J = 1.1 Hz, 1H), 7.88–7.80 (m, 3H), 7.75–7.66 (m, 3H), 7.58 (d, J = 8.7 Hz, 1H), 7.51 – 7.45 (m, 2H), 7.35 (dd, J = 8.5, 1.8 Hz, 1H), 6.67 (dd, J = 3.2, 0.6 Hz, 1H), 5.63 (s, 2H).

^{13}C NMR (75 MHz, DMSO-d_6) δ 168.20, 138.13, 135.42, 132.77, 132.27, 130.82, 128.29, 127.88, 127.62, 127.54, 126.38, 126.01, 125.56, 125.25, 123.17, 122.40, 121.80, 109.90, 102.66, 49.46.

HRMS (ESI$^+$) calcd. for $C_{20}H_{16}NO_2$ $[M+H]^+$: 302.1181; found: 302.1173. **HRMS** (ESI$^-$) calcd. for $C_{20}H_{14}NO_2$ $[M-H]^-$: 300.1025; found: 300.0984.

Ethyl 1-(naphthalen-1-ylmethyl)-1H-indole-2-carboxylate (2c)

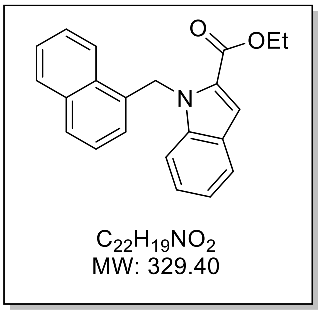

It was synthesized from ethyl 1H-indole-2-carboxylate (150.00 mg, 0.79 mmol), 1-(bromomethyl)naphthalene (262.90 mg, 1.19 mmol), and NaH (47.60 mg, 1.19 mmol) in DMF (5 ml) in accordance with General procedure 2. The residue was purified by FCC on silica gel (in a hexane/EtOAc gradient 20:1→ 15:1) thereby affording **2c** as a yellow oil (104.5 mg, 40%).

^{1}H NMR (300 MHz, DMSO-d_6) δ 8.28 (d, J = 8.4 Hz, 1H), 8.01–7.95 (m, 1H), 7.83–7.75 (m, 2H), 7.64 (ddd, J = 11.9, 8.0, 1.2 Hz, 2H), 7.49 (d, J = 0.8 Hz, 1H), 7.46–7.41 (m, 1H), 7.30–7.15 (m, 3H), 6.36 (s, 2H), 6.11–6.04 (m, 1H), 4.17 (q, J = 7.2 Hz, 2H), 1.16 (t, J = 7.1 Hz, 3H).

^{13}C NMR (75 MHz, DMSO-d_6) δ 160.99, 139.39, 134.38, 133.09, 129.90, 128.53, 127.56, 127.02, 126.33, 126.02, 125.53, 125.43, 125.37, 122.90, 122.58, 120.96, 120.92, 111.10, 110.74, 60.31, 45.39, 13.92.

HRMS (ESI$^+$) calcd. for $C_{22}H_{20}NO_2$ $[M+H]^+$: 330.1494; found: 330.1481.

Ethyl 1-(naphthalen-2-ylmethyl)-1H-indole-2-carboxylate (2d)

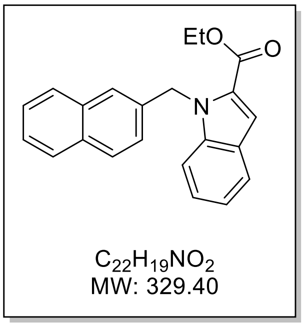

In was prepared from ethyl 1H-indole-2-carboxylate (300.00 mg, 1.58 mmol), 2-(bromomethyl)naphthalene (526.00 mg, 2.38 mmol), and NaH (95.20 mg, 2.38 mmol) in DMF (10 ml) according to General procedure 2. The residue was purified by FCC on silica gel (in hexane/EtOAc gradient 20:1→ 15:1) to obtain **2d** as yellow oil (501.10 mg, 96%).

^{1}H NMR (300 MHz, DMSO-d_6) δ 7.83 (t, J = 7.2 Hz, 2H), 7.75 (d, J = 7.8 Hz, 2H), 7.61 (d, J = 8.5 Hz, 1H), 7.50 (s, 1H), 7.47 – 7.41 (m, 3H), 7.30 (t, J = 7.7 Hz, 1H), 7.22 (d, J = 8.5 Hz, 1H), 7.15 (t, J = 7.5 Hz, 1H), 6.02 (s, 2H), 4.28 (q, J = 7.1 Hz, 2H), 1.28 (t, J = 7.1 Hz, 3H).

^{13}C NMR (75 MHz, DMSO-d_6) δ 161.28, 139.12, 136.14, 132.76, 132.11, 128.16, 127.55, 127.50, 127.32, 126.29, 125.82, 125.59, 125.29, 124.64, 124.52, 122.54, 120.86, 111.32, 110.74, 60.47, 47.36, 14.09.

HRMS (ESI$^+$) calcd. for $C_{22}H_{20}NO_2$ $[M+H]^+$: 330.1494; found: 330.1483.

Methyl 1-(naphthalen-2-ylmethyl)-1H-indole-6-carboxylate (3c)

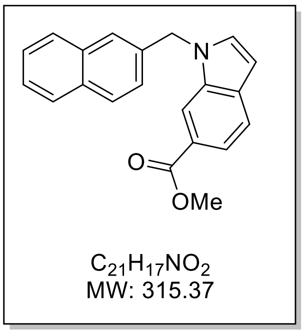

It was prepared from methyl 1H-indole-6-carboxylate (200.00 mg, 1.14 mmol), 2-(bromomethyl)naphthalene (378.80 mg, 1.71 mmol), and NaH (68.50 mg, 1.71 mmol) in DMF (5 ml) in accordance with General procedure 2. The residue was purified by FCC on silica gel (in a hexane/EtOAc mixture at 4:1) thus giving **3c** as a yellowish solid (129.9 mg, 65%): mp 123 ^{o}C.

^{1}H NMR (300 MHz, DMSO-*d$_6$*) δ 8.13 (q, J = 1.0 Hz, 1H), 7.89–7.85 (m, 2H), 7.81 (dd, J = 5.5, 3.2 Hz, 2H), 7.66 (dd, J = 3.3, 1.0 Hz, 3H), 7.50–7.46 (m, 2H), 7.32 (dd, J = 8.5, 1.8 Hz, 1H), 6.64 (dd, J = 3.1, 0.8 Hz, 1H), 5.71 (s, 2H), 3.80 (s, 3H).

^{13}C NMR (75 MHz, DMSO-*d$_6$*) δ 167.04, 135.55, 135.09, 133.13, 132.77, 132.25, 132.06, 128.32, 127.57, 127.54, 126.40, 125.99, 125.15, 124.98, 122.36, 120.41, 119.83, 112.01, 101.60, 51.74, 49.33.

HRMS (ESI$^+$) calcd. for $C_{21}H_{18}NO_2$ [M+H]$^+$: 316.1338; found: 316.1328.

Ethyl 1-(naphthalen-1-ylmethyl)-1H-pyrrole-2-carboxylate (5c)

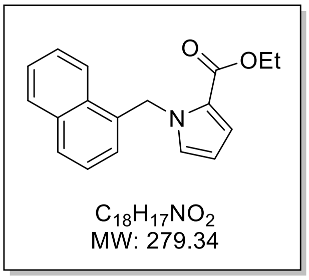

It was prepared from ethyl 1H-pyrrole-2-carboxylate (100.00 mg, 0.72 mmol), 1-(bromomethyl)naphthalene (190.90 mg, 1.08 mmol), and NaH (43.20 mg, 1.08 mmol) in DMF (5 ml) according to General procedure 2. The residue was purified by FCC on silica gel (in a hexane/EtOAc mixture at 94:6) to isolate **5c** as a colorless oil (129.9 mg, 65%).

^{1}H NMR (300 MHz, DMSO-*d$_6$*) δ 8.10 (d, J = 7.4 Hz, 1H), 8.00–7.94 (m, 1H), 7.83 (d, J = 8.2 Hz, 1H), 7.59 (ddd, J = 6.8, 4.5, 1.6 Hz, 2H), 7.41–7.34 (m, 1H), 7.20 (t, J = 2.1 Hz, 1H), 7.00 (dd, J = 3.9, 1.8 Hz, 1H), 6.49 (d, J = 7.1 Hz, 1H), 6.24 (dd, J = 3.9, 2.6 Hz, 1H), 6.06 (s, 2H), 4.07 (q, J = 7.1 Hz, 2H), 1.11 (t, J = 7.1 Hz, 3H).

^{13}C NMR (75 MHz, DMSO-*d$_6$*) δ 160.20, 135.11, 133.01, 130.33, 129.91, 128.53, 127.39, 126.43, 126.01, 125.58, 122.78, 122.40, 121.79, 118.03, 108.44, 59.35, 49.34, 14.09.

HRMS (ESI$^+$) calcd. for $C_{18}H_{18}NO_2$ [M+H]$^+$: 280.1338; found: 280.1331.

Ethyl 1-(naphthalen-2-ylmethyl)-1H-pyrrole-2-carboxylate (5d)

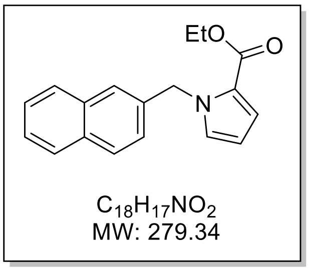

It was prepared from ethyl 1H-pyrrole-2-carboxylate (150.00 mg, 1.09 mmol), 2-(bromomethyl)naphthalene (357.50 mg, 1.62 mmol), and NaH (64.70 mg, 1.62 mmol) in DMF (5 ml) according to General procedure 2. The residue was purified by FCC on silica gel (hexane/EtOAc 94:6) to obtain **5d** as a colorless oil (192.3 mg, 64%).

^{1}H NMR (300 MHz, Chloroform-*d*) δ 7.82–7.75 (m, 3H), 7.51 (s, 1H), 7.48–7.43 (m, 2H), 7.28 (dd, J = 8.5, 1.7 Hz, 1H), 7.06 (dd, J = 3.9, 1.8 Hz, 1H), 6.96–6.89 (m, 1H), 6.22 (dd, J = 3.9, 2.6 Hz, 1H), 5.73 (s, 2H), 4.24 (q, J = 7.1 Hz, 2H), 1.30 (t, J = 7.1 Hz, 3H).

^{13}C NMR (75 MHz, Chloroform-*d*) δ 161.31, 136.00, 133.56, 132.94, 129.09, 128.55, 128.04, 127.80, 126.30, 126.01, 125.75, 125.18, 122.66, 118.47, 108.65, 59.96, 52.35, 14.50.

HRMS (ESI$^+$) calcd. for $C_{18}H_{18}NO_2$ [M+H]$^+$: 280.1338; found: 280.1331.

1-(Naphthalen-1-ylmethyl)-1H-indole-2-carboxylic acid (2a)

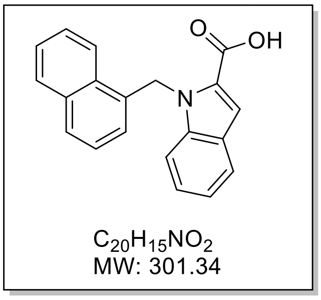

It was synthesized from ethyl 1-(naphthalen-1-ylmethyl)-1H-indole-2-carboxylate (95.00 mg, 0.29 mmol) and NaOH (1.5 M in water, 0.38 ml, 0.57 mmol) in ethanol (5 ml) according to General procedure 3. The precipitate was filtered out, washed with distilled water, and dried under reduced pressure overnight thus affording **2a** as a white solid (256.4 mg, 91%): mp 228 °C.

^{1}H NMR (400 MHz, DMSO-*d*$_6$) δ 12.89 (br.s, 1H), 8.29 (d, J = 8.3 Hz, 1H), 7.98 (d, J = 7.6 Hz, 1H), 7.77 (dd, J = 8.0, 5.2 Hz, 2H), 7.69–7.65 (m, 1H), 7.63–7.59 (m, 1H), 7.45–7.43 (m, 1H), 7.38 (d, J = 8.4 Hz, 1H), 7.26–7.21 (m, 2H), 7.18–7.13 (m, 1H), 6.39 (s, 2H), 6.08 (d, J = 7.1 Hz, 1H).

^{13}C NMR (101 MHz, DMSO-*d*$_6$) δ 162.66, 139.27, 134.54, 133.10, 129.96, 128.55, 126.99, 126.35, 126.02, 125.62, 125.47, 125.03, 122.98, 122.47, 121.09, 120.75, 111.08, 110.55, 45.22.

HRMS (ESI$^+$) calcd. for $C_{20}H_{16}NO_2$ [M+H]$^+$: 302.1181; found: 302.1176. **HRMS** (ESI$^-$) calcd. for $C_{20}H_{14}NO_2$ [M-H]$^-$: 300.1025; found: 300.0997.

1-(Naphthalen-2-ylmethyl)-1H-indole-2-carboxylic acid (2b)

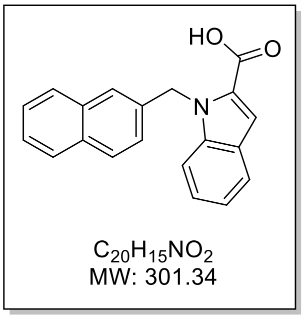

It was prepared from ethyl 1-(naphthalen-2-ylmethyl)-1H-indole-2-carboxylate (35.7 mg, 0.11 mmol) and NaOH (1.5 M in water, 0.10 ml, 0.14 mmol) in ethanol (2 ml) according to General procedure 3. The precipitate was filtered off, washed with distilled water, and dried under reduced pressure overnight thereby giving **2b** as a white solid (20.6 mg, 63%): mp 219 °C.

^{1}H NMR (300 MHz, DMSO-*d*$_6$) δ 12.98 (s, 1H), 7.85–7.80 (m, 2H), 7.77–7.71 (m, 2H), 7.59–7.55 (m, 1H), 7.51 (s, 1H), 7.45 (dd, J = 6.2, 3.3 Hz, 2H), 7.37 (d, J = 0.8 Hz, 1H), 7.29–7.20 (m, 2H), 7.16–7.10 (m, 1H), 6.05 (s, 2H).

^{13}C NMR (75 MHz, DMSO-*d*$_6$) δ 162.91, 138.99, 136.29, 132.75, 132.08, 128.21, 128.10, 127.50, 127.48, 126.25, 125.77, 125.65, 124.91, 124.69, 124.54, 122.39, 120.64, 111.24, 110.49, 47.15.

HRMS (ESI$^+$) calcd. for $C_{20}H_{16}NO_2$ [M+H]$^+$: 302.1181; found: 302.1175. **HRMS** (ESI$^-$) calcd. for $C_{20}H_{14}NO_2$ [M-H]$^-$: 300.1025; found: 300.1022.

1-(Naphthalen-2-ylmethyl)-1H-indole-6-carboxylic acid (3b)

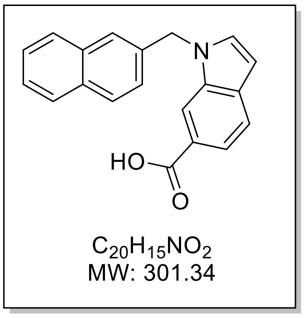

It was prepared from methyl 1-(naphthalen-1-ylmethyl)-1H-indole-6-carboxylate (300.00 mg, 0.95 mmol) and NaOH (1.5 M in water, 1.27 ml, 1.90 mmol) in ethanol (7 ml) in accordance with General procedure 3. The precipitate was filtered out, washed with distilled water, and dried under reduced pressure overnight to obtain **3b** as a white solid (234.5 mg, 82%): mp 208 °C.

^{1}H NMR (300 MHz, DMSO-*d*$_6$) δ 12.52 (s, 1H), 8.10 (q, J = 1.1 Hz, 1H), 7.89–7.84 (m, 2H), 7.83–7.77 (m, 2H), 7.69 (s, 1H), 7.65 (d, J = 0.9 Hz, 2H), 7.50–7.46 (m, 2H), 7.32 (dd, J = 8.5, 1.7 Hz, 1H), 6.63 (dd, J = 3.1, 0.8 Hz, 1H), 5.70 (s, 2H).

^{13}**C NMR** (75 MHz, DMSO-d_6) δ 168.14, 135.62, 135.13, 132.81, 132.26, 131.83, 128.31, 127.58, 127.56, 126.40, 125.99, 125.18, 125.01, 123.51, 120.17, 120.13, 112.13, 101.48, 49.37.

HRMS (ESI$^+$) calcd. for $C_{20}H_{16}NO_2$ [M+H]$^+$: 302.1181; found: 302.1171. **HRMS** (ESI$^-$) calcd. for $C_{20}H_{14}NO_2$ [M-H]$^-$: 300.1025; found: 300.1013.

1-(Naphthalen-1-ylmethyl)-1H-pyrrole-2-carboxylic acid (5a)

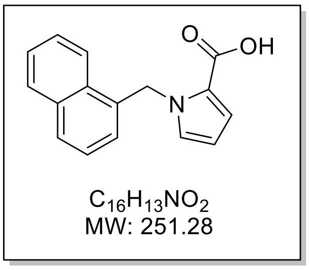

It was prepared from ethyl 1-(naphthalen-1-ylmethyl)-1H-pyrrole-2-carboxylate (108.90 mg, 0.39 mmol) and NaOH (1.5 M in water, 0.34 ml, 0.51 mmol) in ethanol (5 ml) according to General procedure 3. The precipitate was filtered off, washed with distilled water, and dried under reduced pressure overnight to obtain **5a** as white solid (61.2 mg, 61%): mp 160 °C.

^{1}**H NMR** (300 MHz, DMSO- d_6) δ 12.14 (br.s, 1H), 8.10 (d, J = 7.5 Hz, 1H), 7.99–7.94 (m, 1H), 7.83 (d, J = 8.2 Hz, 1H), 7.62–7.54 (m, 2H), 7.39 (t, J = 7.7 Hz, 1H), 7.12 (s, 1H), 6.96 (s, 1H), 6.54 (d, J = 7.0 Hz, 1H), 6.20 (s, 1H), 6.08 (s, 2H).

^{13}**C NMR** (75 MHz, DMSO-d_6) δ 161.84, 135.28, 133.02, 130.01, 129.74, 128.53, 127.38, 126.41, 125.98, 125.61, 122.88, 122.69, 117.88, 108.19, 49.09.

HRMS (ESI$^+$) calcd. for $C_{16}H_{14}NO_2$ [M+H]$^+$: 252.1025; found: 252.1018. **HRMS** (ESI$^-$) calcd. for $C_{16}H_{12}NO_2$ [M-H]$^-$: 250.0868; found: 250.0830.

1-(Naphthalen-2-ylmethyl)-1H-pyrrole-2-carboxylic acid (5b)

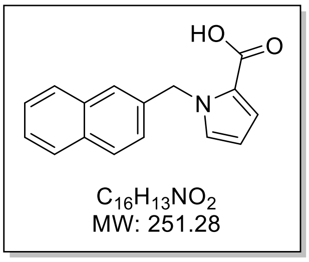

It was synthesized from ethyl 1-(naphthalen-2-ylmethyl)-1H-pyrrole-2-carboxylate (170.00 mg, 0.61 mmol) and NaOH (1.5 M in water, 0.65 ml, 0.97 mmol) in ethanol (5 ml) by General procedure 3. The precipitate was filtered off, washed with distilled water, and dried under reduced pressure overnight to obtain **5b** as a white solid (128.00 mg, 82%): mp 160 °C.

^{1}**H NMR** (300 MHz, DMSO-d_6) δ 12.15 (br.s, 1H), 7.86 (dd, J = 8.9, 4.1 Hz, 2H), 7.82–7.78 (m, 1H), 7.52 (s, 1H), 7.50–7.46 (m, 2H), 7.31–7.26 (m, 2H), 6.90 (dd, J = 3.9, 1.8 Hz, 1H), 6.18 (dd, J = 3.9, 2.6 Hz, 1H), 5.73 (s, 2H).

^{13}**C NMR** (75 MHz, DMSO-d_6) δ 161.83, 136.79, 132.79, 132.15, 129.61, 127.98, 127.56, 127.50, 126.26, 125.83, 125.04, 124.92, 122.10, 117.96, 108.11, 51.02.

HRMS (ESI$^+$) calcd. for $C_{16}H_{14}NO_2$ [M+H]$^+$: 252.1025; found: 252.1019. **HRMS** (ESI$^-$) calcd. for $C_{16}H_{12}NO_2$ [M-H]$^-$: 250.0868; found: 250.0836.

Table S1. Results of biochemical assay of bCSE and hCSE

Compound	IC_{50}* biochemical assay	
	bCSE, μM	hCSE, μM
**1a**	>500	>500
**1b**	>500	>500
**1c**	>500	>500
**2a**	64.6 ± 3.1	251.5 ± 16.6
**2b**	71.1 ± 4.2	284.8 ± 14.6
**3a**	79.3 ± 5.9	471.9 ± 37.3
**3b**	148.1 ± 46.4	>500
**4a**	138.2 ± 9.0	>500
**4b**	109.8 ± 11.7	>500
**5a**	289.3 ± 30.4	>500
**5b**	>500	>500
**NL2**	143.7 ± 9.0	>500
**PAG**	24.3 ± 0.7	0.17 ± 0.02

*Data are the mean ± SD of at least three independent measurements.

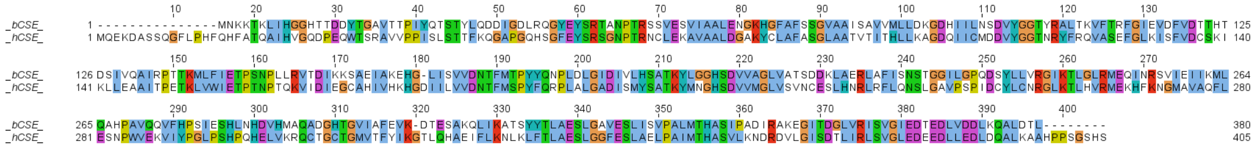

Figure S1. An alignment of amino acid sequences of bCSE (A0A2Y2FJW5) and hCSE (P32929) using Clustal Omega at EMBL-EBI [23].

Table S2. Susceptibility of the investigated isolates to the main antimicrobial agents.

Isolate	Sensitive to	Resistant to
A. baumannii GIMC5509:ABT-52Ts19	Polymyxin	Aztreonam, amikacin, ampiside (ampicillin + sulbactam), vancomycin, gentamicin, zavicefta (ceftazidim + avibactam), maxicam (cefepim + sulbactam), meropenem, moxifloxacin, ofloxacin, sulperazone (cefoperazone + sulbactam), tigecycline, cefepime, ceftazidime, ciprofloxacin, fosfomycin, ertapenem
P. aeruginosa GIMC5016:PA1840/36/2015	Colistin	Amikacin, gentamicin, imipenem, pilastatin levofloxacin meropenem, piperacillin, tobramycin, cefazolin, cefepime, cefoperazone/, sulbactam, cefotaxime, ceftazidime, ceftriaxone, ciprofloxacin

Table S3. Results of the microbiological assay of potentiating activity in combination with antibiotics against *S. aureus* ATCC 25923 and *S. aureus* INA00761 (MRSA)

Bacterial strain	*S. aureus* ATCC 25923			*S. aureus* INA00761		
Antibiotic	km	amp	nor	km	amp	nor
MIC (μg/ml)	12.5	0.3	2.5	res*	res	10
Range (μg/ml)	50-0.4	1.25-0.01	10-0.08	100-0.8	100-0.8	40-0.3
1a	12.5	0.3	1.25	res	res	10
1b	1.5	0.3	1.25	res	res	10
1c	12.5	0.3	2.5	res	res	10
**2a**	**0.3**	**0.07**	**0.08**	**res**	**0.8**	**10**
2b	0.4	0.15	0.3	res	res	10
3a	1.5	0.3	0.6	res	res	10
3b	1.5	0.3	0.8	res	res	10
4a	1.5	0.3	1.25	res	res	10
4b	0.7	0.3	0.3	res	res	10
5a	3	0.3	1.25	res	res	10
5b	3	0.3	2.5	res	res	10
NL2	0.7	0.02	0.07	0.7	0.8	0.3

*resistant to any tested concentrations of the antibiotic below the maximum of tested concentrations.

Table S4. Results of the microbiological assay of potentiating activity in combination with antibiotics against clinical isolates of *A. baumannii* and *P. aeruginosa.*

Clinical isolates	MIC (μg/ml)		**2a**	**NL2**
A. baumannii GIMC5509:ABT-52Ts19	Cefepim	>512	256	128
	Gentamicin	>512	>512	>512
	Meropenem	64	32	32
	Tacillin	256	64	128
P. aeruginosa GIMC5016:PA1840/36/2015	Cefepim	256	32	64
	Gentamicin	>512	>512	>512
	Meropenem	16	8	8
	Tacillin	64	32	32

Table S5. Solubility in 100 mM phosphate buffer pH 7.4

**Compound**	**Solubility (μM)**	**Comment**
Nicardipine	5.16±0.23	Negative control (low solubility)
Diclofenac sodium	>100	Positive control (high solubility)
**2a**	>100	-

Data are shown as mean ± SD (n = 3)

Table S6. Stability in human plasma

**Compound**	**% Remaining at 4 h**	**Comment**
Propantheline	0.21±0.04	Negative control (unstable compound)
Verapamil	103.70±8.29	Positive control (stable compound)
**2a**	97.58±7.95	-

Data are shown as mean ± SD (n = 3)

Table S7. Stability in SGF and SIF

Compound	% Remaining at 2 h		Comment
	SGF	SIF	
Omeprazole	< 0.1	90.45±10.04	Negative control (unstable at low pH)
Verapamil	114.47±4.70	86.23±5.57	Positive control (stable compound)
**2a**	91.88±2.68	104.07±14.23	-

Data are shown as mean ± SD (n = 3)

Table S8. Log$D_{7.4}$

Compound	Measured log$D_{7.4}$	Predicted ACD/Labs log$D_{7.4}$	Comment
Paracetamol	0.29±0.02	0.40	Negative control (log$D_{7.4}$ < 1)
Ketoconazole	3.81±0.20	3.54	Positive control (log$D_{7.4}$ > 3)
**2a**	2.14±0.14	1.83	-

Data are shown as mean ± SD (n = 3)

Table S9. Caco-2 cell permeability

Compound	P_{app} A to B (10^{-6} cm/s)	P_{app} B to A (10^{-6} cm/s)	Efflux ratio	Comment
Atenolol	1.4±0.1	0.7±0.1	0.54	Negative control (low penetration ability)
Propranolol	30.2±2.9	31.6±1.1	1.04	Positive control (high penetration ability)
**2a**	43.3±2.8	49.1±6.0	1.13	-

Data are shown as mean ± SD (n = 2)

Table S10. Metabolic stability in rat liver microsomes

Compound	Cofactor	CL_{int} (µl/min/mg)	$t_{1/2}$ (min)	Comment
Verapamil	NADPH	70.20±0.52	19.75±0.15	Positive control (metabolized by CYP450)
Umbelliferone	NADPH	8.81±1.00	158.43±18.05	Negative control (metabolized by UGTs (uridine 5’-diphospho-glucuronosyltransferases))
**2a**	NADPH	29.22±0.29	47.44±0.47	-
Verapamil	UDPGA	<3	>500	Negative control (metabolized by CYP450)
Umbelliferone	UDPGA	180.02±0.70	7.70±0.03	Positive control (metabolized by UGTs)
**2a**	UDPGA	9.20±1.05	151.70±17.27	-

Data are shown as mean ± SD (n = 2)

Table S11. Plasma protein binding

**Compound**	**Fraction bound f_b (%)**	**Comment**
Atenolol	<5	Negative control (low binding)
Propranolol	85.81±2.42	Positive control (high binding)
**2a**	>99	-

Data are shown as mean ± SD (n = 2)

Table S12. HEK293 cytotoxicity assays.

**Compound**	**CC50, µM**	**Comment**
Doxorubicin	1.30 ± 0.71	Positive control (high toxicity)
**1a**	432.00 ± 37.51	-
**1b**	422.90 ± 71.78	-
**1c**	307.60 ± 75.50	-
**2a**	291.00 ± 8.11	-
**2b**	367.80 ± 32.83	-
**3a**	452.30 ± 21.51	-
**3b**	528.20 ± 47.10	-
**4a**	392.20 ± 21.84	-
**4b**	216.90 ± 93.20	-
**5a**	459.90 ± 51.08	-
**5b**	847.80 ± 72.15	-

Data are shown as mean ± SD (n = 3)

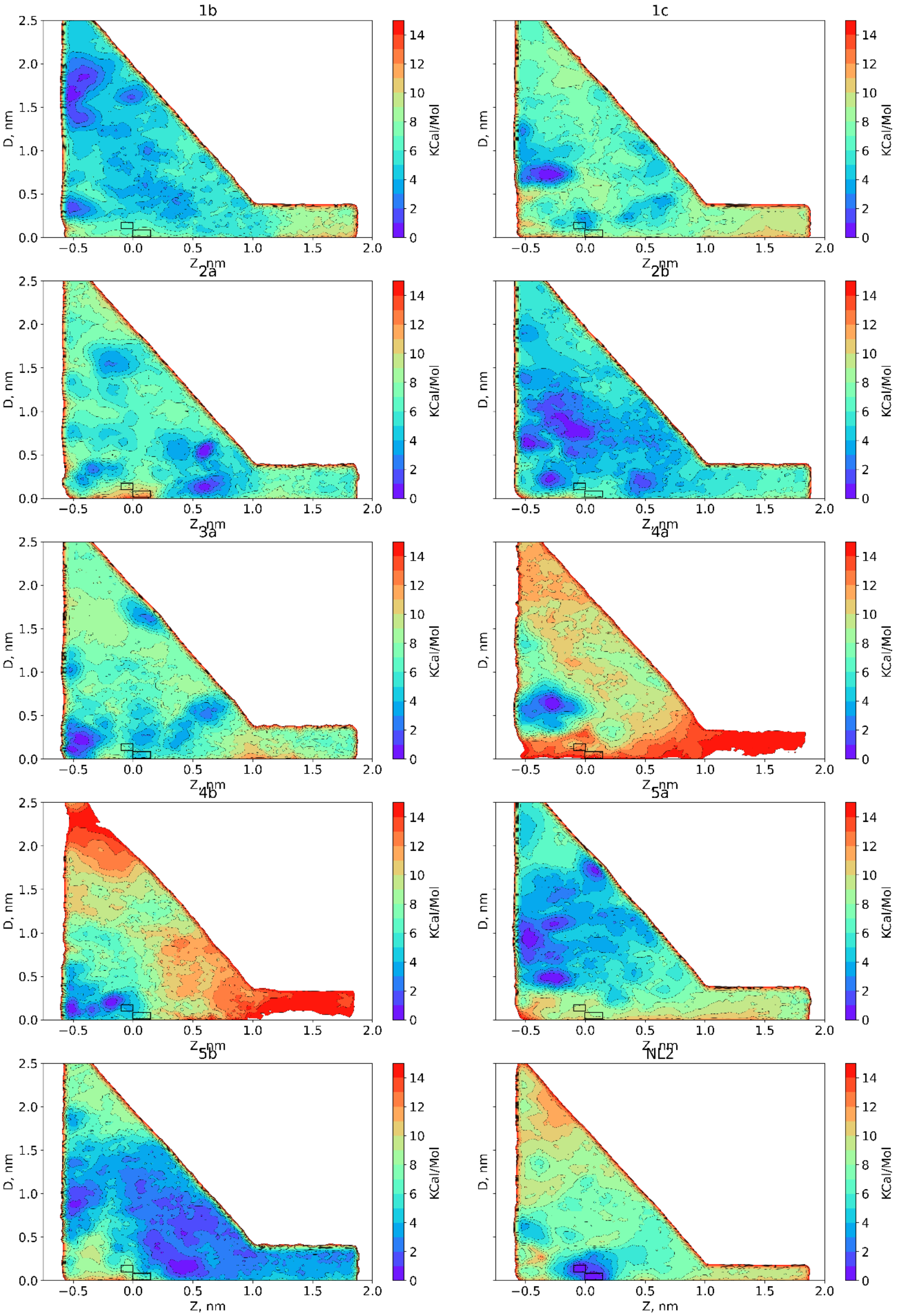

Figure S2. Potential energy surfaces obtained for compounds 1-5 from funnel metadynamics simulation data.

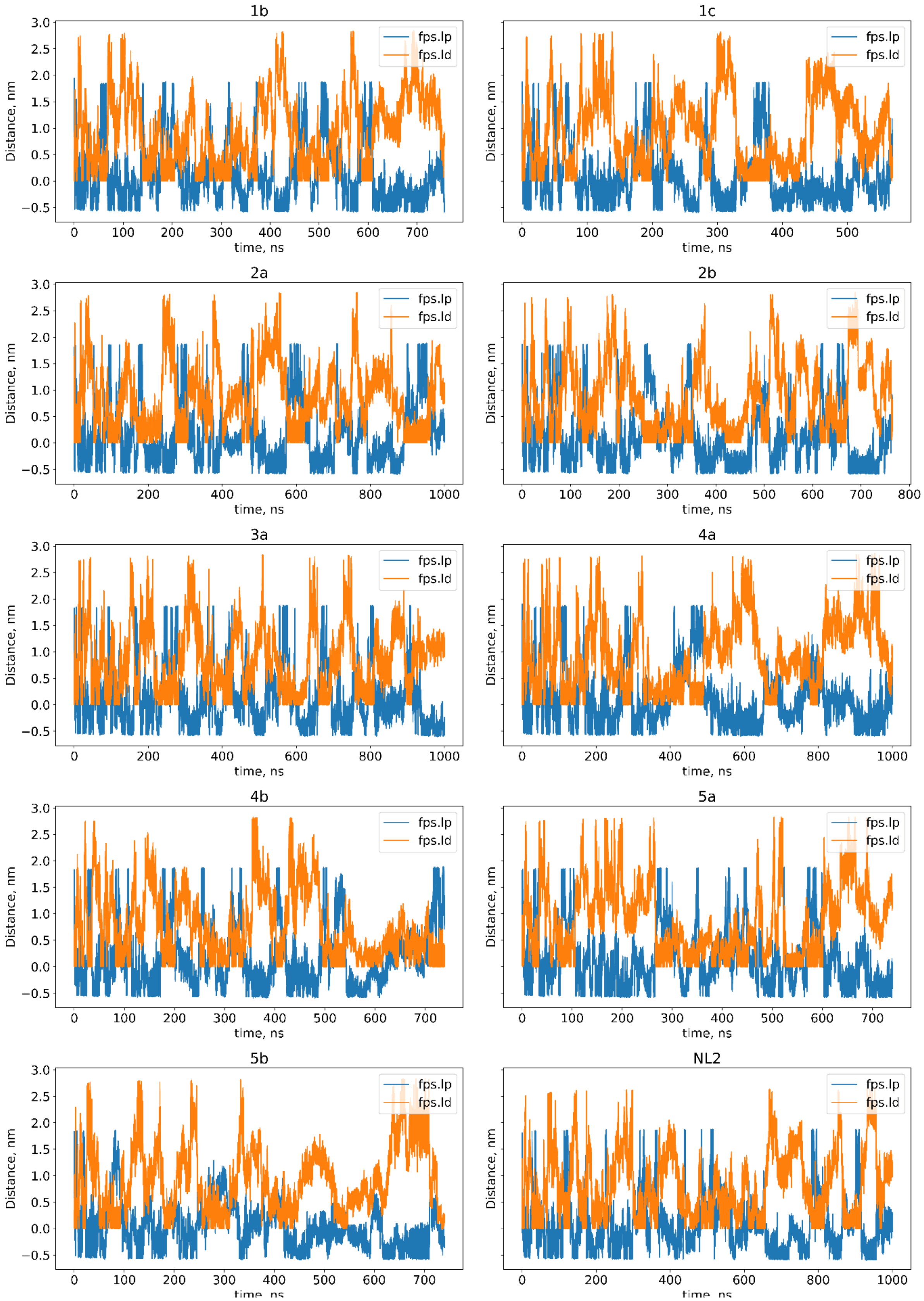

Figure S3. Dependence of the values of collective variables during the simulation of funnel metadynamics for compounds **1-5**. One change in fps.lp (the distance between COM **NL2** in the binding site of X-ray structure and the point solution 20 Å far from **NL2** position) from 0 to 2 nm reflects one act of dissociation and from 2 to 0 is binding event.

Table S13. Results of first stage docking calculations of molecules **1a-5a, 1b-5b, 1c.** Highlighting (according to enzymatic assays): green - most potent compounds inhibiting bCSE, yellow – medium active, orange – poorly active.

Compound	GlideScore, kcal/mol (monomer)	IC50 (uM)
NL2	-5.0	144
1a	-6.2	>500
2a	-4.2	65
3a	-5.7	79
4a	-5.8	138
5a	-5.2	289
1b	-6.3	>500
2b	-4.7	71
3b	-6.3	141
4b	-6.6	110
5b	-5.3	>500
1c	-6.1	>500

Table S14. Results of docking at two active sites, identified after MD simulations, in tetrameric bCSE. Highlighting: red – poorly active compounds, yellow – moderate activity, green – most potent molecules. Scoring function values are in correlation with the enzymatic activity.

Compound	$GlideScore_1$ (kcal/mol)	$GlideScore_2$ (kcal/mol)	IC50 (uM)
NL-2	-6.3	-7.3	144
1a	-4.7	-4.9	>500
2a	-6.6	-6.3	65
3a	-6.1	-6.4	79
4a	-6.3	-6.5	138
5a	-5.9	-6.0	289
1b	-5.3	-4.8	>500
2b	-6.0	-6.0	71
3b	-6.1	-6.7	141
4b	-6.8	-8.1	110
5b	-5.9	-5.3	>500
1c	-5.2	-5.5	>500

Table S15. Total MMGBSA ΔG energy for protein-ligand complexes in two sites of bCSE

Compound	ΔG_1 (kcal/mol)	ΔG_2 (kcal/mol)	IC50 (uM)
2a	-42.77	-41.21	65
3a	-40.36	-39.51	79
4a	-38.60	-43.11	138
5a	-30.43	-29.71	289
2b	-41.34	-37.90	71
3b	-57.84	-46.13	141
4b	-41.33	-55.51	110
5b	-38.28	-30.98	>500

1-(naphthalen-1-ylmethyl)-1H-indole-3-carboxylic acid (1a)

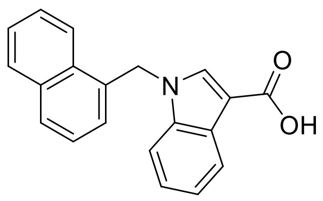

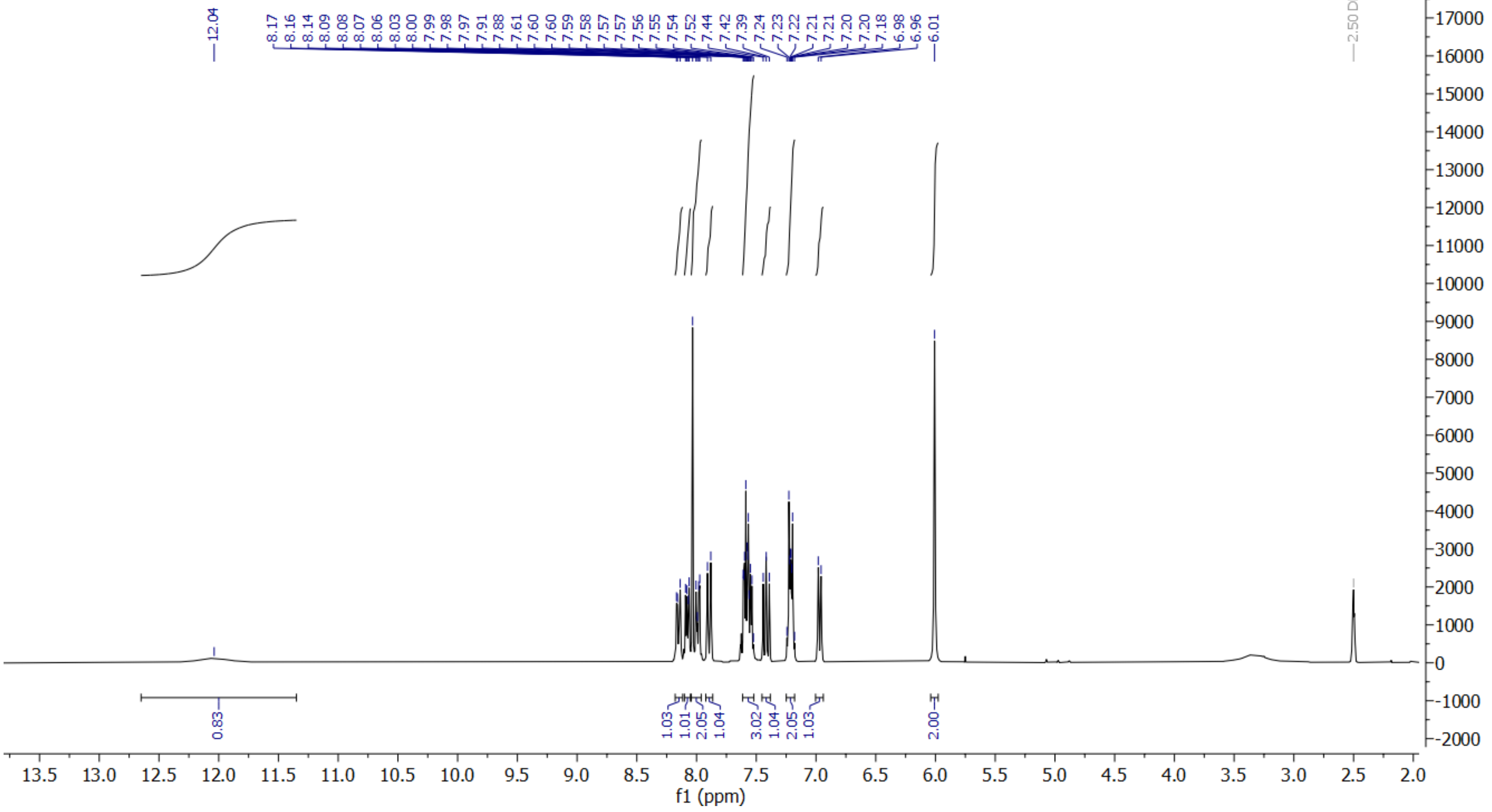

1-(naphthalen-1-ylmethyl)-1H-indole-3-carboxylic acid (1a)

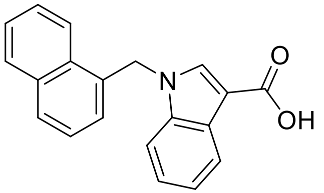

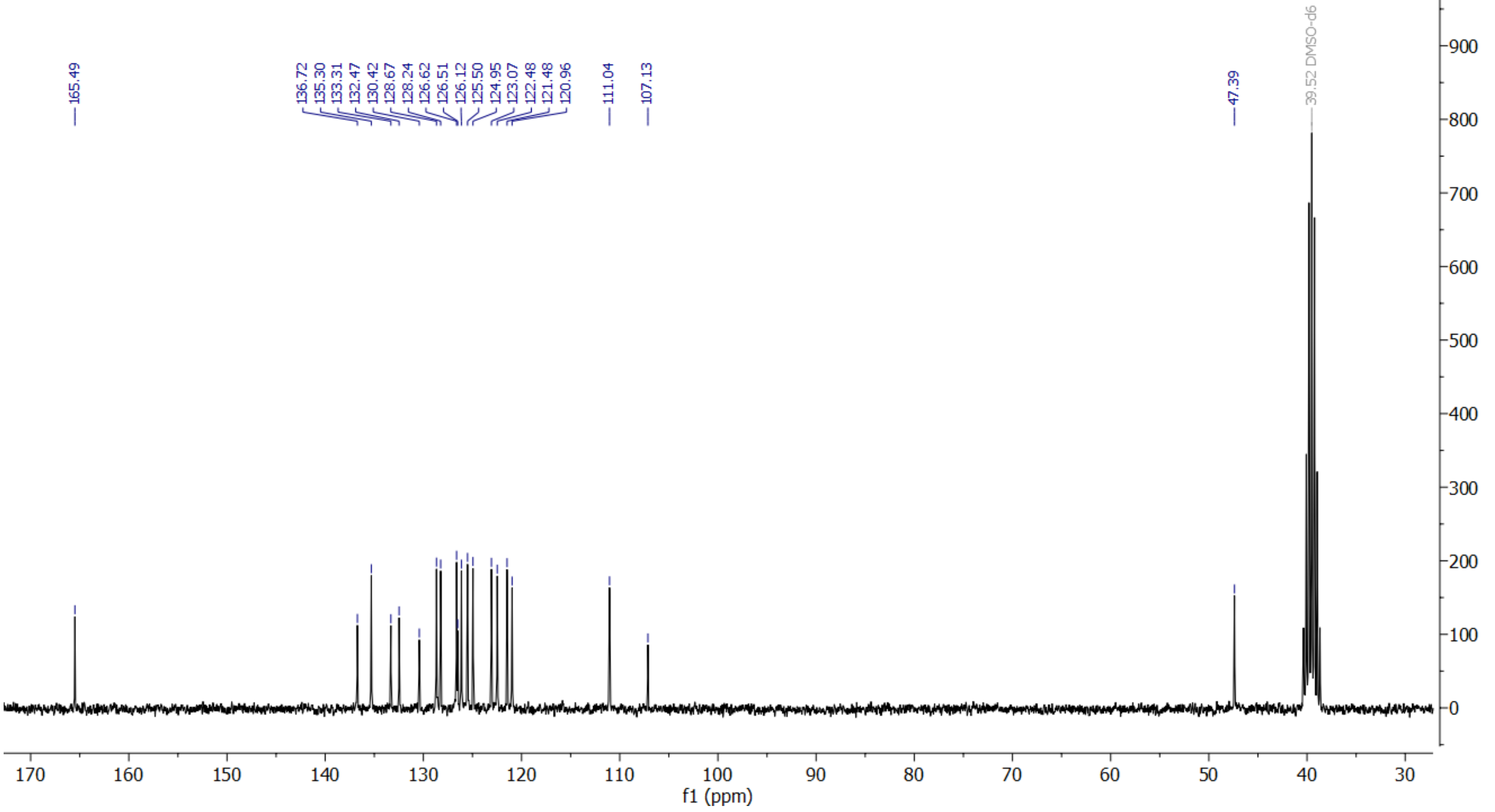

1-(naphthalen-2-ylmethyl)-1H-indole-3-carboxylic acid (1b)

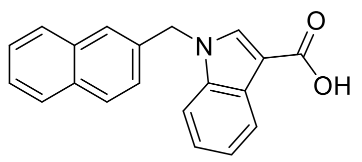

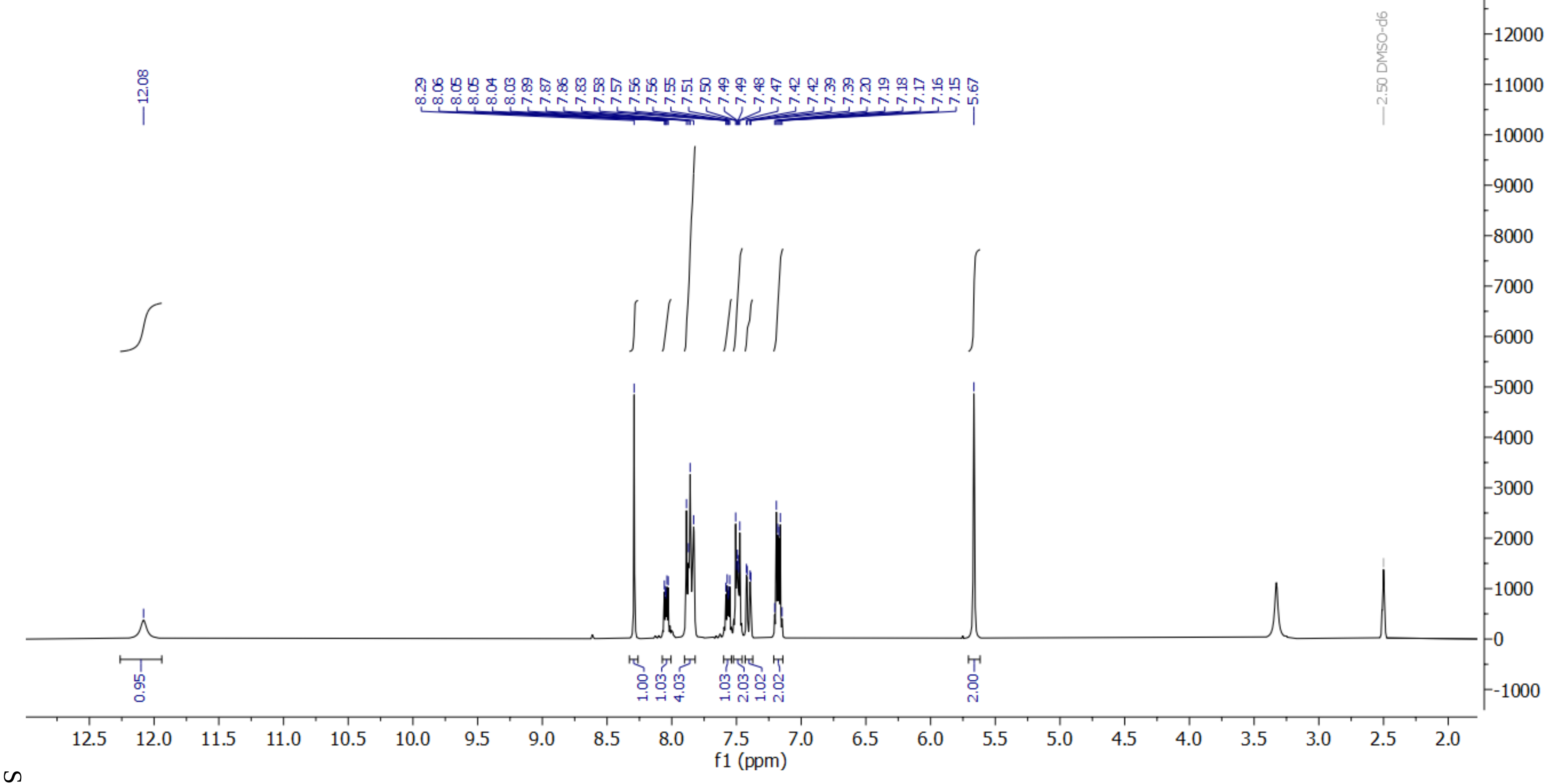

1-(naphthalen-2-ylmethyl)-1H-indole-3-carboxylic acid (1b)

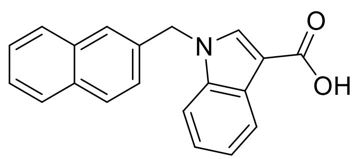

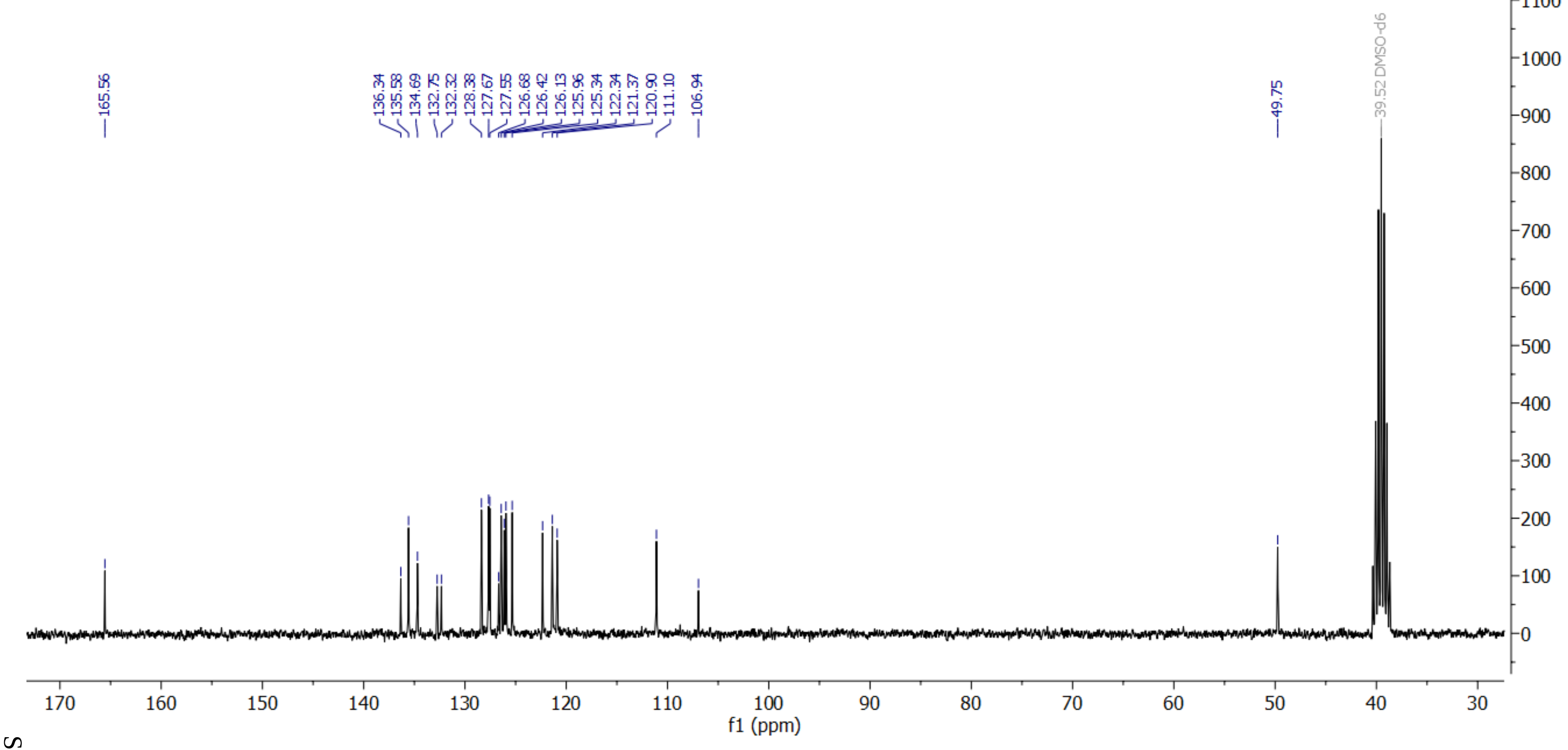

Sodium 1-(naphthalen-1-ylmethyl)-1H-indole-3-carboxylate (1c)

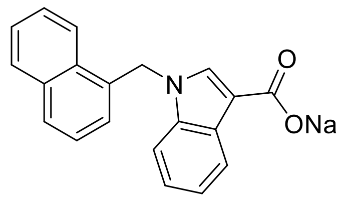

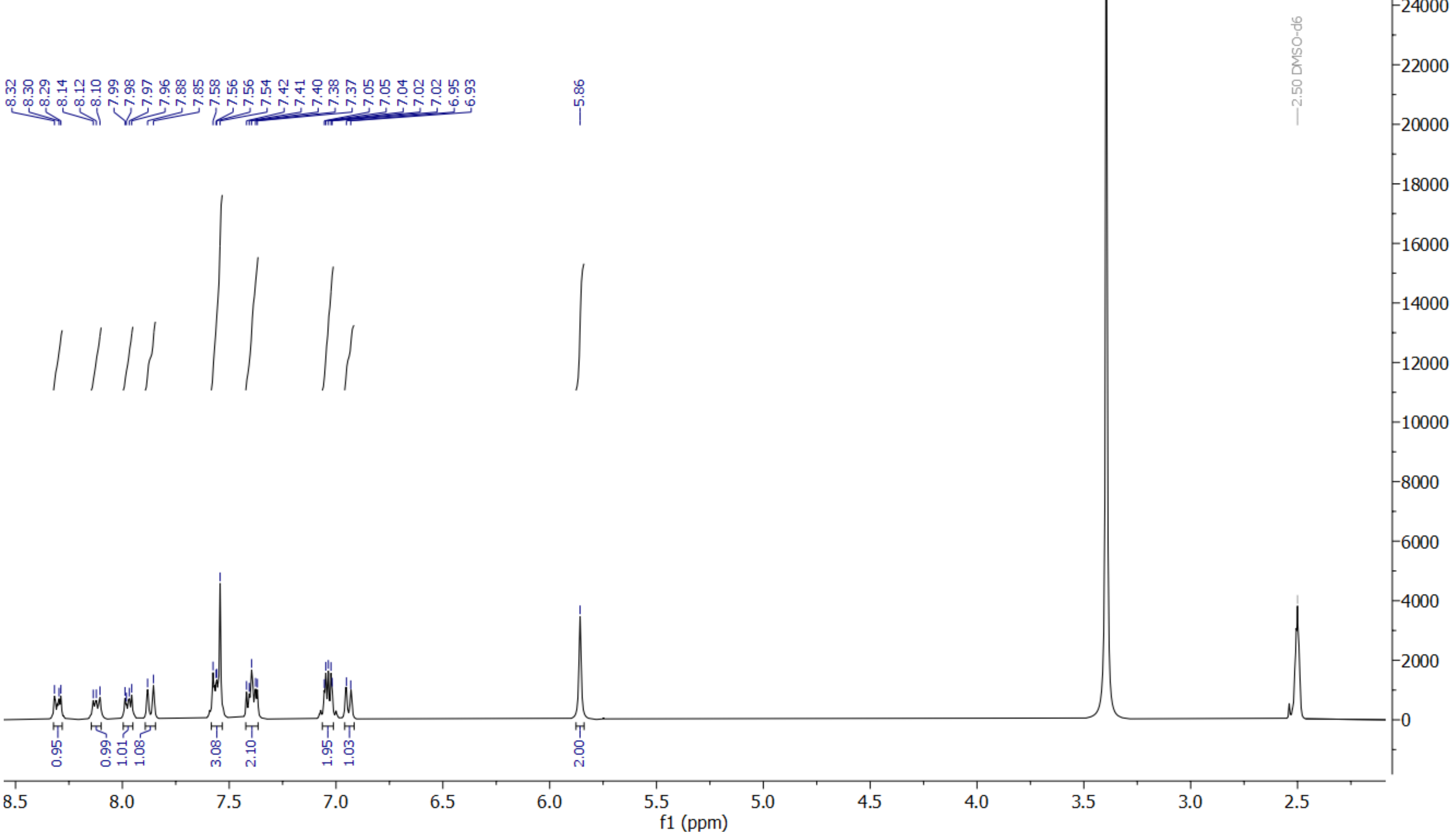

Sodium 1-(naphthalen-1-ylmethyl)-1H-indole-3-carboxylate (1c)

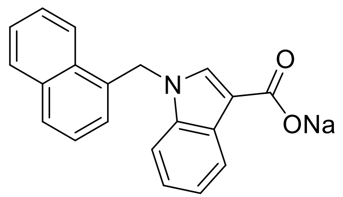

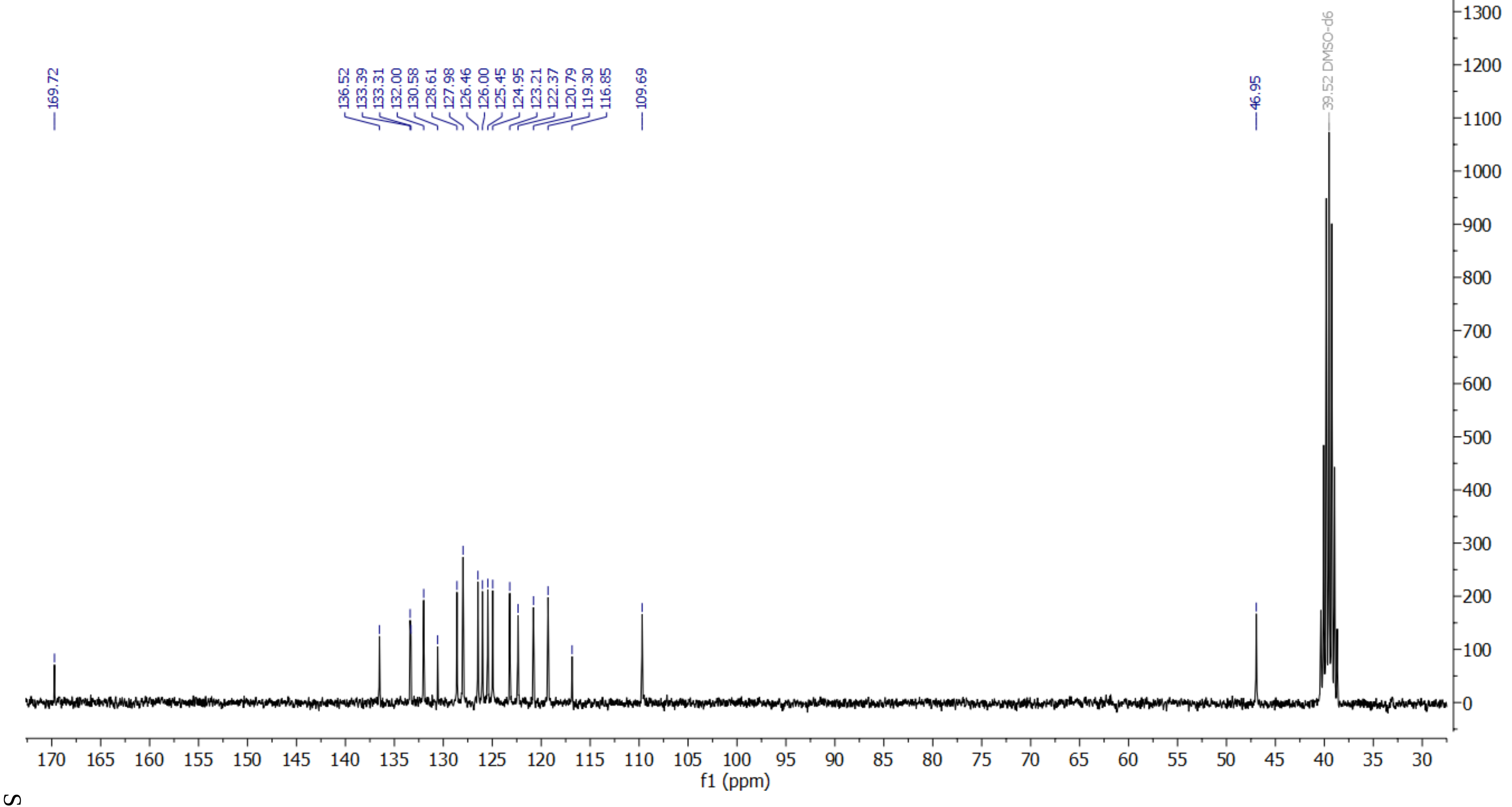

1-(naphthalen-1-ylmethyl)-1H-indole-5-carboxylic acid (3a)

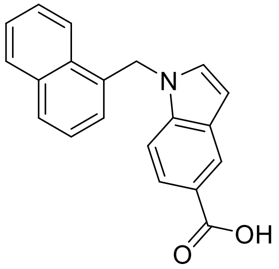

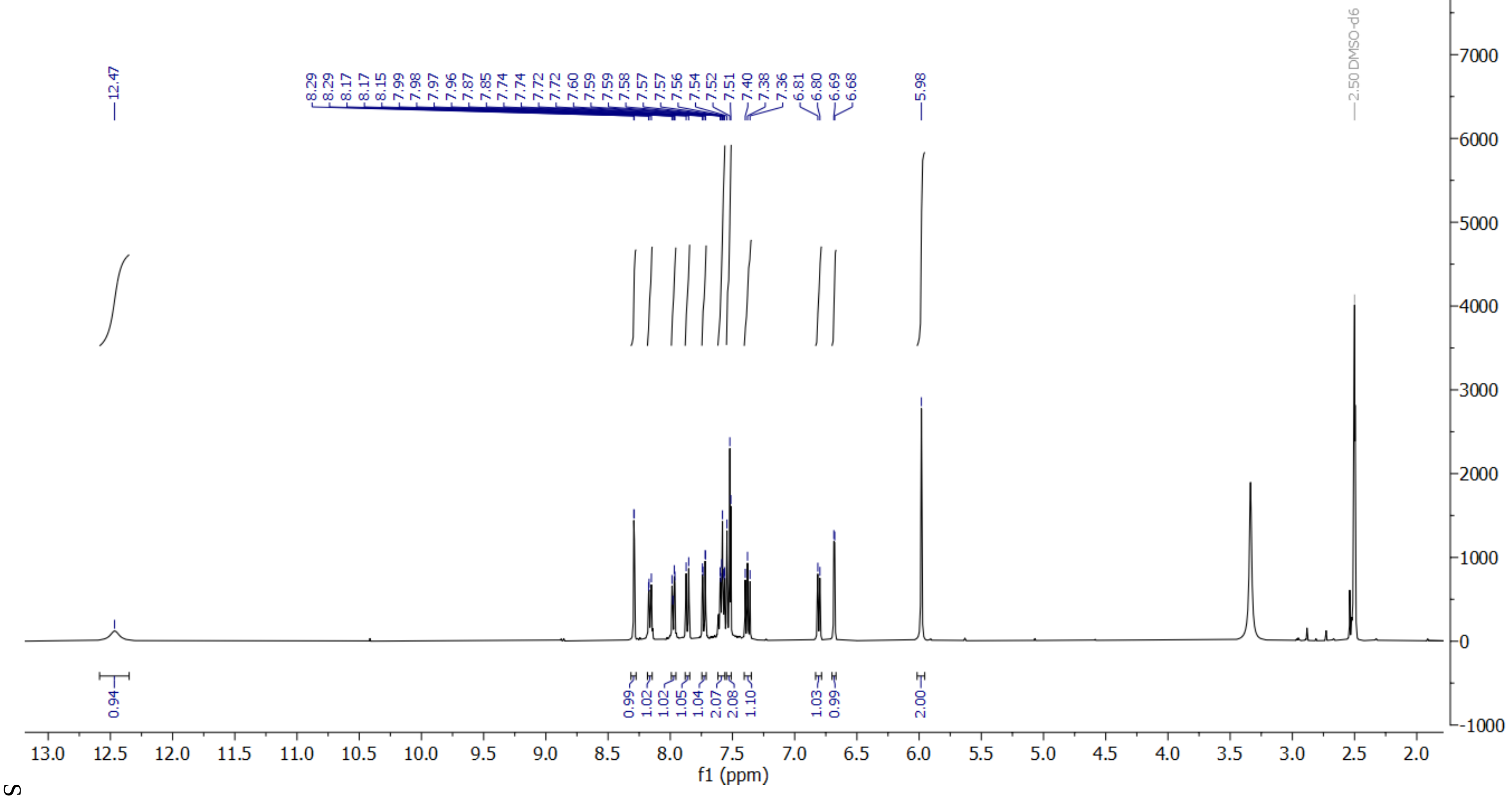

1-(naphthalen-1-ylmethyl)-1H-indole-5-carboxylic acid (3a)

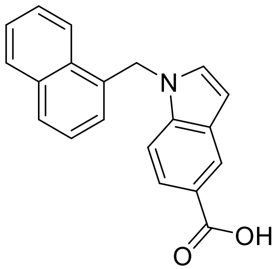

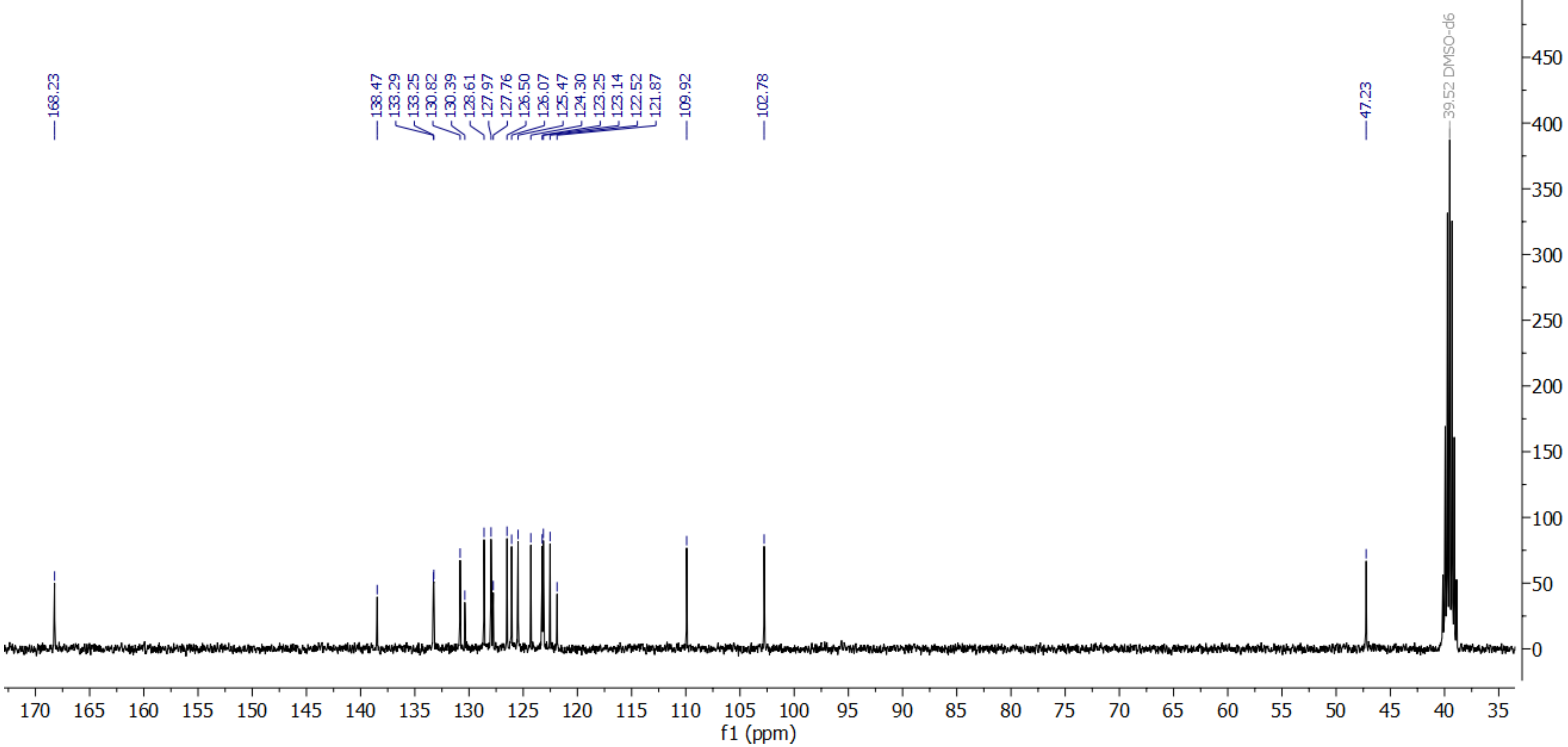

1-(naphthalen-1-ylmethyl)-1H-indole-6-carboxylic acid (4a)

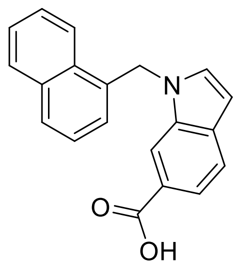

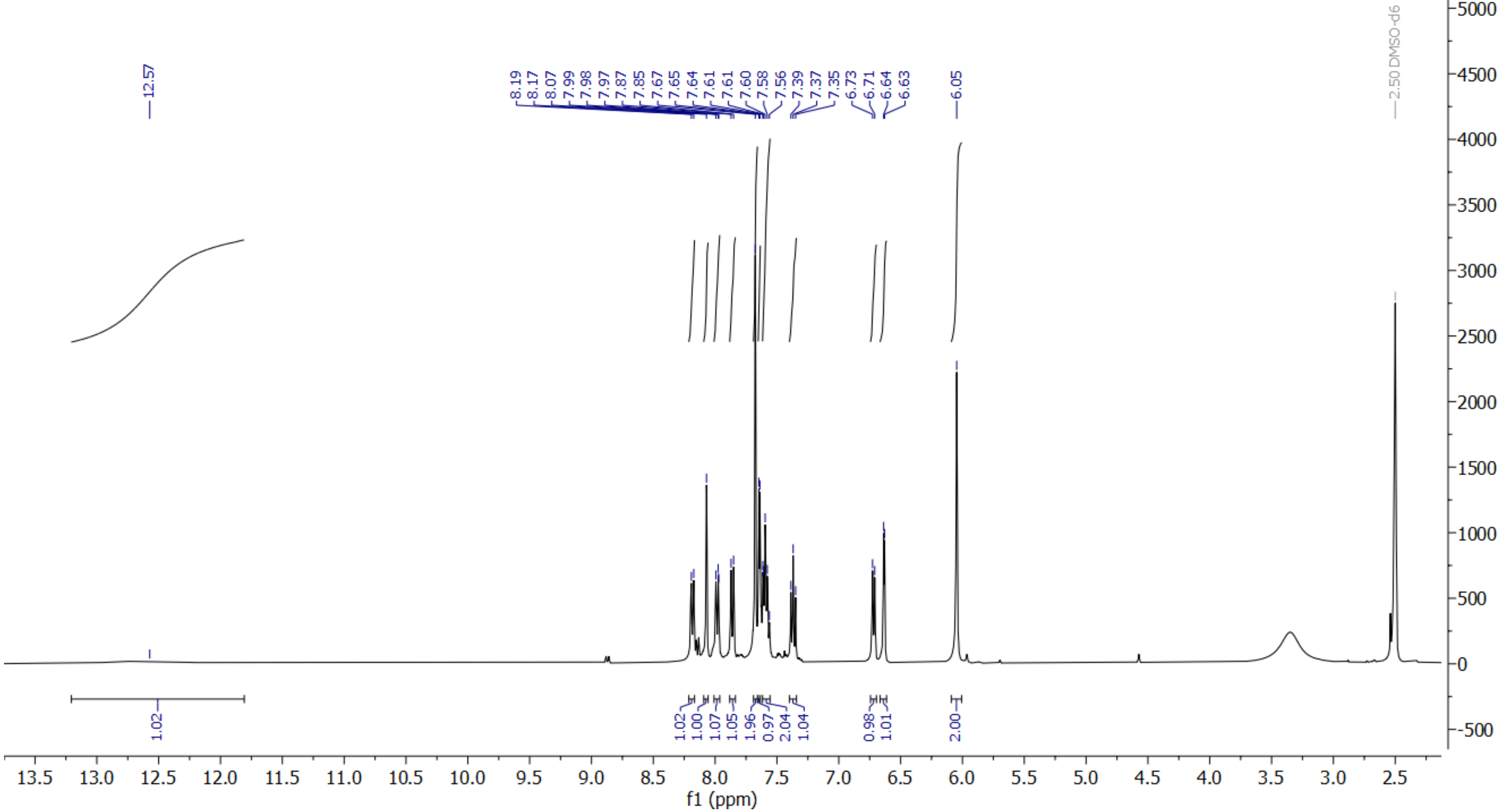

1-(naphthalen-1-ylmethyl)-1H-indole-6-carboxylic acid (4a)

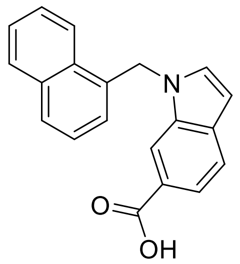

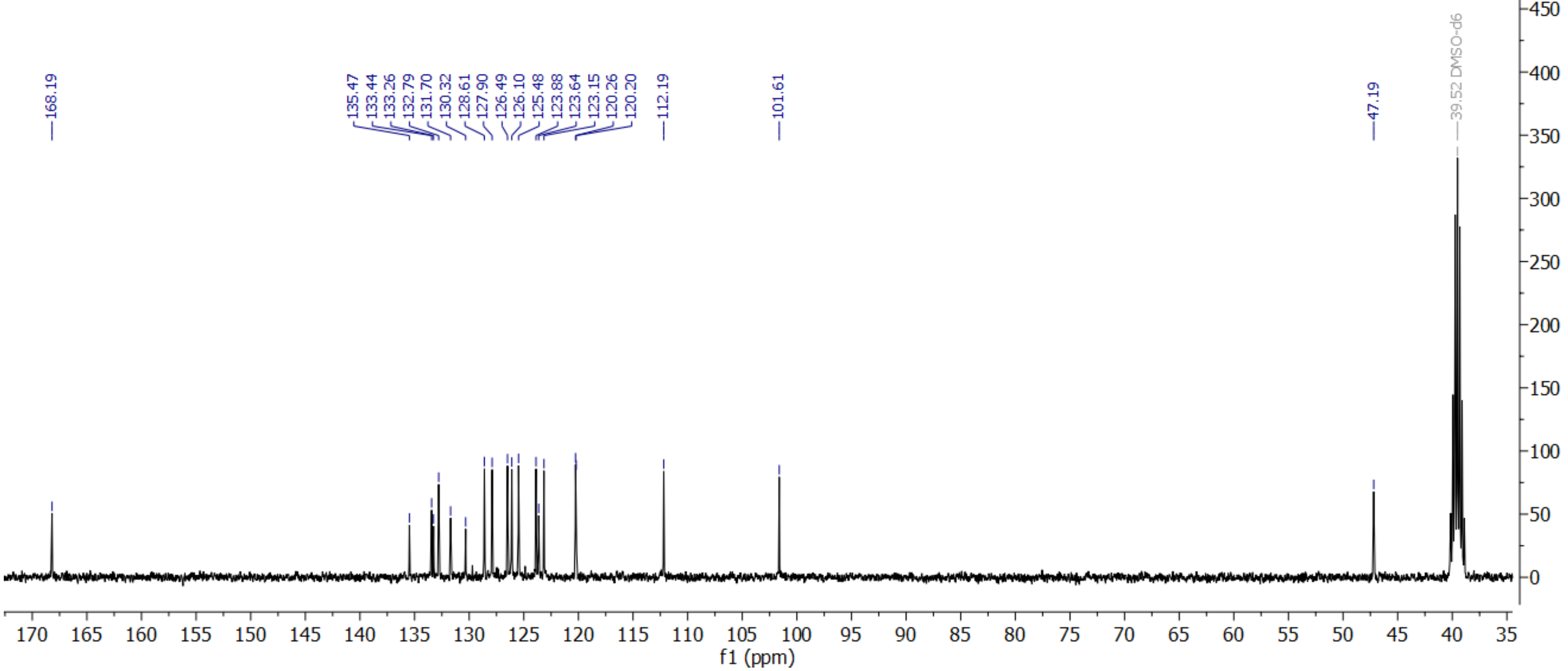

1-(naphthalen-2-ylmethyl)-1H-indole-5-carboxylic acid (4b)

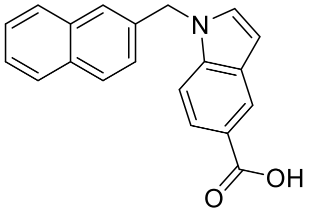

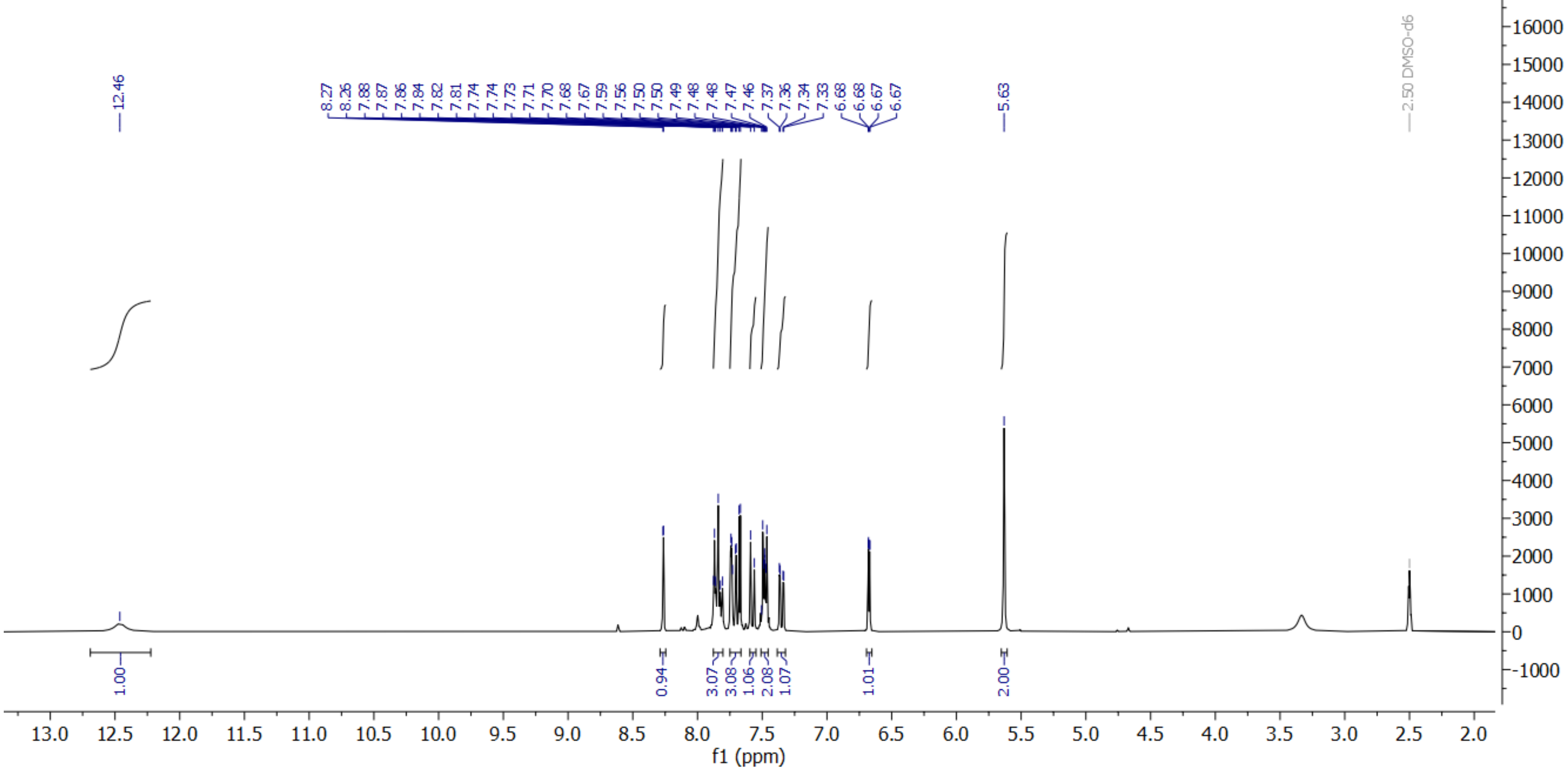

1-(naphthalen-2-ylmethyl)-1H-indole-5-carboxylic acid (4b)

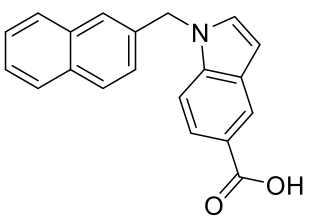

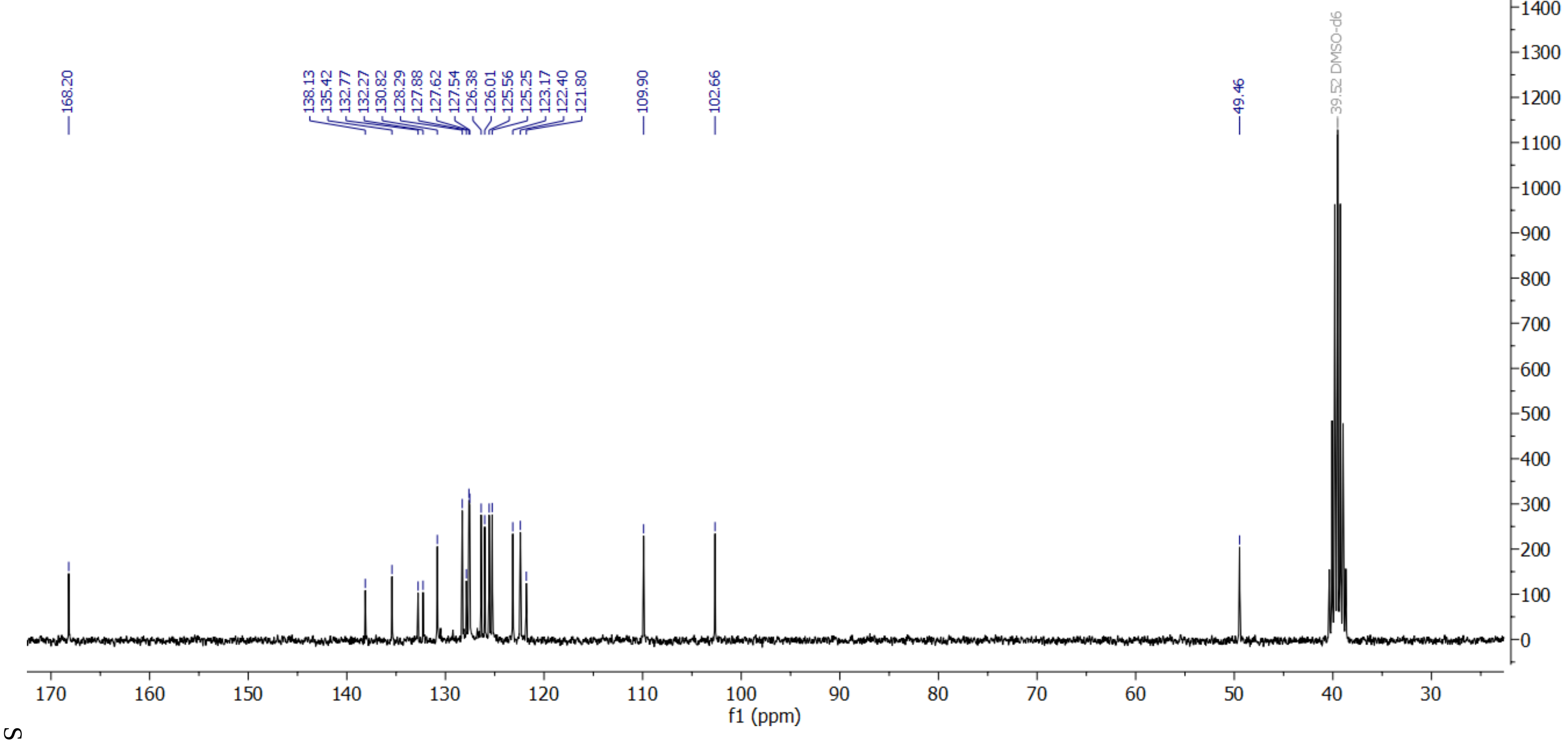

Ethyl 1-(naphthalen-1-ylmethyl)-1H-indole-2-carboxylate (2c)

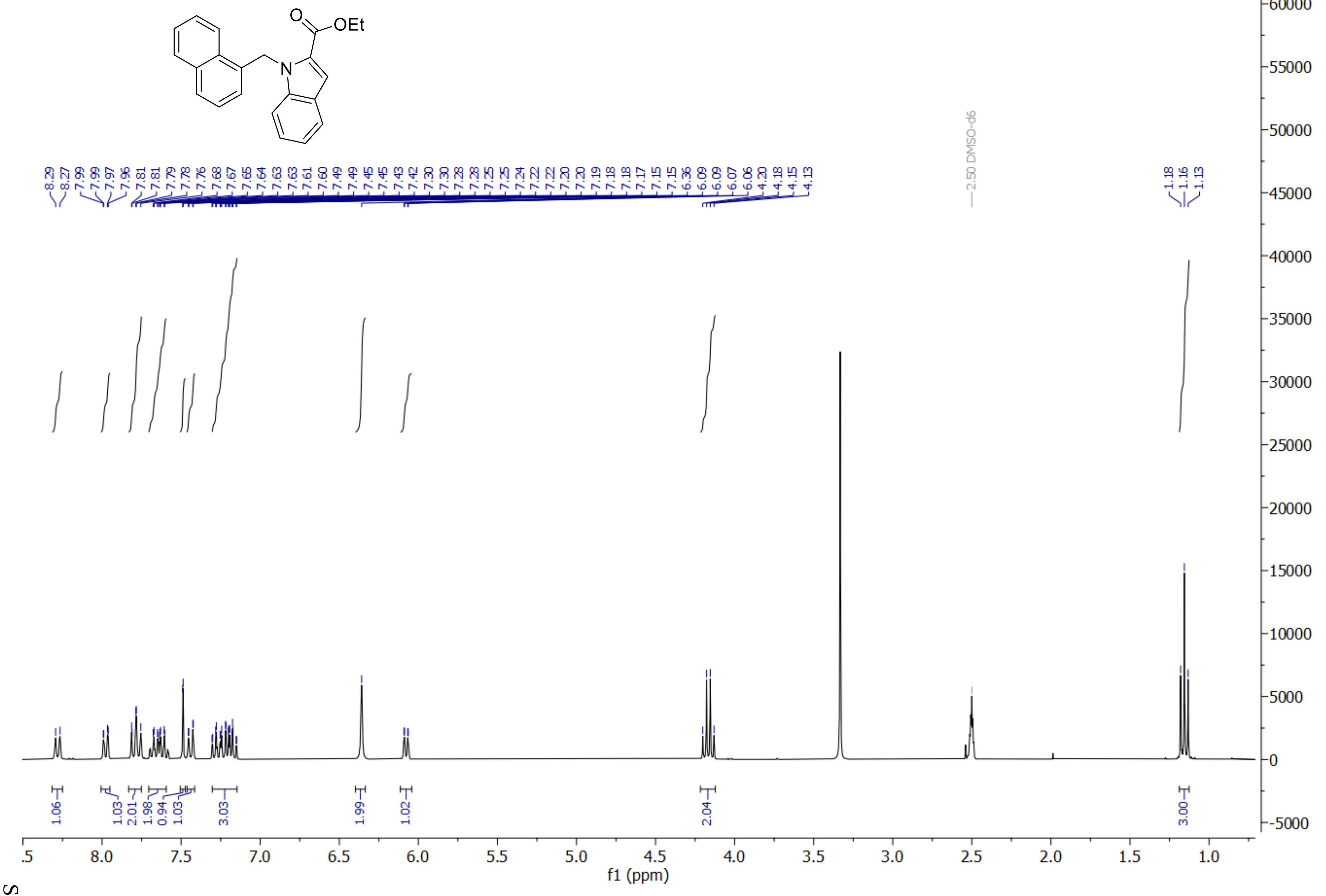

Ethyl 1-(naphthalen-1-ylmethyl)-1H-indole-2-carboxylate (2c)

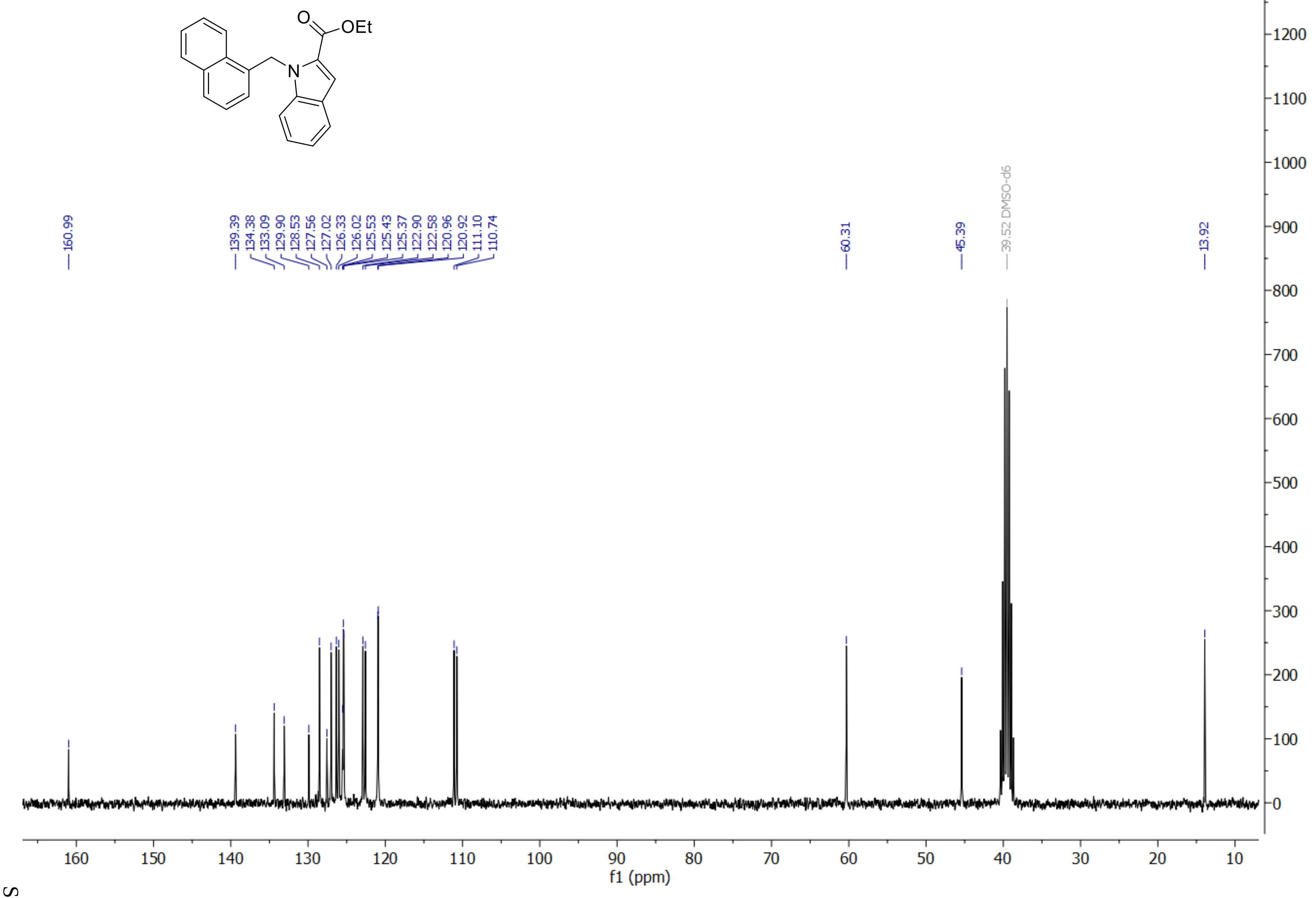

Ethyl 1-(naphthalen-2-ylmethyl)-1H-indole-2-carboxylate (2d)

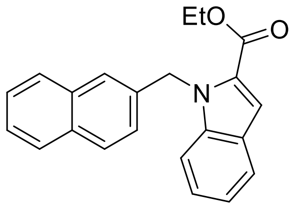

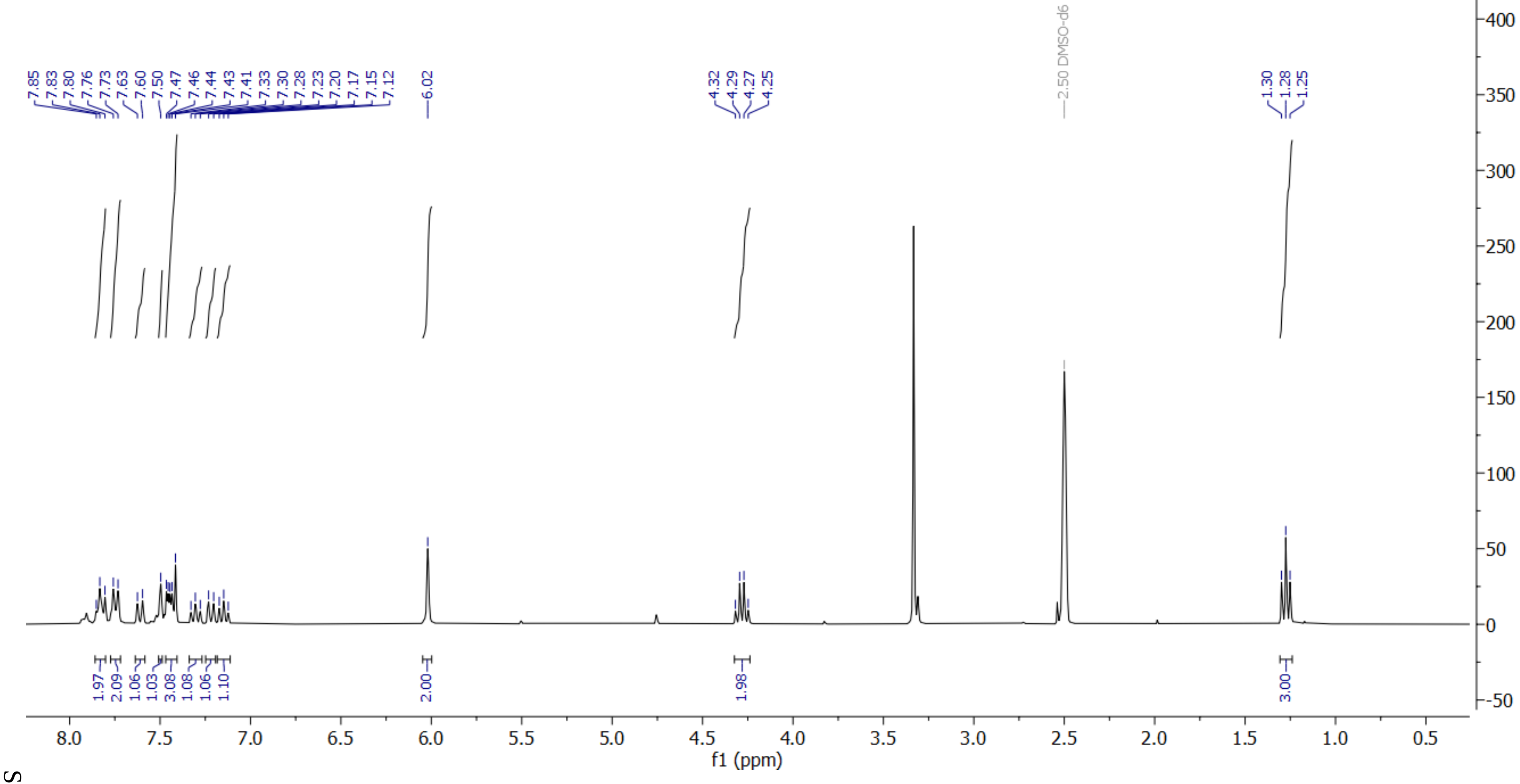

Ethyl 1-(naphthalen-2-ylmethyl)-1H-indole-2-carboxylate (2d)

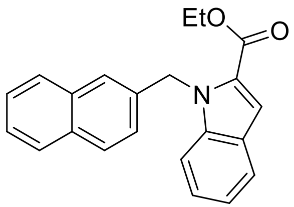

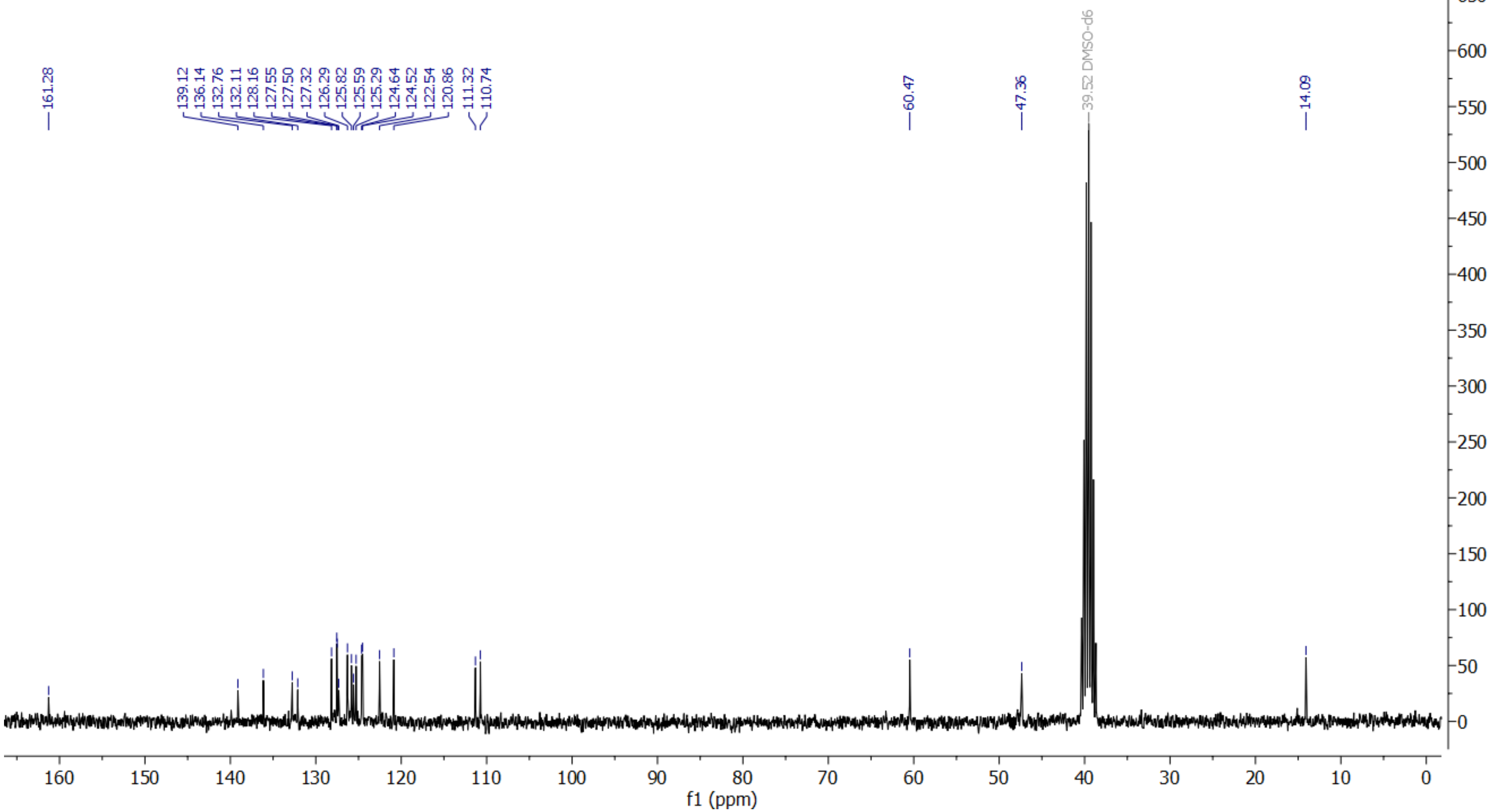

Methyl 1-(naphthalen-2-ylmethyl)-1H-indole-6-carboxylate (3c)

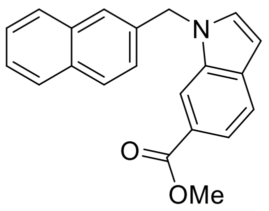

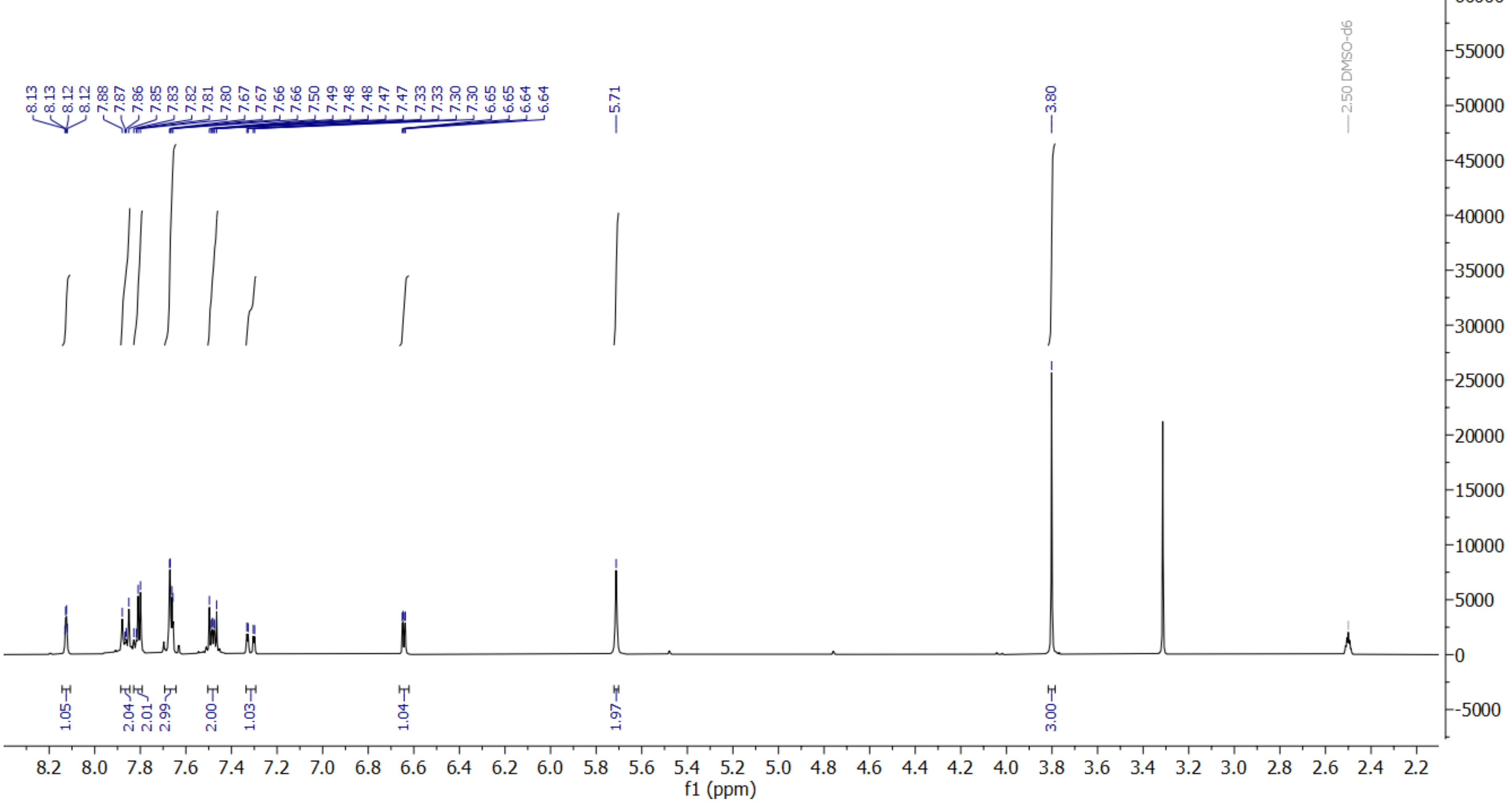

Methyl 1-(naphthalen-2-ylmethyl)-1H-indole-6-carboxylate (3c)

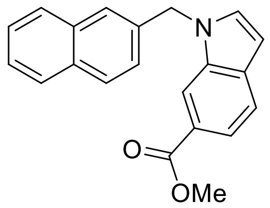

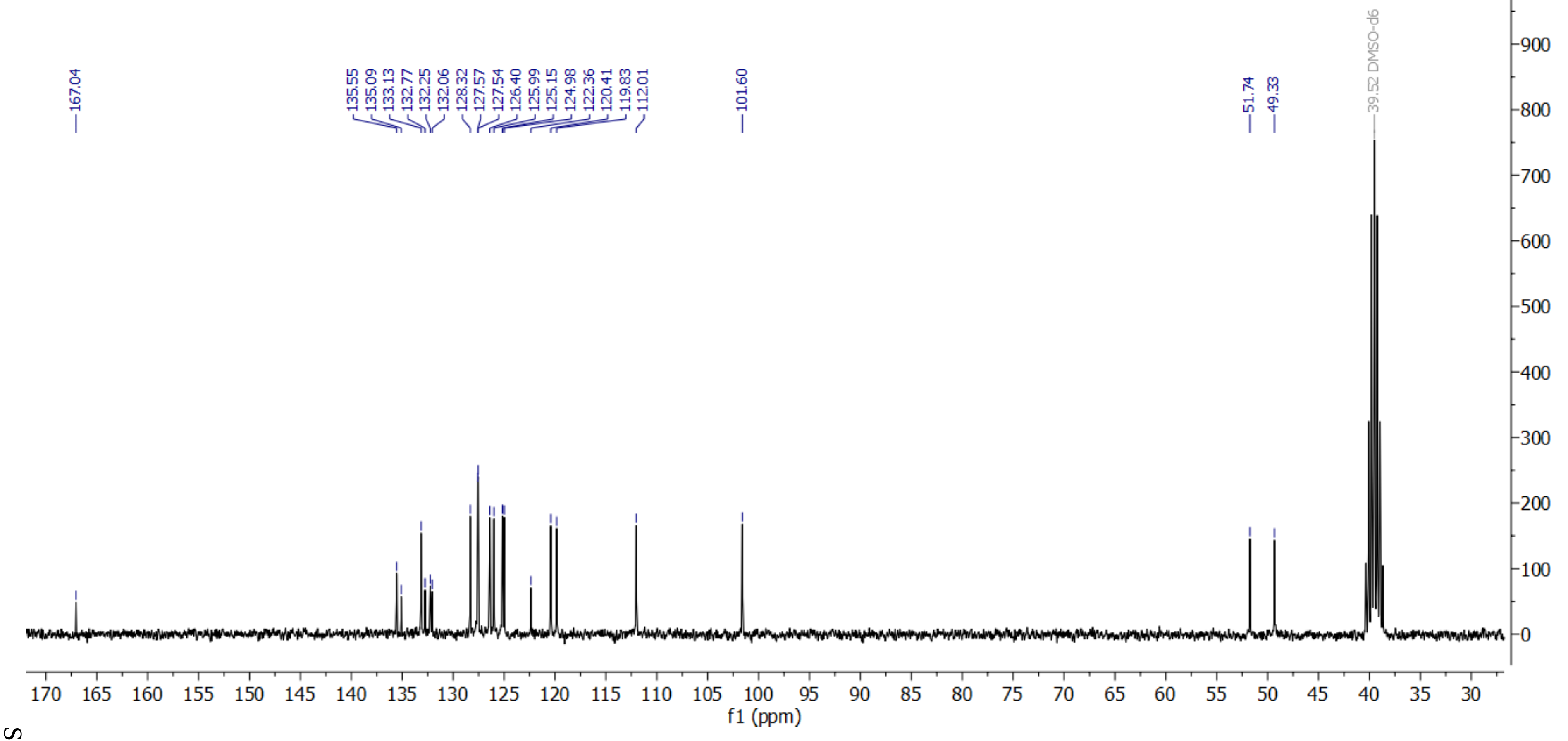

Ethyl 1-(naphthalen-1-ylmethyl)-1H-pyrrole-2-carboxylate (5c)

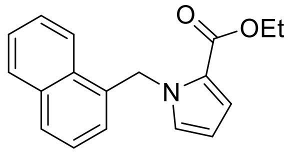

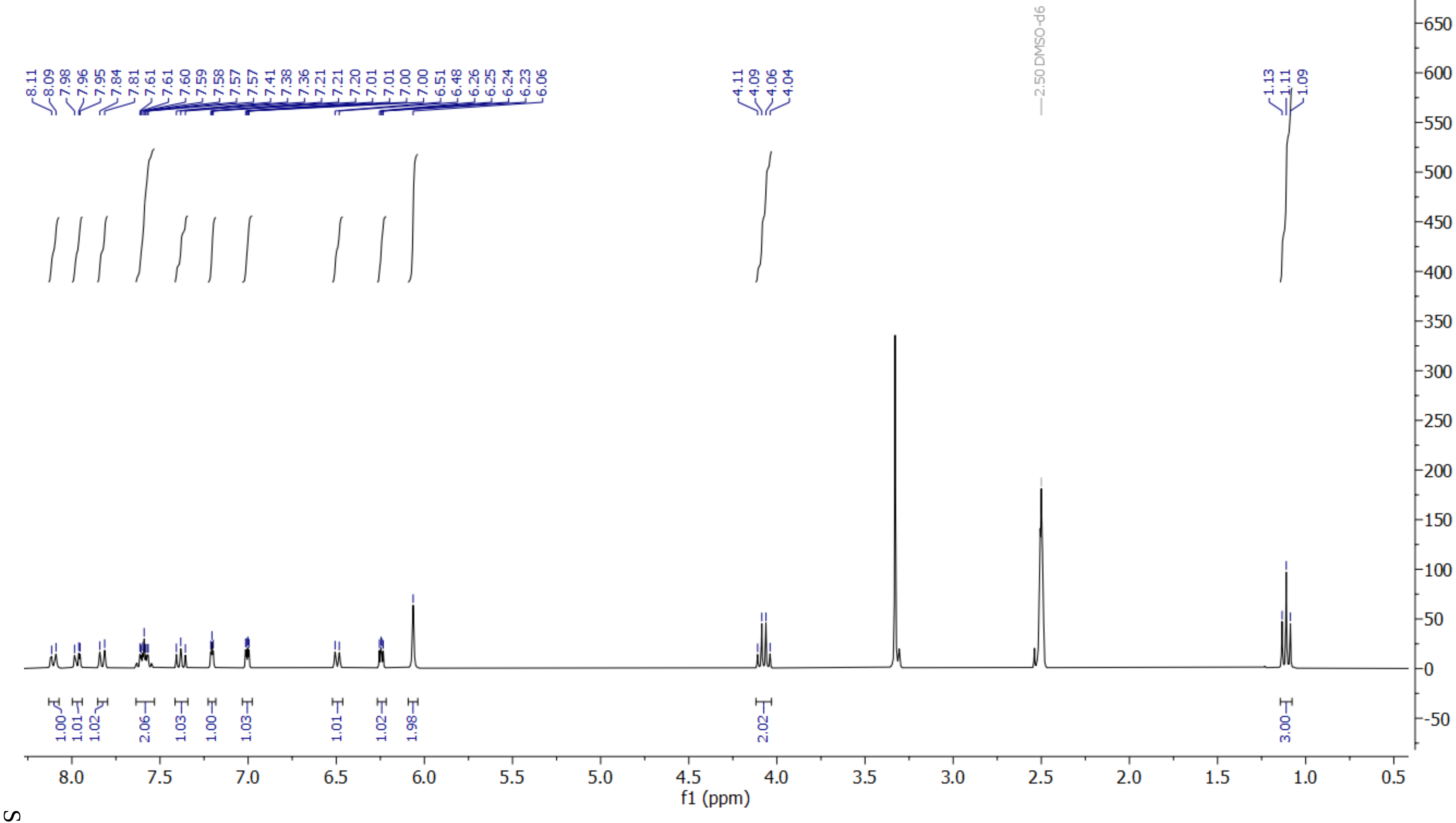

Ethyl 1-(naphthalen-1-ylmethyl)-1H-pyrrole-2-carboxylate (5c)

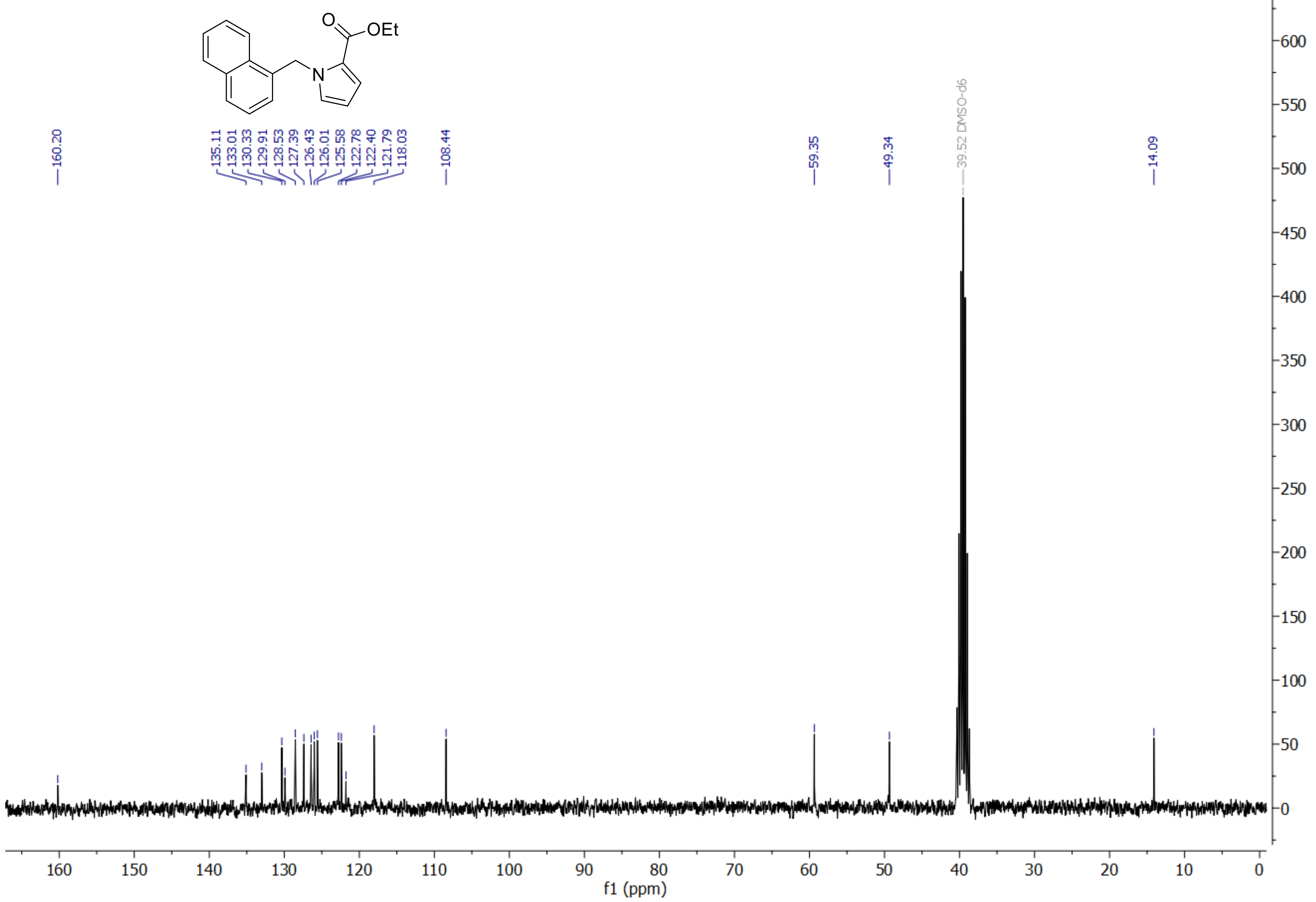

Ethyl 1-(naphthalen-2-ylmethyl)-1H-pyrrole-2-carboxylate (5d)

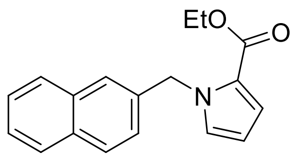

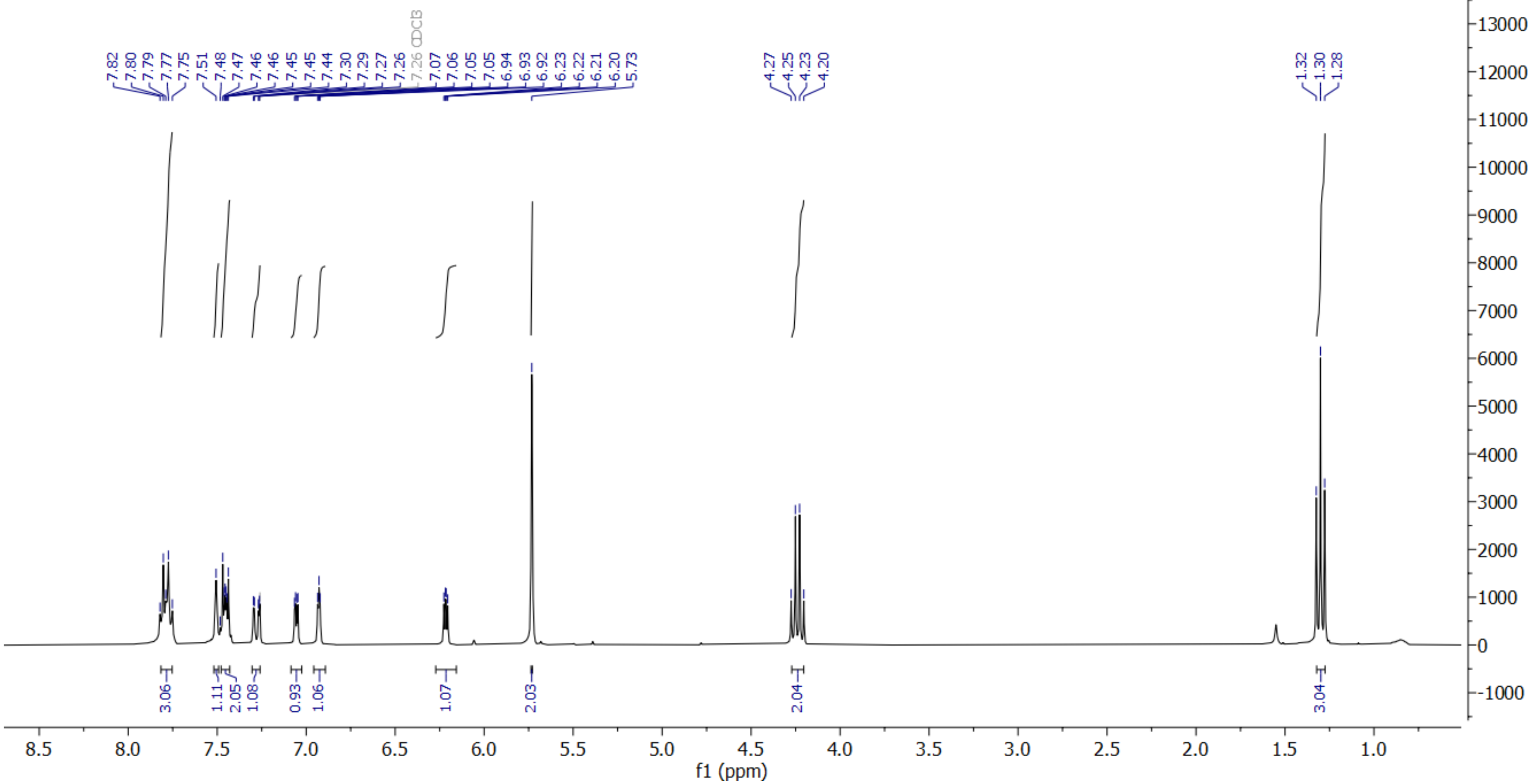

Ethyl 1-(naphthalen-2-ylmethyl)-1H-pyrrole-2-carboxylate (5d)

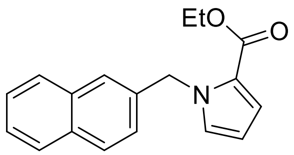

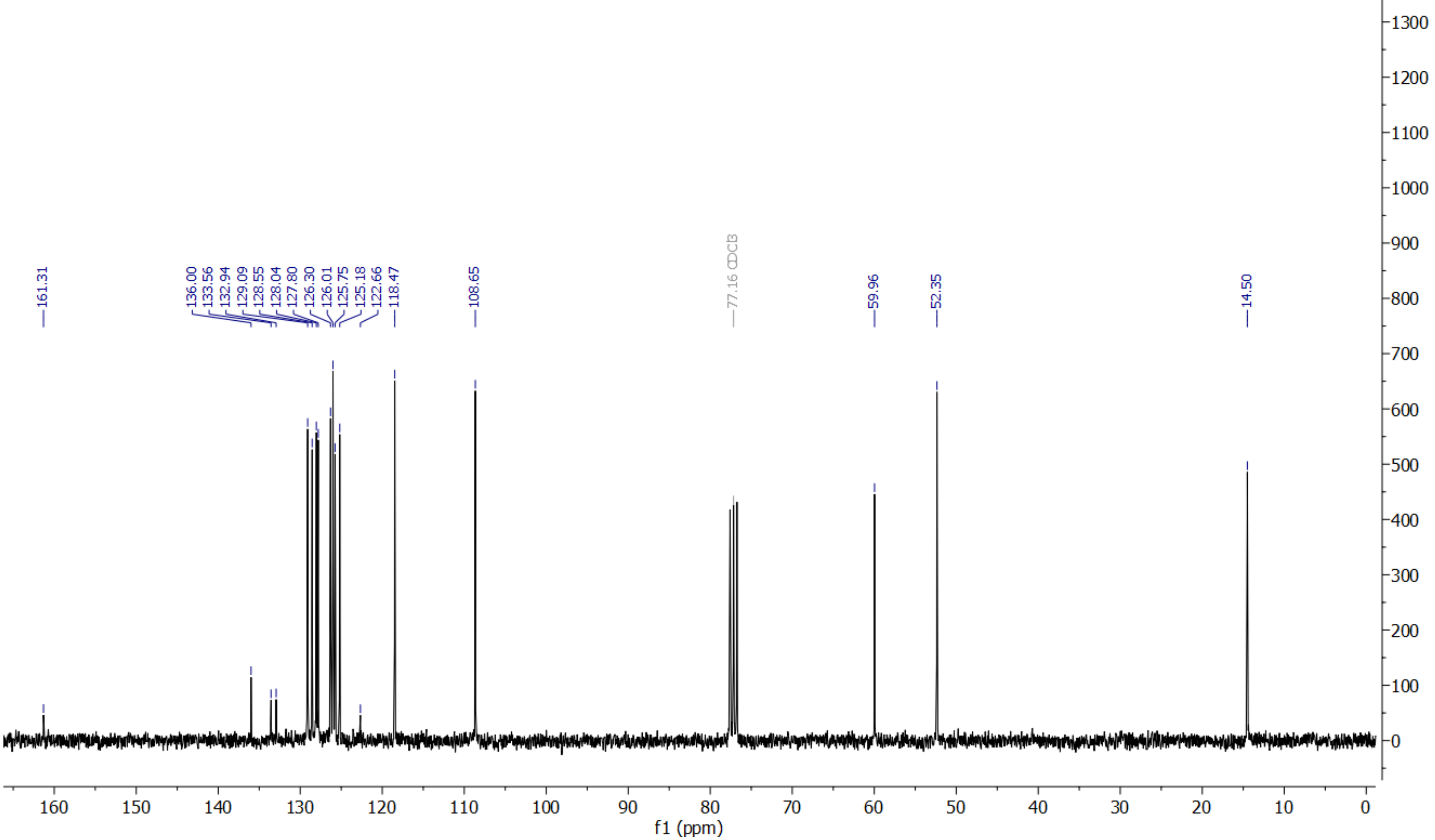

1-(naphthalen-1-ylmethyl)-1H-indole-2-carboxylic acid (2a)

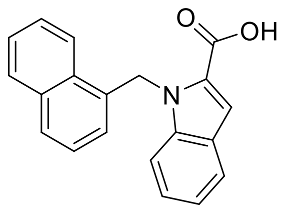

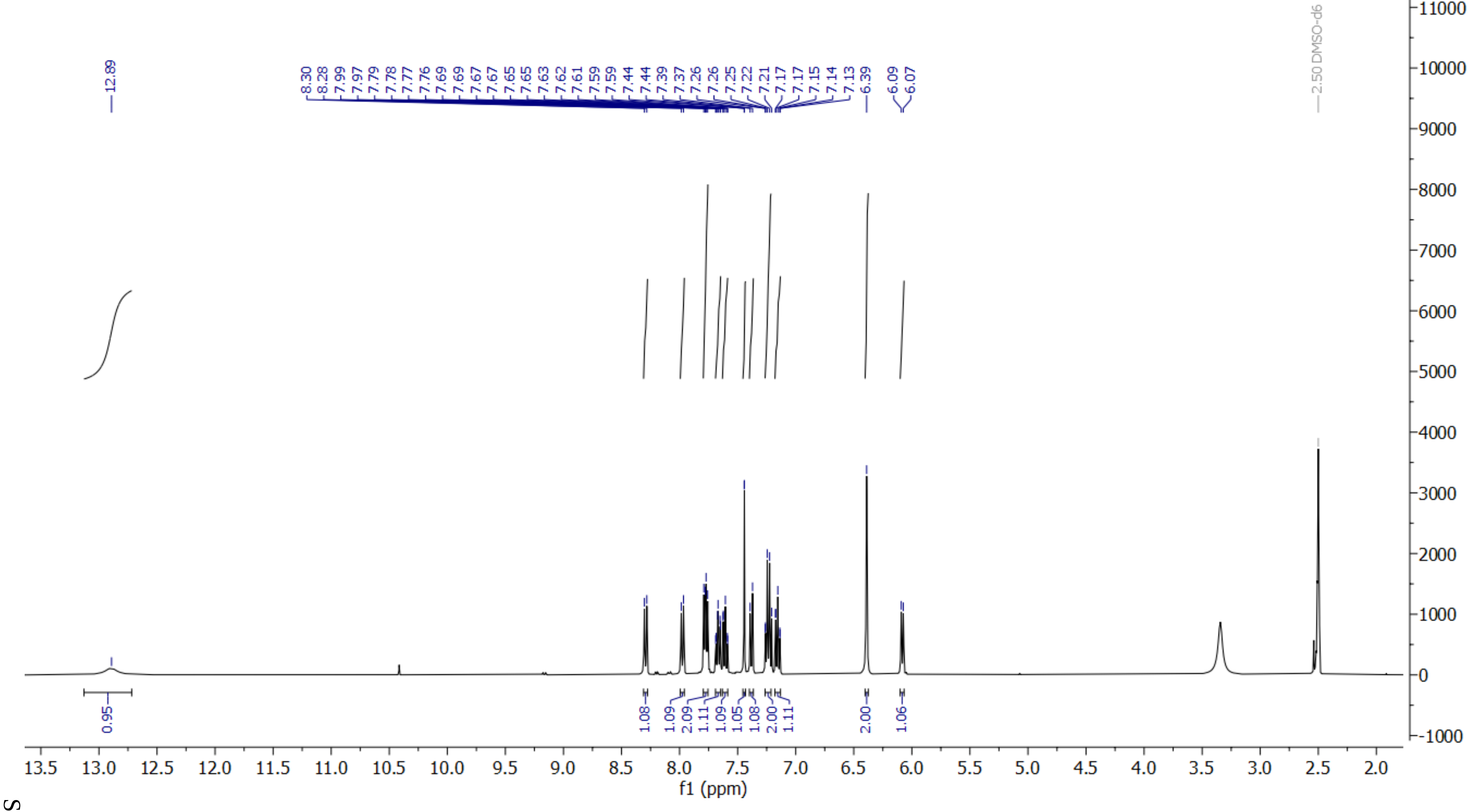

1-(naphthalen-1-ylmethyl)-1H-indole-2-carboxylic acid (2a)

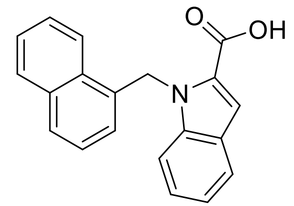

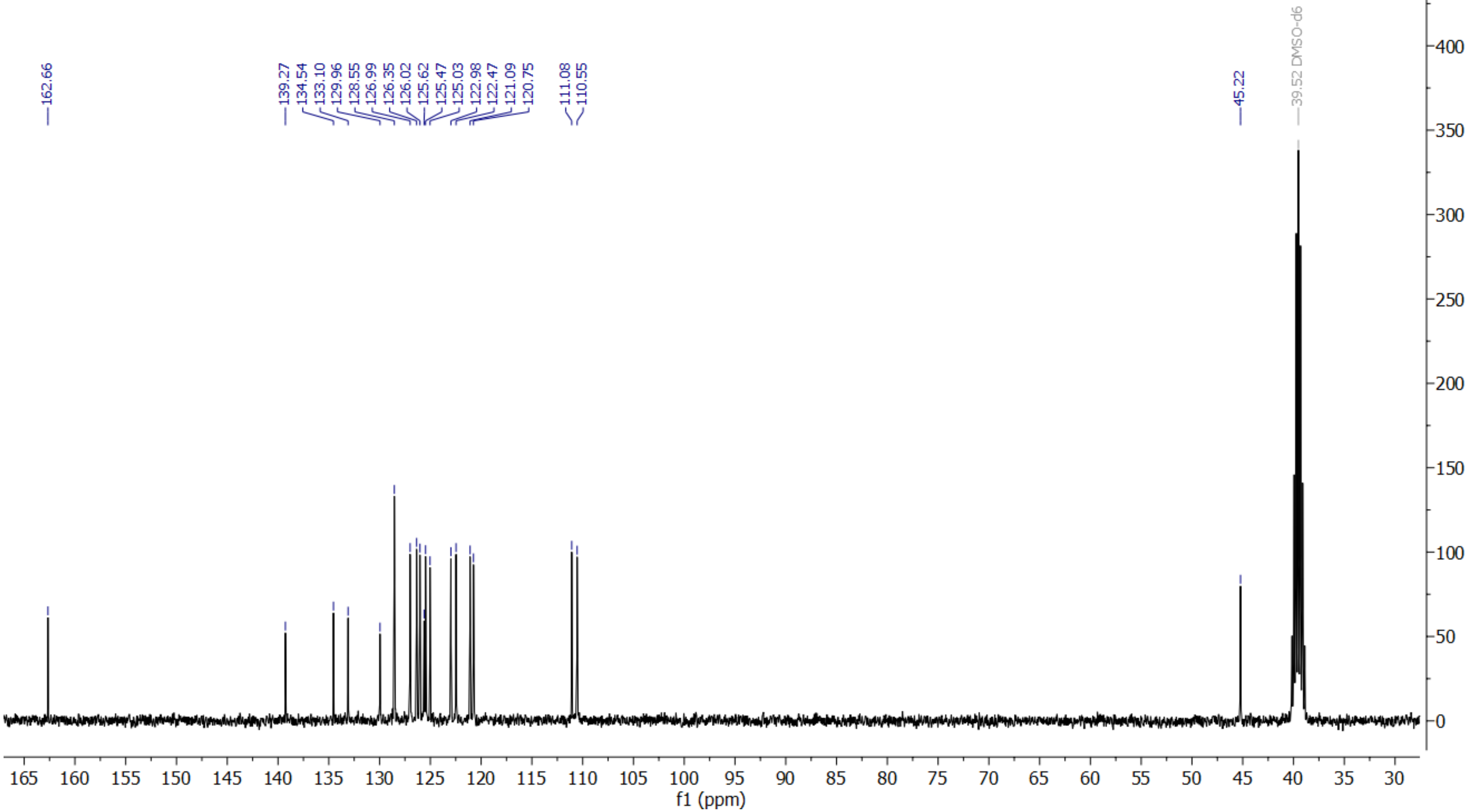

1-(naphthalen-2-ylmethyl)-1H-indole-2-carboxylic acid (2b)

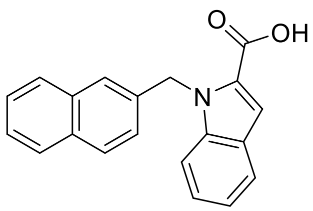

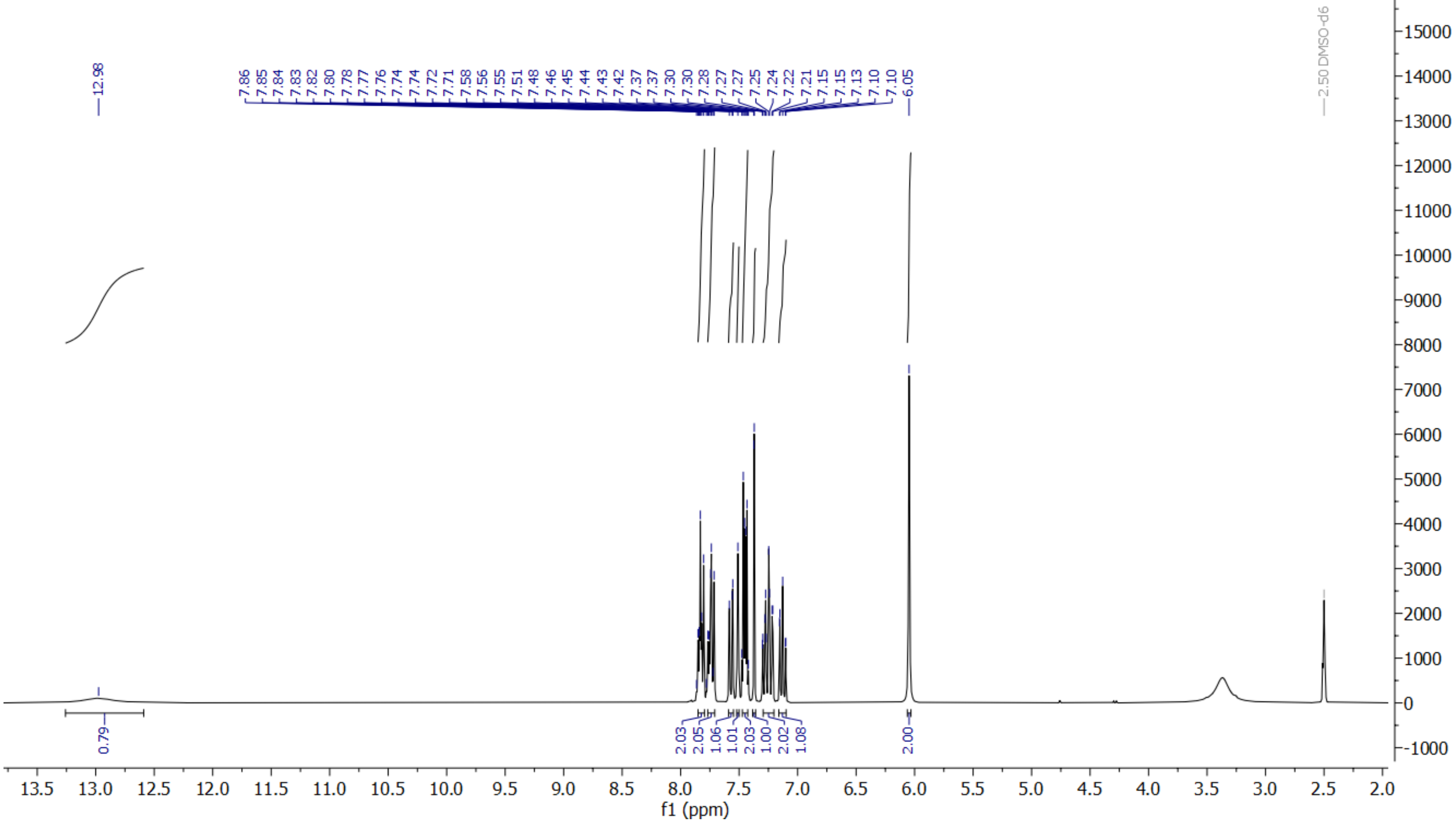

1-(naphthalen-2-ylmethyl)-1H-indole-2-carboxylic acid (2b)

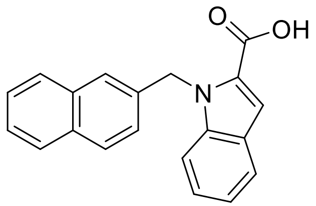

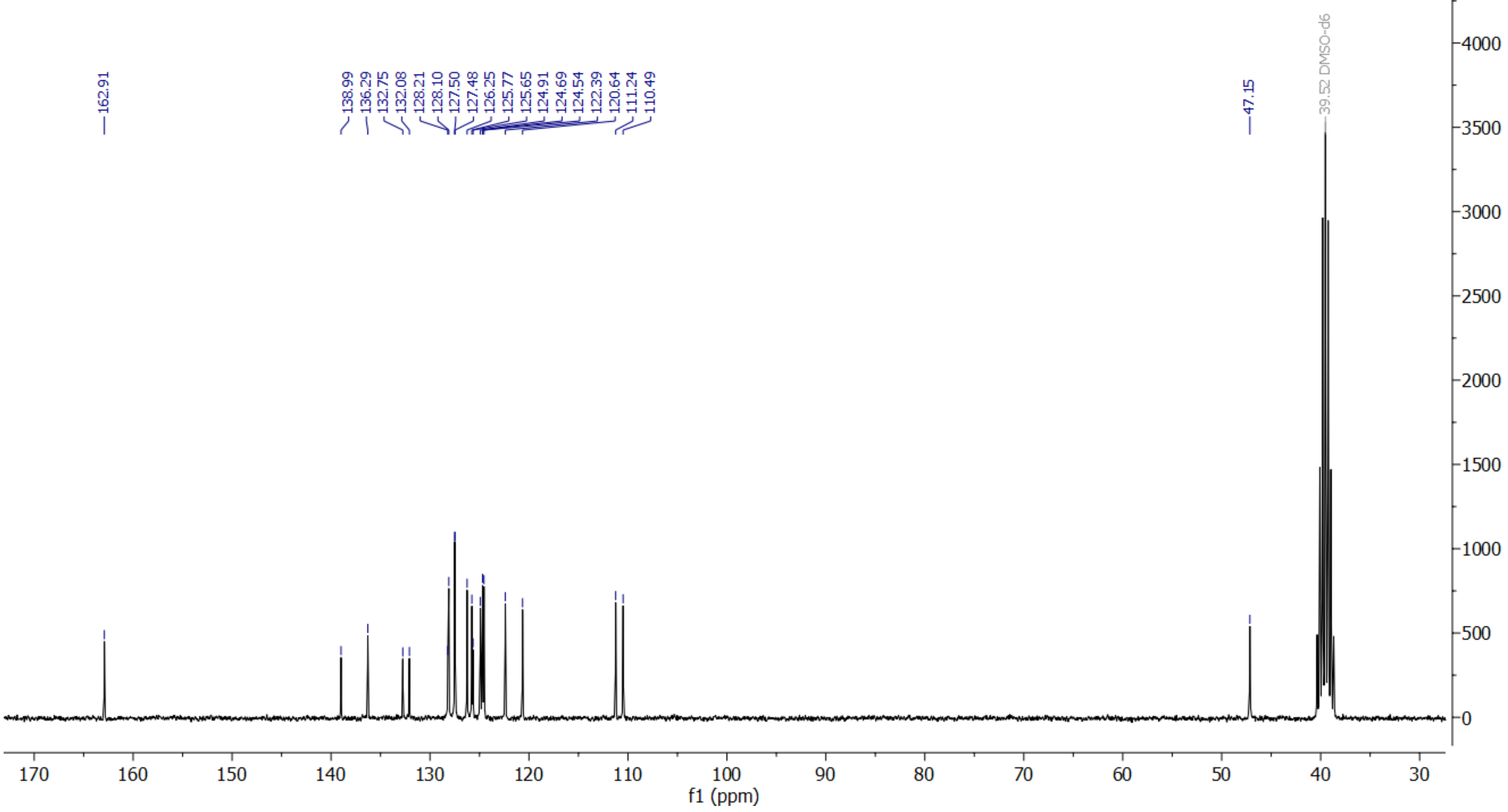

1-(naphthalen-2-ylmethyl)-1H-indole-6-carboxylic acid (3b)

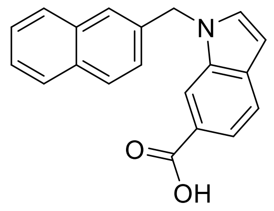

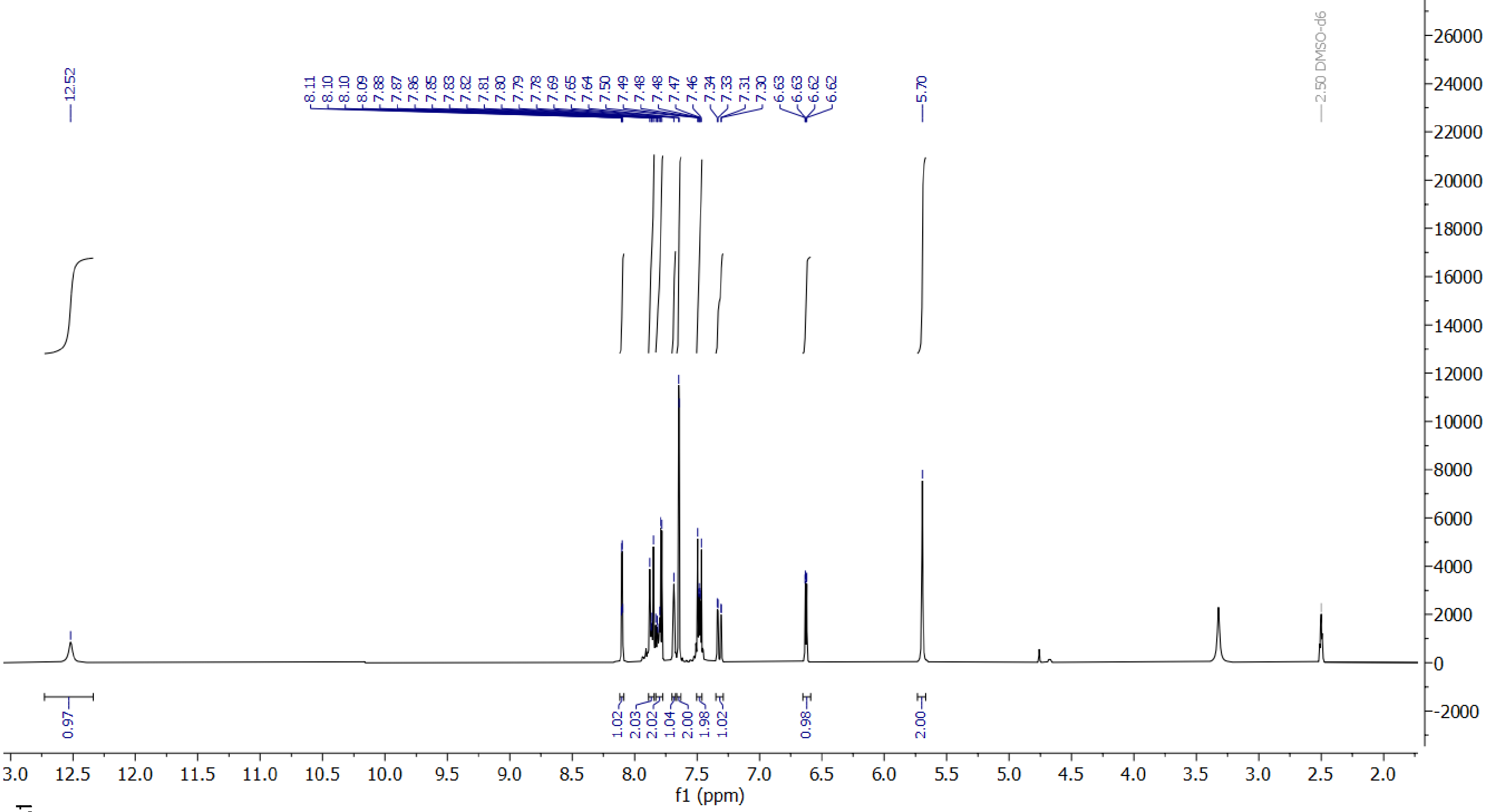

1-(naphthalen-2-ylmethyl)-1H-indole-6-carboxylic acid (3b)

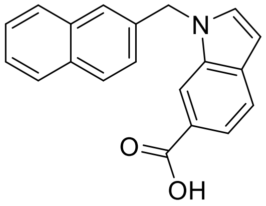

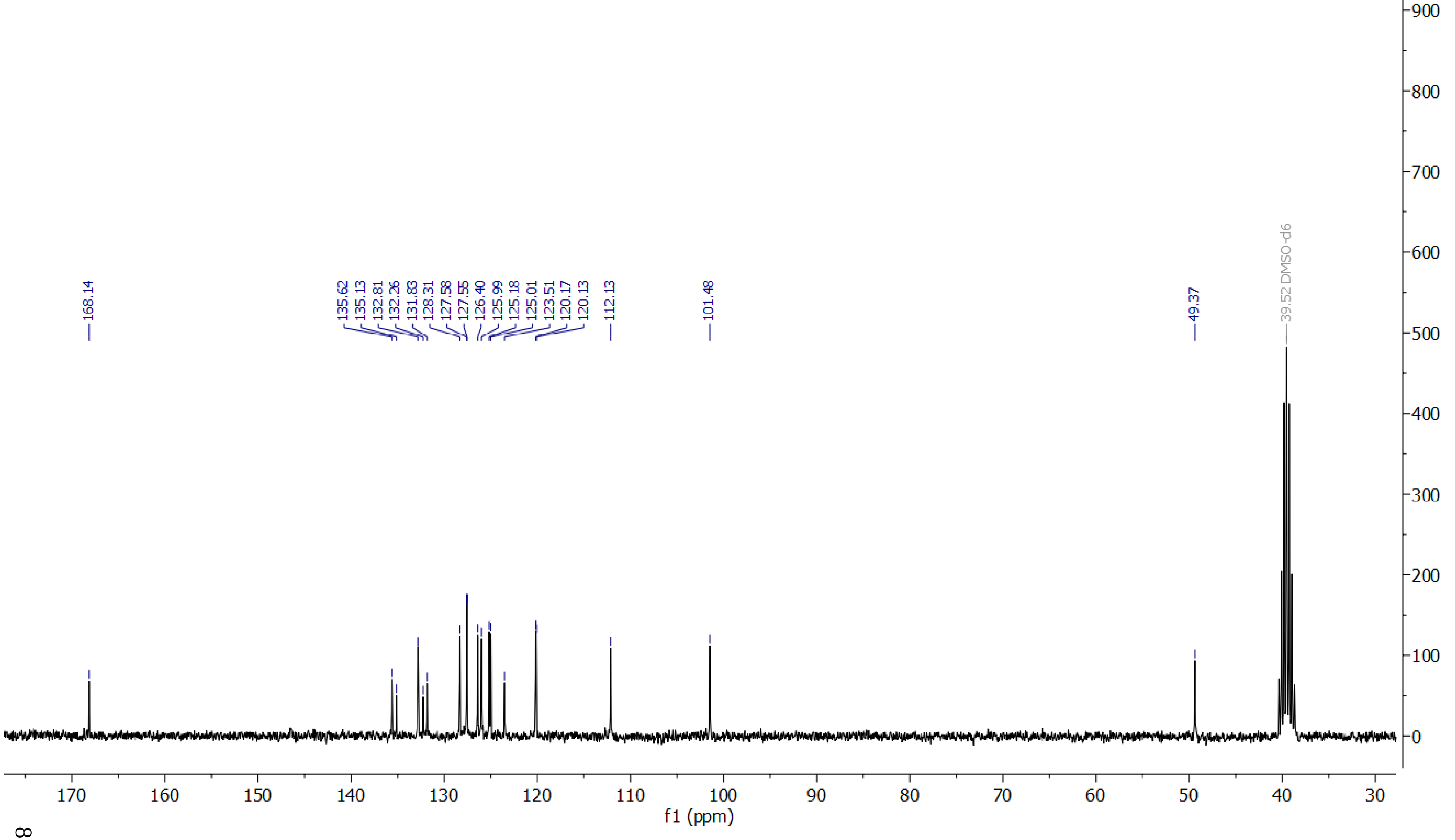

1-(naphthalen-1-ylmethyl)-1H-pyrrole-2-carboxylic acid (5a)

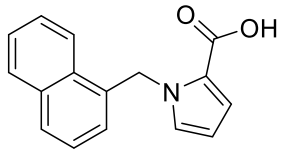

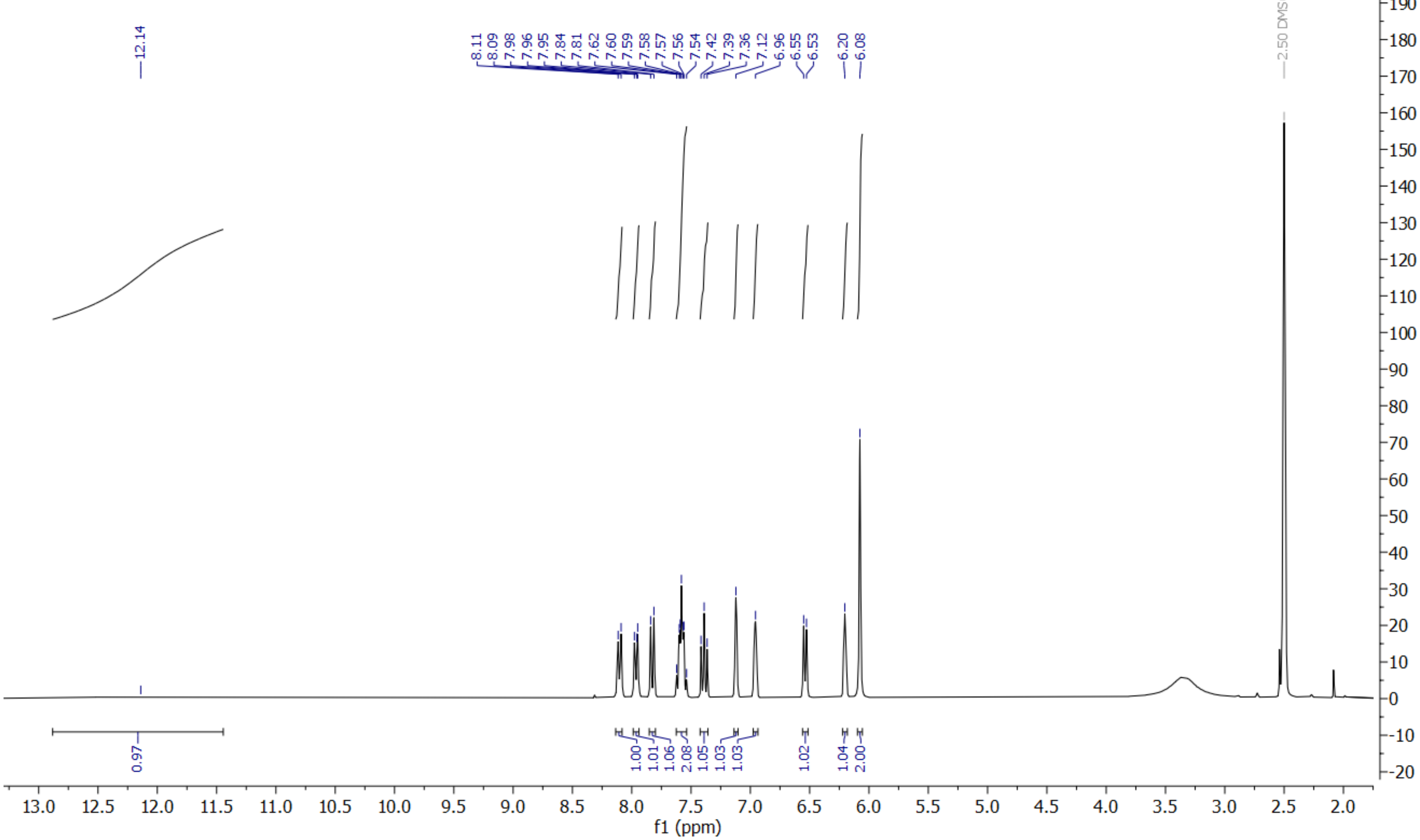

1-(naphthalen-1-ylmethyl)-1H-pyrrole-2-carboxylic acid (5a)

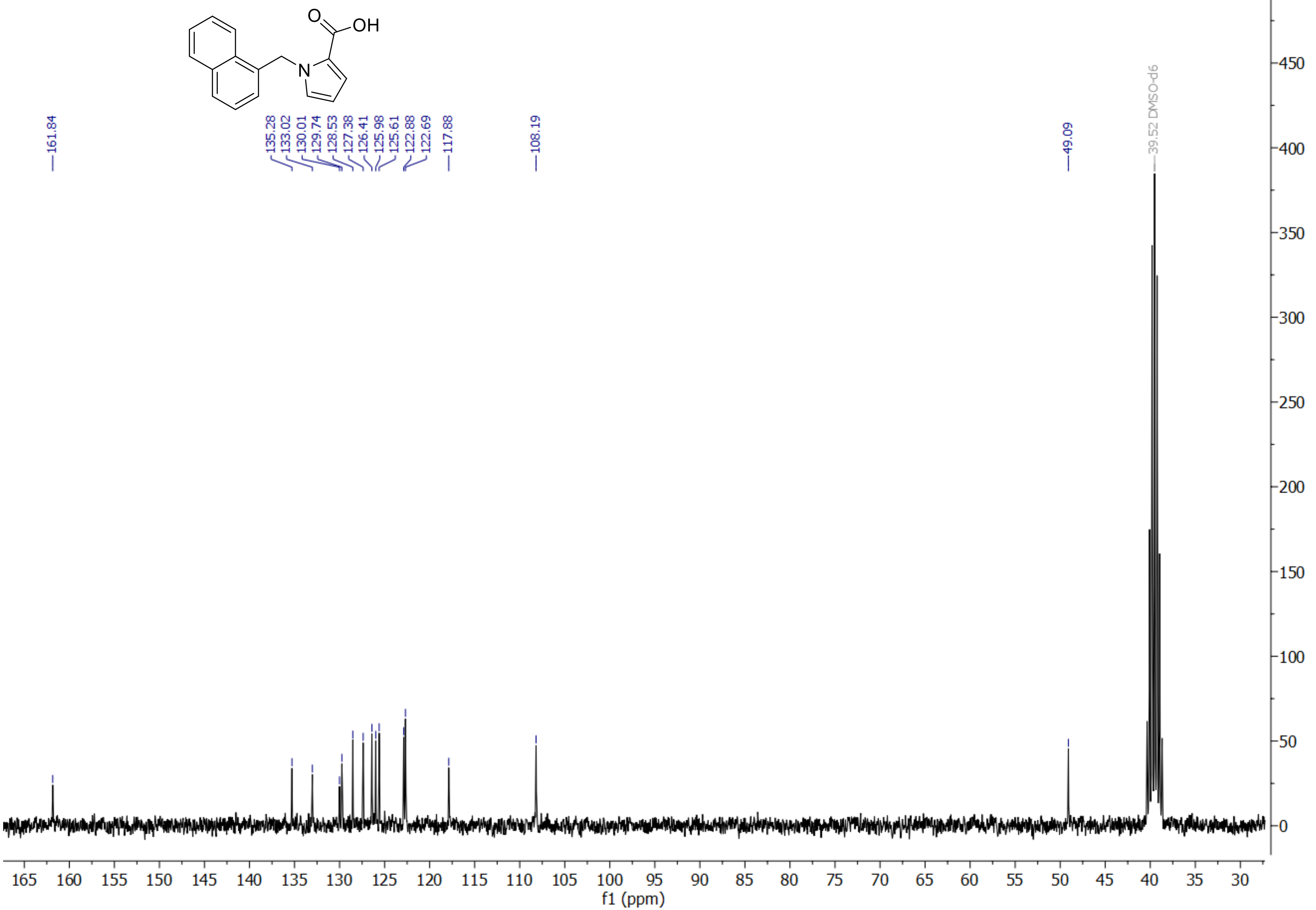

1-(naphthalen-2-ylmethyl)-1H-pyrrole-2-carboxylic acid (5b)

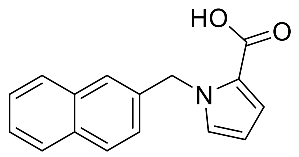

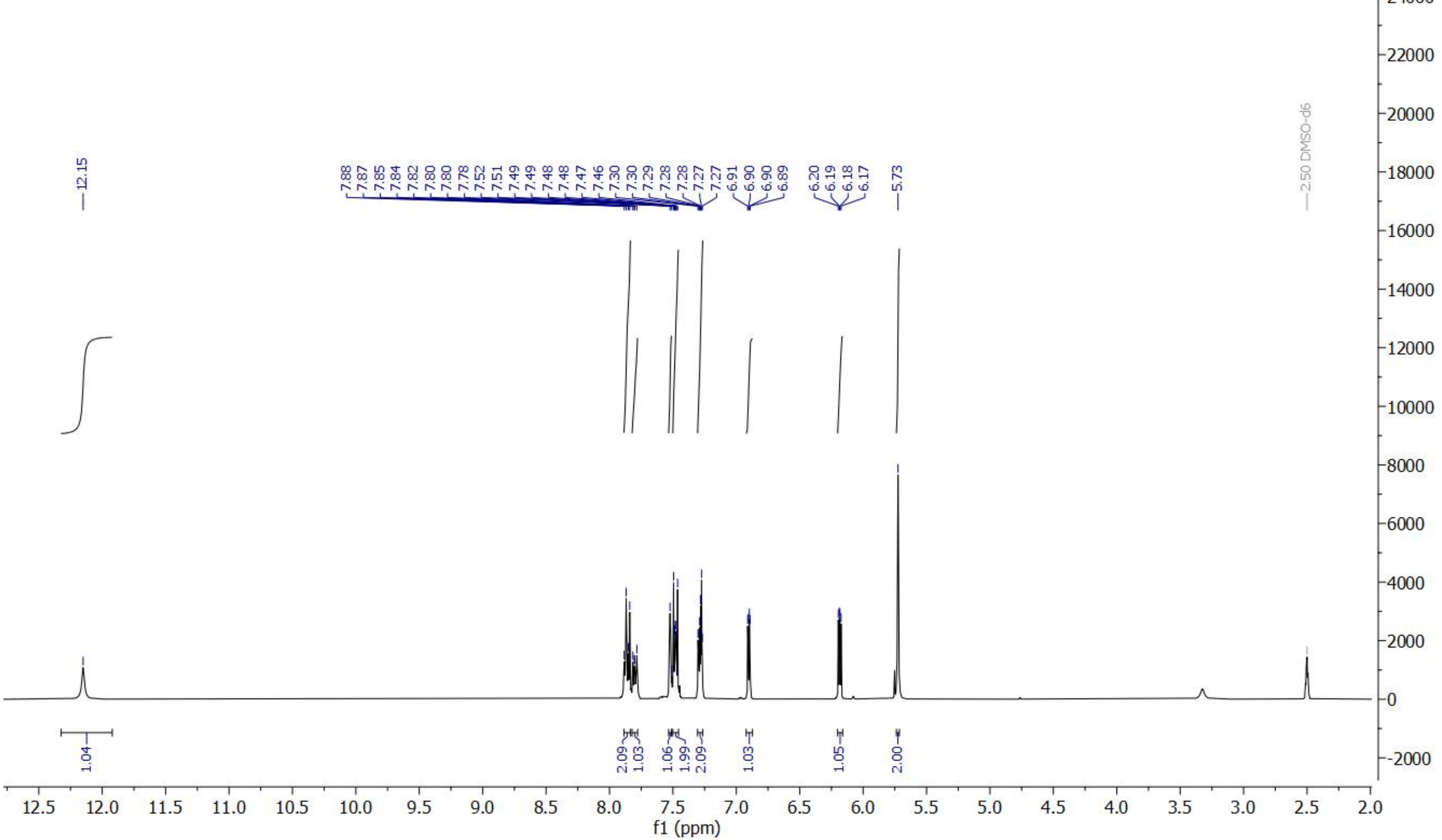

1-(naphthalen-2-ylmethyl)-1H-pyrrole-2-carboxylic acid (5b)

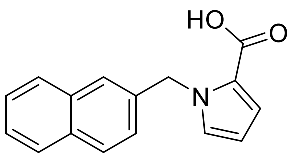

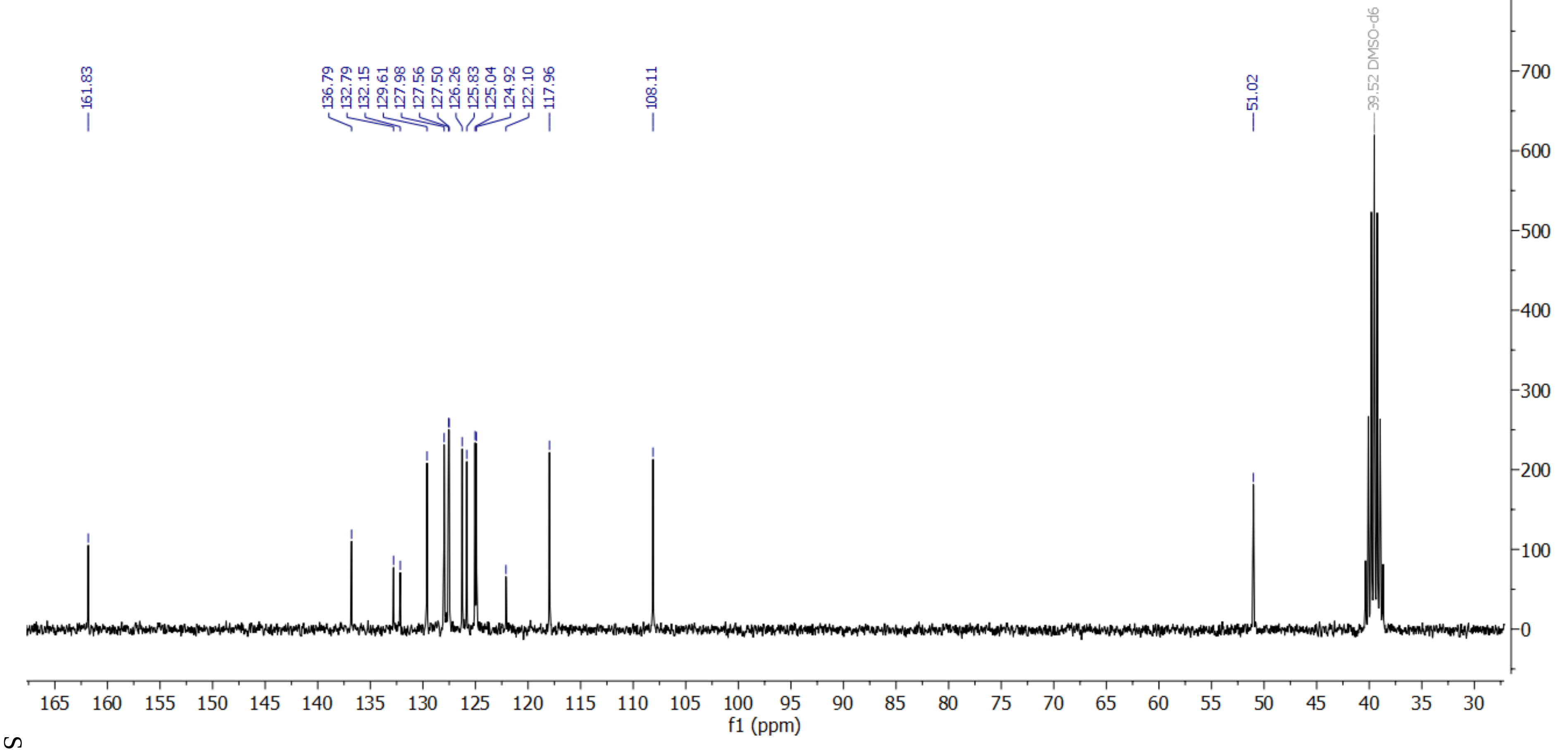

1-(naphthalen-1-ylmethyl)-1H-indole-3-carboxylic acid (1a)

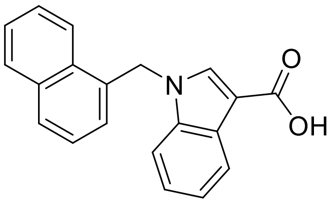

Injection Details

Injection Name:	**1a**
Instrument Method:	**10-90% 0.5 ml-min 6 min (MS) +FA**
Vial Number:	**G:D6**
Injection Date/Time:	**16/Jun/23 12:28**

Chromatogram

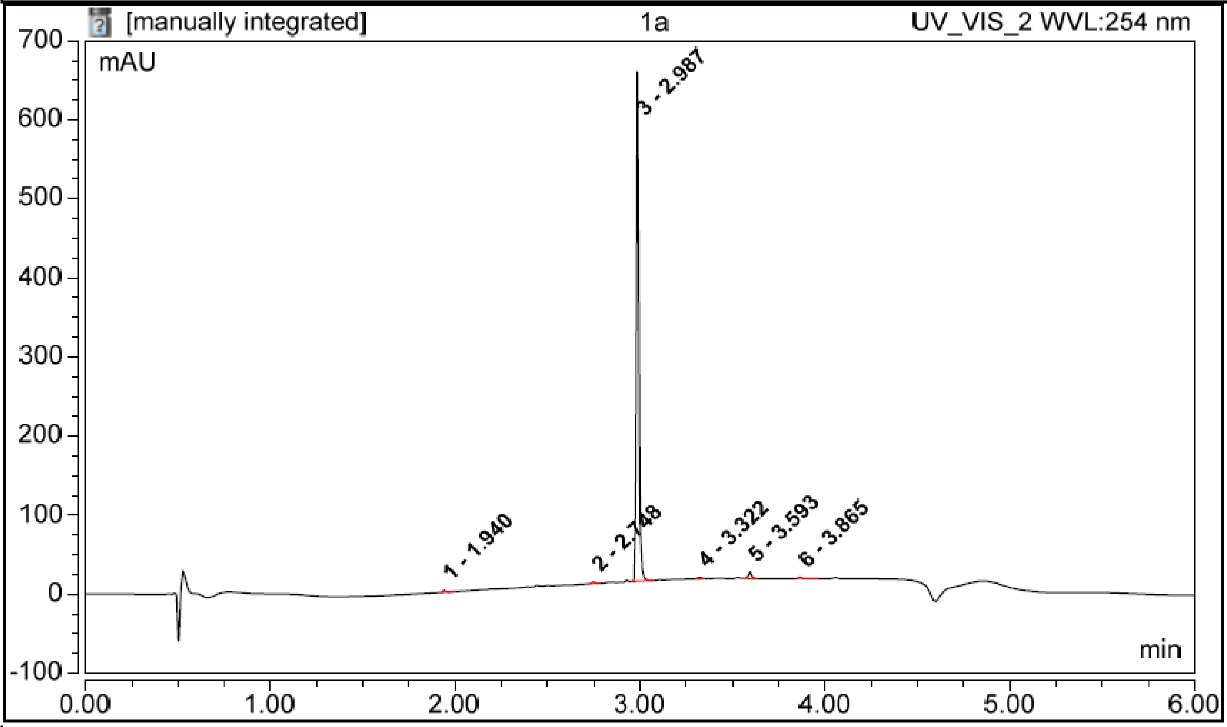

Integration Results

No.	Peak Name	Retention Time min	Area mAU*min	Relative Area %
1	1a	1.940	0.048	0.45
2		2.748	0.040	0.37
3		2.987	10.363	97.18
4		3.322	0.034	0.32
5		3.593	0.156	1.47
6		3.865	0.023	0.21
**Total:**			**10.665**	**100.00**

1-(naphthalen-2-ylmethyl)-1H-indole-3-carboxylic acid (1b)

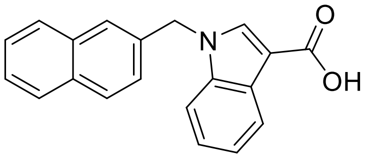

Injection Details	
Injection Name:	**1b**
Instrument Method:	**10-90% 0.5 ml-min 6 min (MS) +FA**
Vial Number:	**G:B2**
Injection Date/Time:	**29/May/23 22:33**

Chromatogram

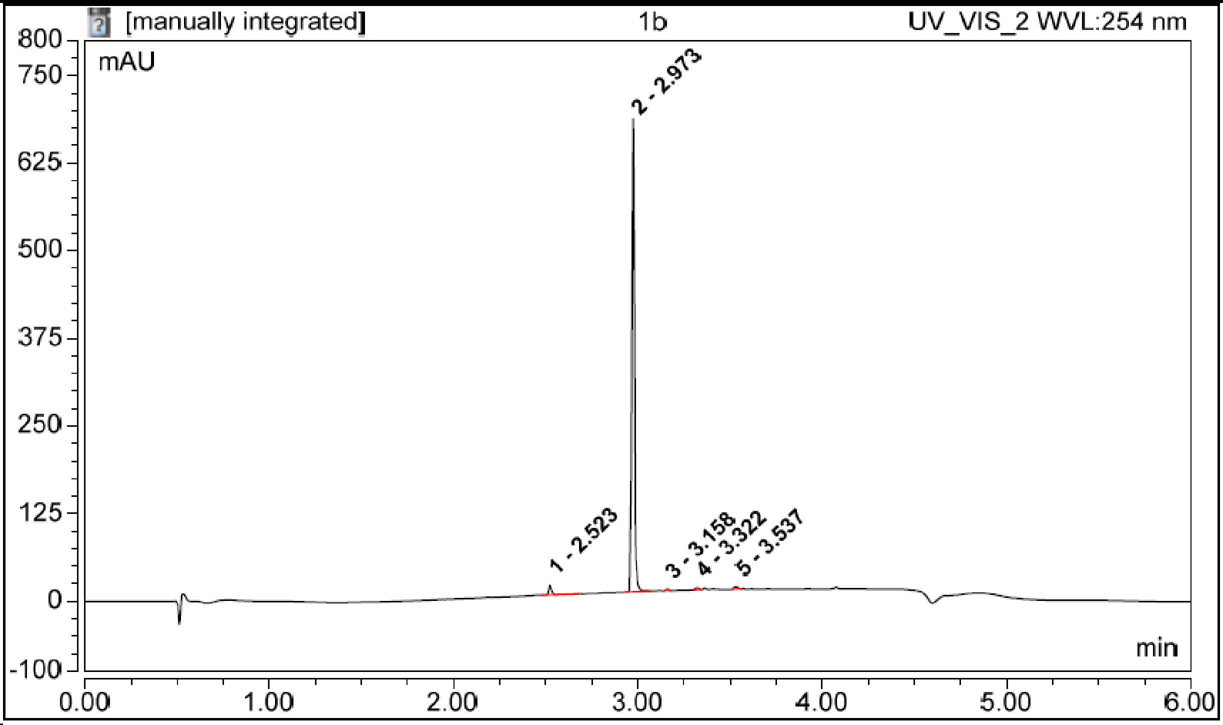

Integration Results

No.	Peak Name	Retention Time<br>min	Area<br>mAU*min	Relative Area<br>%
1		2.523	0.218	1.94
2	1b	2.973	10.809	96.44
3		3.158	0.041	0.37
4		3.322	0.054	0.49
5		3.537	0.085	0.76
**Total:**			**11.208**	**100.00**

Sodium 1-(naphthalen-1-ylmethyl)-1H-indole-3-carboxylate (1c)

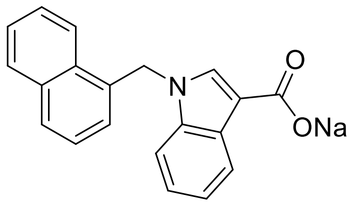

Injection Details

Injection Name:	**1c**
Instrument Method:	**10-90% 0.5 ml-min 6 min (MS) +FA**
Vial Number:	**G:A6**
Injection Date/Time:	**14/Apr/23 15:06**

Chromatogram

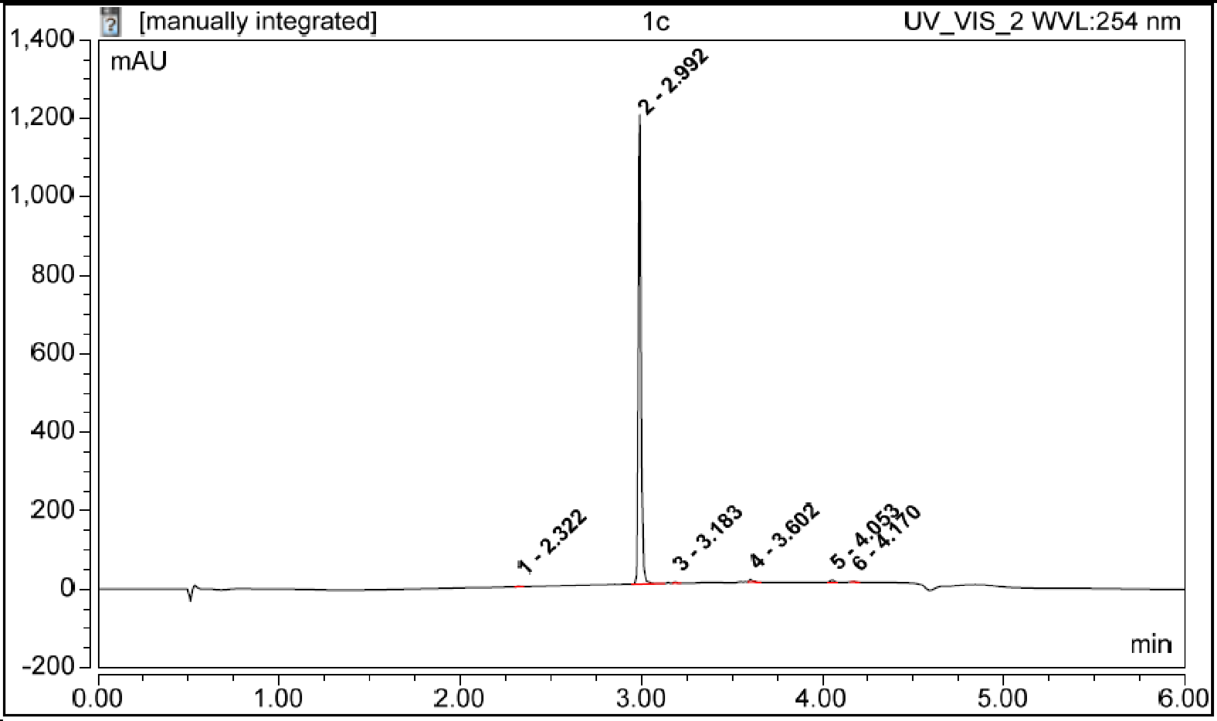

Integration Results

No.	Peak Name	Retention Time min	Area mAU*min	Relative Area %
1		2.322	0.019	0.09
2	1c	2.992	20.095	98.03
3		3.183	0.053	0.26
4		3.602	0.156	0.76
5		4.053	0.133	0.65
6		4.170	0.041	0.20
**Total:**			**20.498**	**100.00**

1-(naphthalen-1-ylmethyl)-1H-indole-2-carboxylic acid (2a)

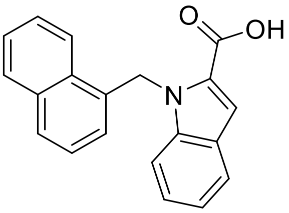

Injection Details

Injection Name:	**2a**
Instrument Method:	**10-90% 0.5 ml-min 6 min (MS) +FA**
Vial Number:	**G:A8**
Injection Date/Time:	**18/Jul/23 14:57**

Chromatogram

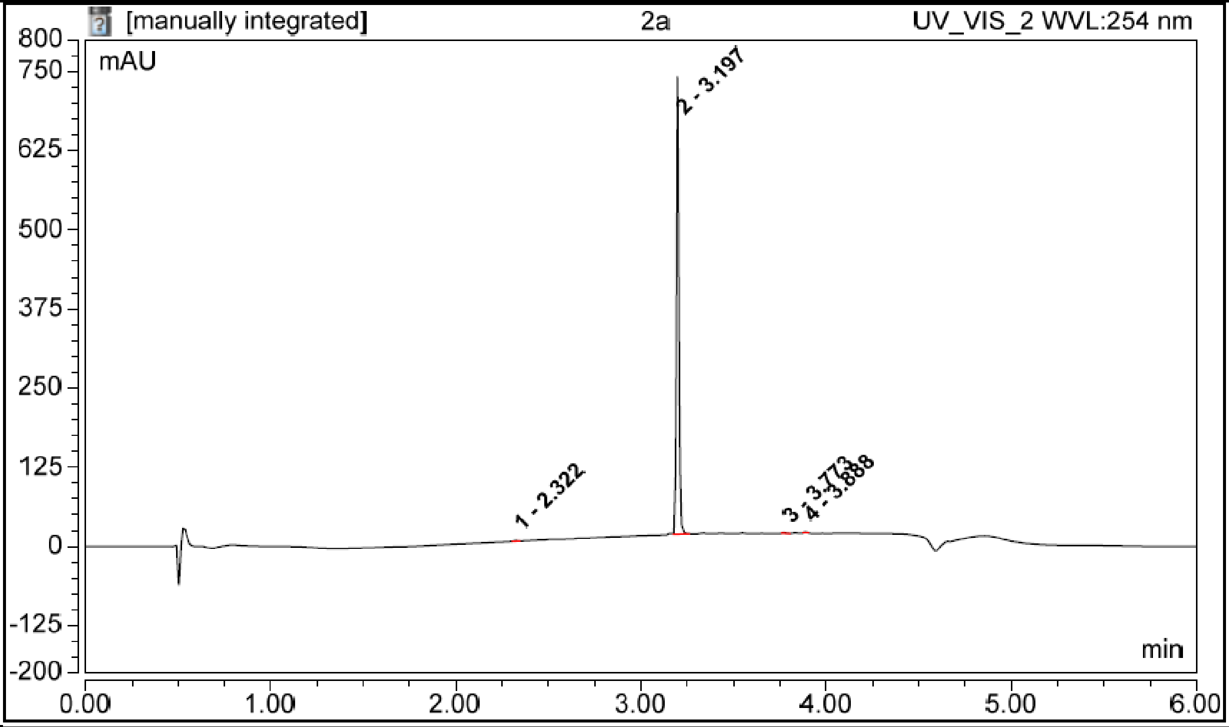

Integration Results

No.	Peak Name	Retention Time<br>min	Area<br>mAU*min	Relative Area<br>%
1		2.322	0.021	0.18
2	2a	3.197	11.986	99.36
3		3.773	0.024	0.20
4		3.888	0.032	0.27
**Total:**			**12.063**	**100.00**

1-(naphthalen-2-ylmethyl)-1H-indole-2-carboxylic acid (2b)

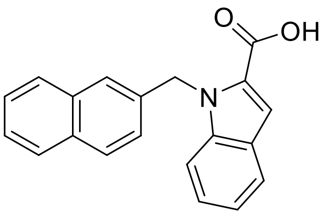

Injection Details

Injection Name:	**2b**
Instrument Method:	**10-90% 0.5 ml-min 6 min (MS) +FA**
Vial Number:	**G:D5**
Injection Date/Time:	**20/Jun/23 16:25**

Chromatogram

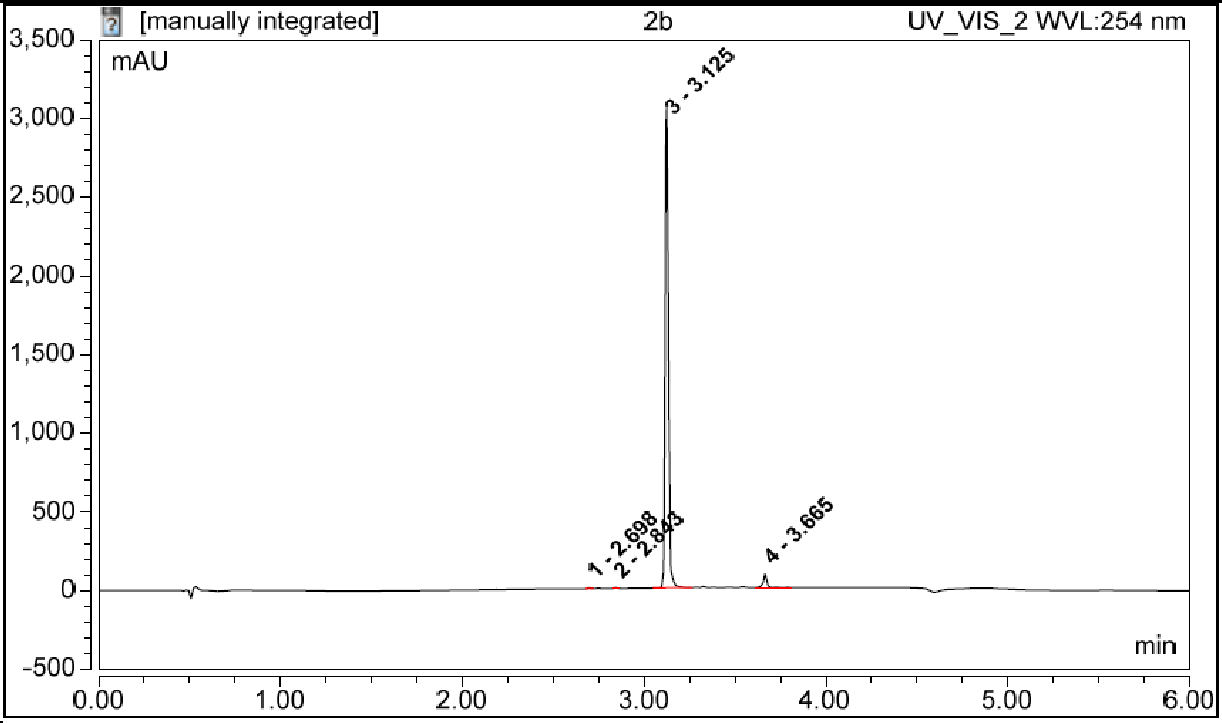

Integration Results

No.	Peak Name	Retention Time min	Area mAU*min	Relative Area %
1		2.698	0.118	0.18
2		2.843	0.082	0.12
3	2b	3.125	65.085	97.12
4		3.665	1.731	2.58
**Total:**			**67.017**	**100.00**

1-(naphthalen-1-ylmethyl)-1H-indole-5-carboxylic acid (3a)

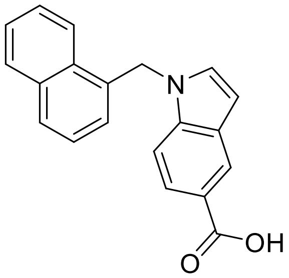

Injection Details

Injection Name:	**3a**
Instrument Method:	**10-90% 0.5 ml-min 6 min (MS) +FA**
Vial Number:	**G:A5**
Injection Date/Time:	**16/Aug/23 15:14**

Chromatogram

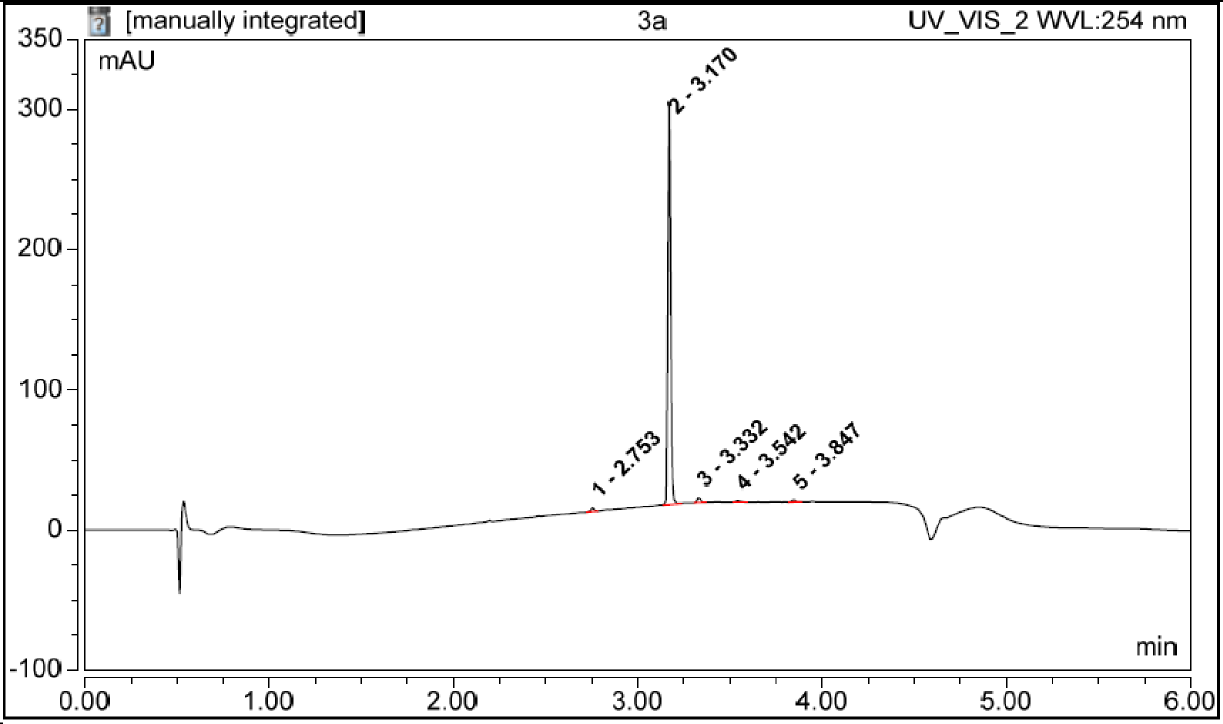

Integration Results

No.	Peak Name	Retention Time min	Area mAU*min	Relative Area %
1		2.753	0.052	1.07
2	3a	3.170	4.720	96.29
3		3.332	0.069	1.41
4		3.542	0.026	0.52
5		3.847	0.035	0.71
**Total:**			**4.901**	**100.00**

1-(naphthalen-2-ylmethyl)-1H-indole-6-carboxylic acid (3b)

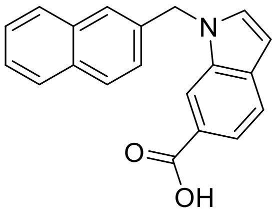

Injection Details

Injection Name:	**3b**
Instrument Method:	**10-90% 0.5 ml-min 6 min (MS) +FA**
Vial Number:	**G:C9**
Injection Date/Time:	**19/Jul/23 18:25**

Chromatogram

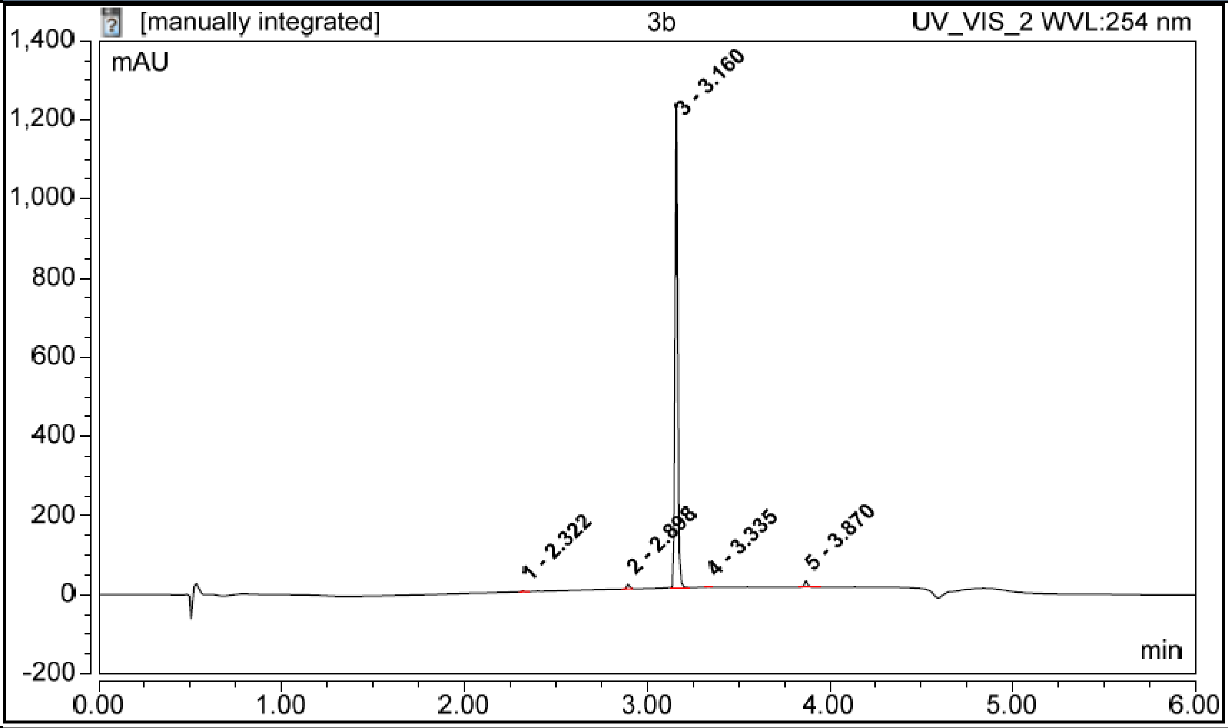

Integration Results

No.	Peak Name	Retention Time<br>min	Area<br>mAU*min	Relative Area<br>%
1		2.322	0.033	0.16
2		2.898	0.224	1.07
3	3b	3.160	20.295	97.16
4		3.335	0.018	0.09
5		3.870	0.318	1.52
**Total:**			**20.889**	**100.00**

1-(naphthalen-1-ylmethyl)-1H-indole-6-carboxylic acid (4a)

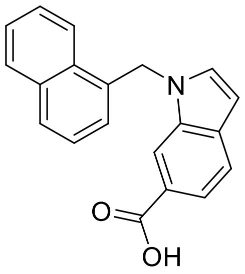

Injection Details

Injection Name:	**4a**
Instrument Method:	**10-90% 0.5 ml-min 6 min (MS) +FA**
Vial Number:	**G:C6**
Injection Date/Time:	**18/Apr/23 20:02**

Chromatogram

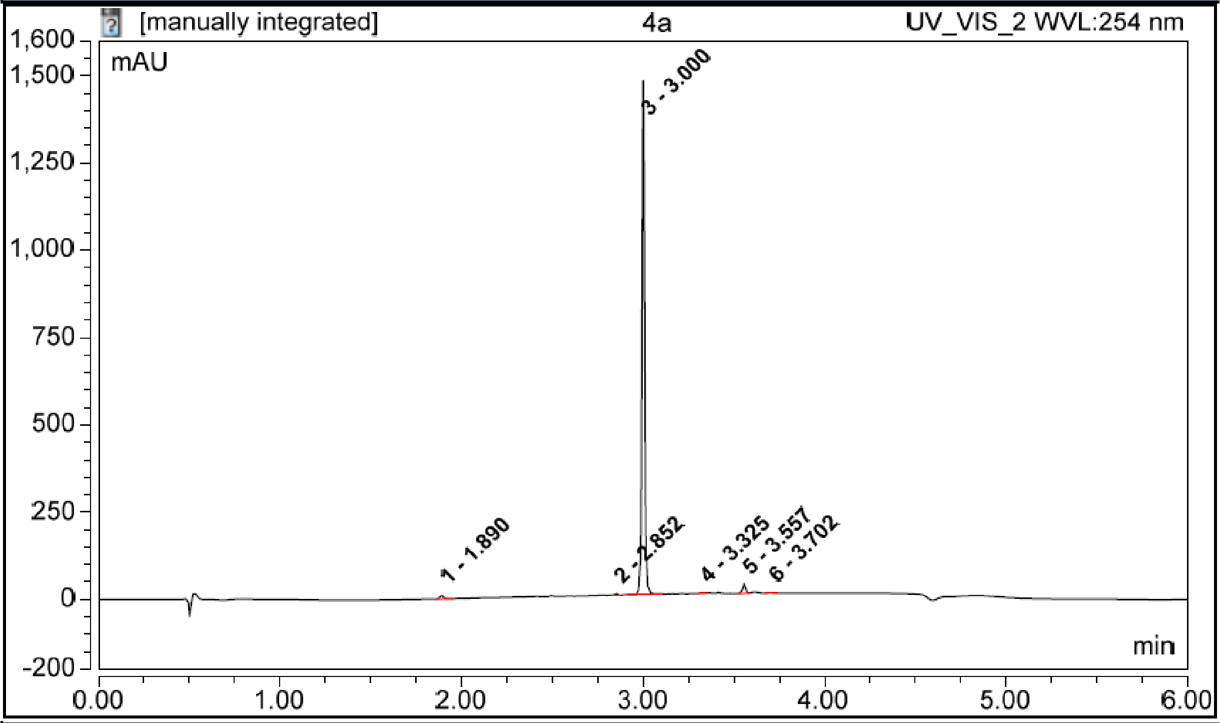

Integration Results

No.	Peak Name	Retention Time min	Area mAU*min	Relative Area %
1	4a	1.890	0.218	0.86
2		2.852	0.077	0.31
3		3.000	24.329	96.51
4		3.325	0.030	0.12
5		3.557	0.490	1.94
6		3.702	0.064	0.25
**Total:**			**25.208**	**100.00**

1-(naphthalen-2-ylmethyl)-1H-indole-5-carboxylic acid (4b)

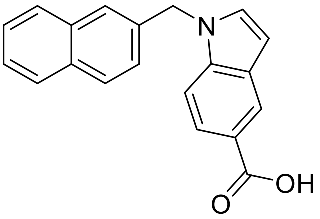

Injection Details

Injection Name:	**4b**
Instrument Method:	**10-90% 0.5 ml-min 6 min (MS) +FA**
Vial Number:	**G:B4**
Injection Date/Time:	**29/May/23 22:51**

Chromatogram

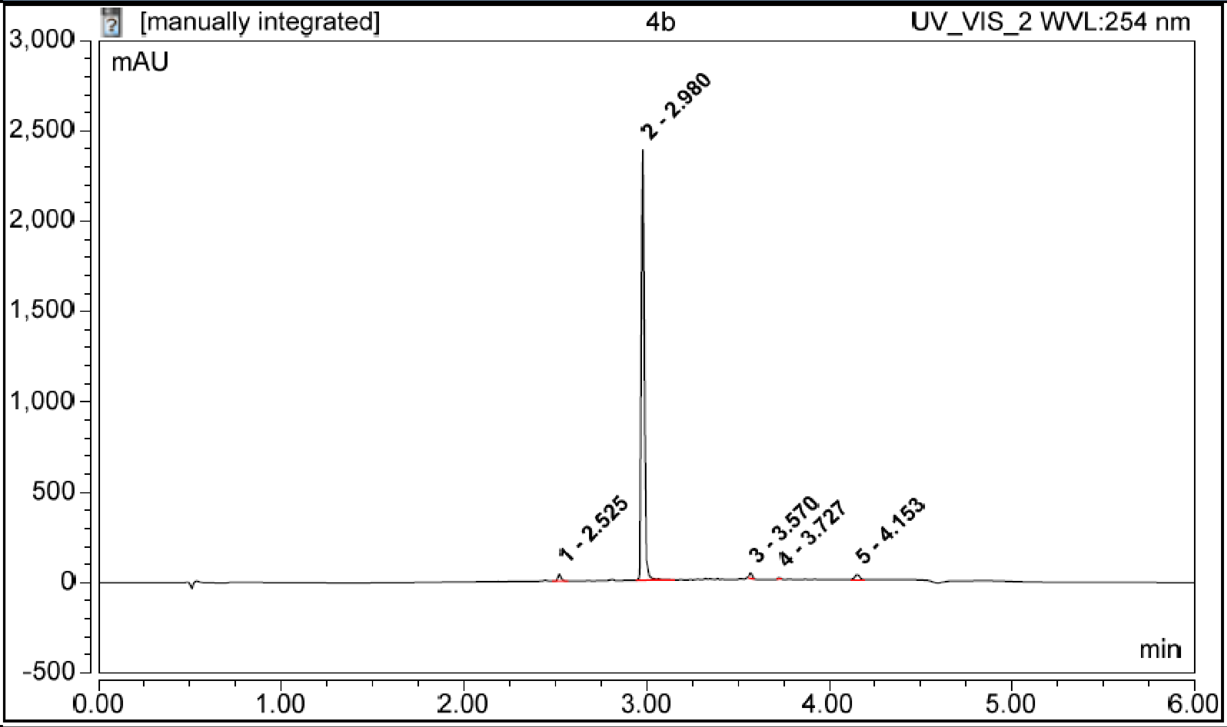

Integration Results

No.	Peak Name	Retention Time min	Area mAU*min	Relative Area %
1		2.525	0.670	1.49
2	4b	2.980	43.050	95.51
3		3.570	0.511	1.13
4		3.727	0.136	0.30
5		4.153	0.708	1.57
**Total:**			**45.076**	**100.00**

1-(naphthalen-1-ylmethyl)-1H-pyrrole-2-carboxylic acid (5a)

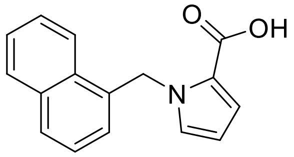

Injection Details

Injection Name:	**5a**
Instrument Method:	**10-90% 0.5 ml-min 6 min (MS) +FA**
Vial Number:	**G:B8**
Injection Date/Time:	**15/Jun/23 11:29**

Chromatogram

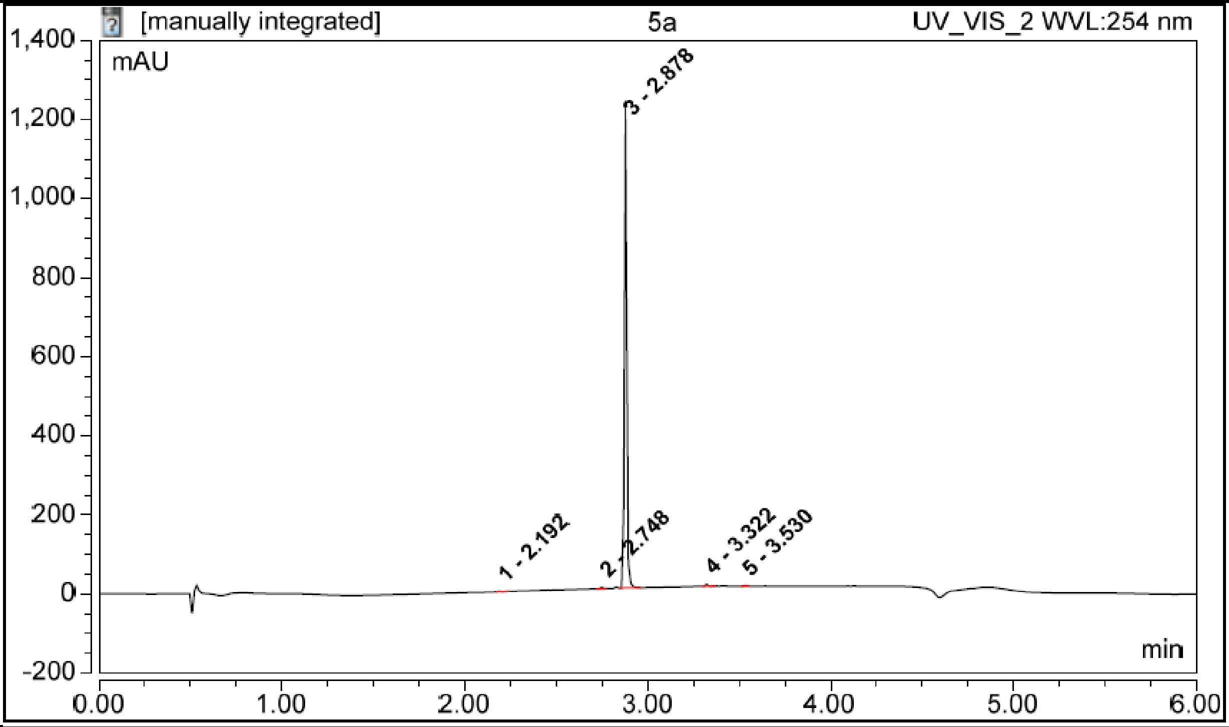

Integration Results

No.	Peak Name	Retention Time min	Area mAU*min	Relative Area %
1		2.192	0.015	0.07
2		2.748	0.063	0.31
3	5a	2.878	19.730	99.11
4		3.322	0.075	0.38
5		3.530	0.024	0.12
**Total:**			**19.907**	**100.00**

1-(naphthalen-2-ylmethyl)-1H-pyrrole-2-carboxylic acid (5b)

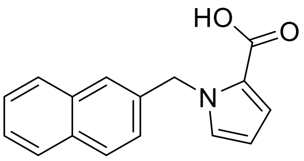

Injection Details

Injection Name:	**5b**
Instrument Method:	**10-90% 0.5 ml-min 6 min (MS) +FA**
Vial Number:	**G:A5**
Injection Date/Time:	**23/May/23 12:10**

Chromatogram

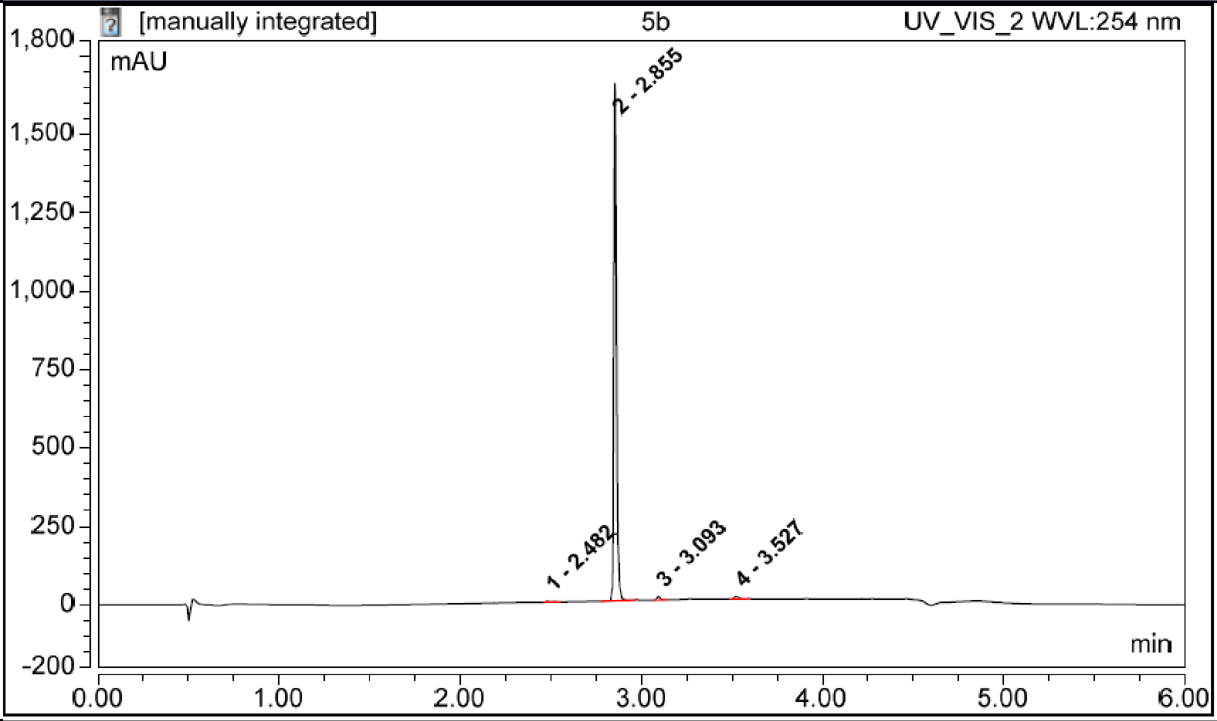

Integration Results

No.	Peak Name	Retention Time min	Area mAU*min	Relative Area %
1		2.482	0.072	0.27
2	5b	2.855	26.584	98.14
3		3.093	0.206	0.76
4		3.527	0.227	0.84
**Total:**			**27.089**	**100.00**

References

23. McWilliam, H.; Li, W.; Uludag, M.; Squizzato, S.; Park, Y.M.; Buso, N.; Cowley, A.P.; Lopez, R. Analysis Tool Web Services from the EMBL-EBI. *Nucleic Acids Res.* **2013**, *41*, W597–W600, doi:10.1093/nar/gkt376.

54. Subhas Bose, D.; Idrees, M.; Todewale, I.K.; Jakka, N.M.; Venkateswara Rao, J. Hybrids of Privileged Structures Benzothiazoles and Pyrrolo[2,1-c] [1,4]Benzodiazepin-5-One, and Diversity-Oriented Synthesis of Benzothiazoles. *Eur.J. Med. Chem.* **2012**, *50*, 27–38, doi:10.1016/j.ejmech.2012.01.014.

55. Yang, T.; Li, X.; Deng, S.; Qi, X.; Cong, H.; Cheng, H.-G.; Shi, L.; Zhou, Q.; Zhuang, L. From N–H Nitration to Controllable Aromatic Mononitration and Dinitration─The Discovery of a Versatile and Powerful *N* -Nitropyrazole Nitrating Reagent. *JACS Au* **2022**, *2*, 2152–2161, doi:10.1021/jacsau.2c00413.

56. Brown, F.J.; Cronk, L.A.; Aharony, D.; Snyder, D.W. 1,3,6-Trisubstituted Indoles as Peptidoleukotriene Antagonists: Benefits of a Second, Polar, Pyrrole Substituent. *J. Med. Chem.* **1992**, *35*, 2419–2439, doi:10.1021/jm00091a010.

57. Houck, H.A.; Blasco, E.; Du Prez, F.E.; Barner-Kowollik, C. Light-Stabilized Dynamic Materials. *J. Am. Chem. Soc.* **2019**, *141*, 12329–12337, doi:10.1021/jacs.9b05092.

58. Young, B.M.; Rossi, P.; Slavish, P.J.; Cui, Y.; Sowaileh, M.; Das, J.; Kalodimos, C.G.; Rankovic, Z. Synthesis of Isotopically Labeled, Spin-Isolated Tyrosine and Phenylalanine for Protein NMR Applications. *Org. Lett.* **2021**, *23*, 6288–6292, doi:10.1021/acs.orglett.1c02084.

59. Verma, A.K.; Fatima, K.; Dudi, R.K.; Tabassum, M.; Iqbal, H.; Kumar, Y.; Luqman, S.; Mondhe, D.M.; Chanda, D.; Khan, F.; et al. Antiproliferative Activity of Diarylnaphthylpyrrolidine Derivative via Dual Target Inhibition. *Eur. J. Med. Chem.* **2020**, *188*, doi:10.1016/j.ejmech.2019.111986.

60. Portolani, C.; Centonze, G.; Luciani, S.; Pellegrini, A.; Righi, P.; Mazzanti, A.; Ciogli, A.; Sorato, A.; Bencivenni, G. Synthesis of Atropisomeric Hydrazides by One-Pot Sequential Enantio- and Diastereoselective Catalysis. *Angewandte Chemie International Edition* **2022**, *61,* doi:10.1002/anie.202209895.
